# Supplementary figures and images for: Magnesium isoglycyrrhizinate alleviates alcohol-associated liver disease through targeting HSD11B1
Source: eLife. 2026 Jul 28;15:RP109174. doi: 10.7554/eLife.109174 (PMC13412320; doi:10.7554/eLife.109174)

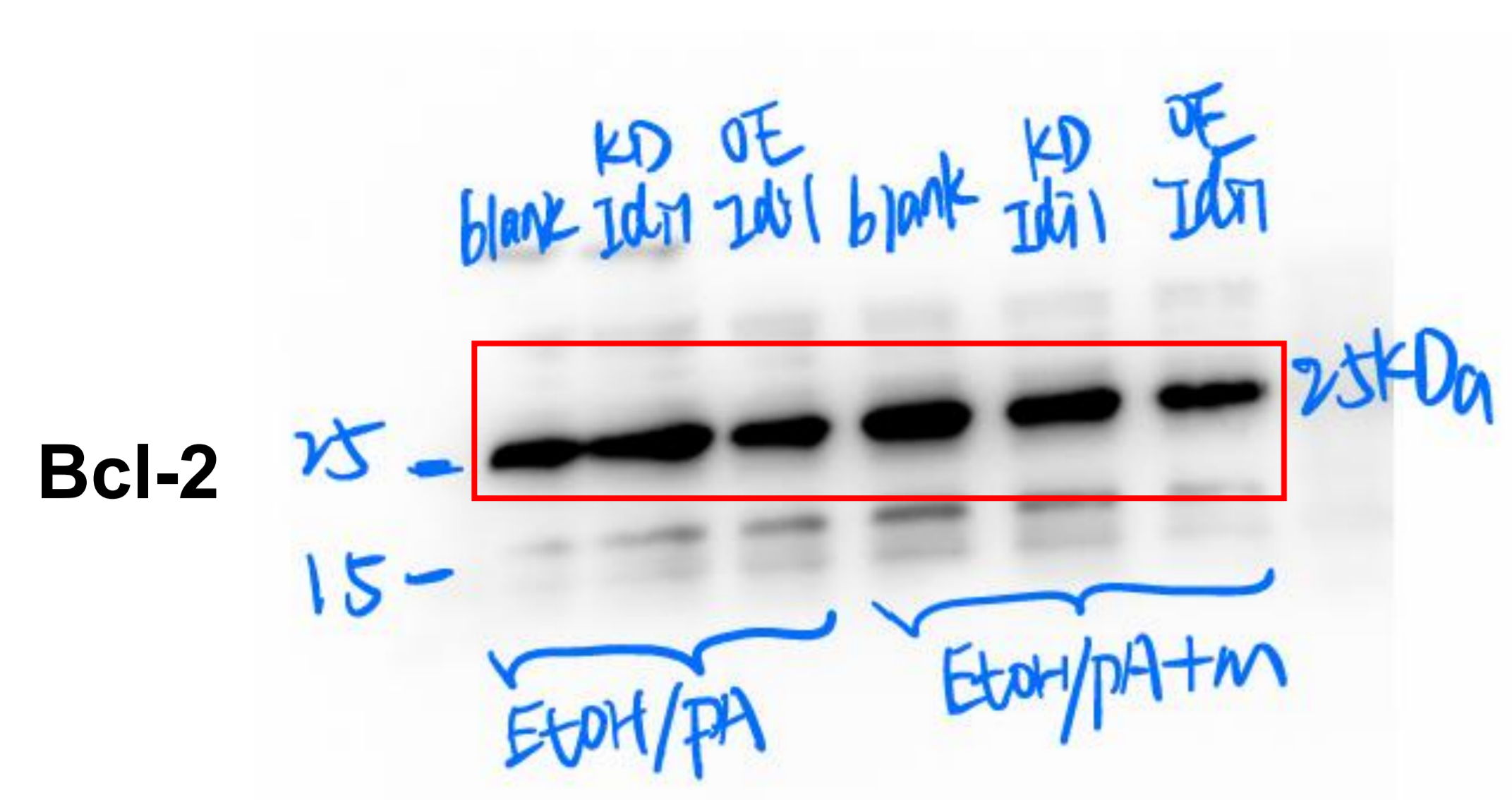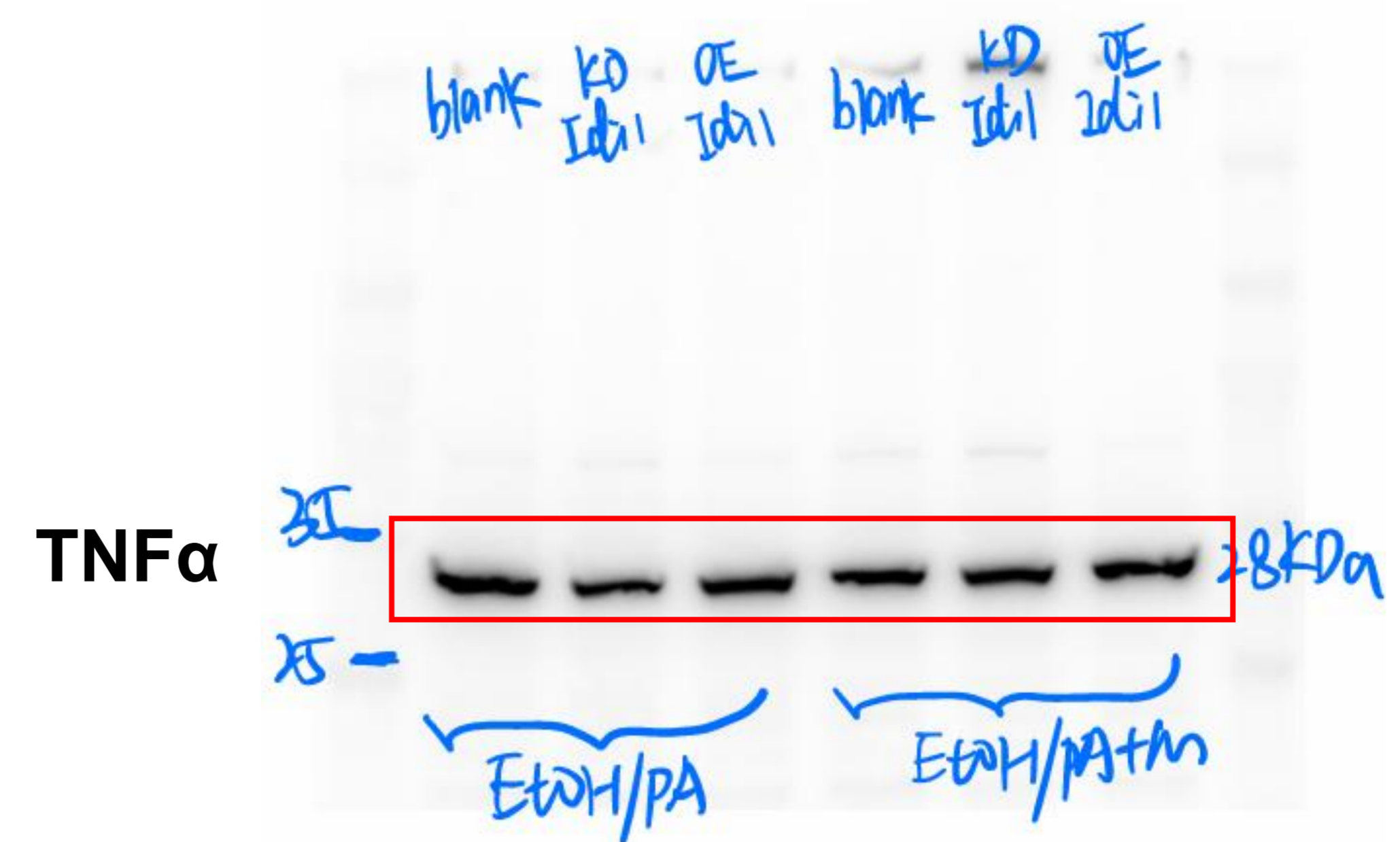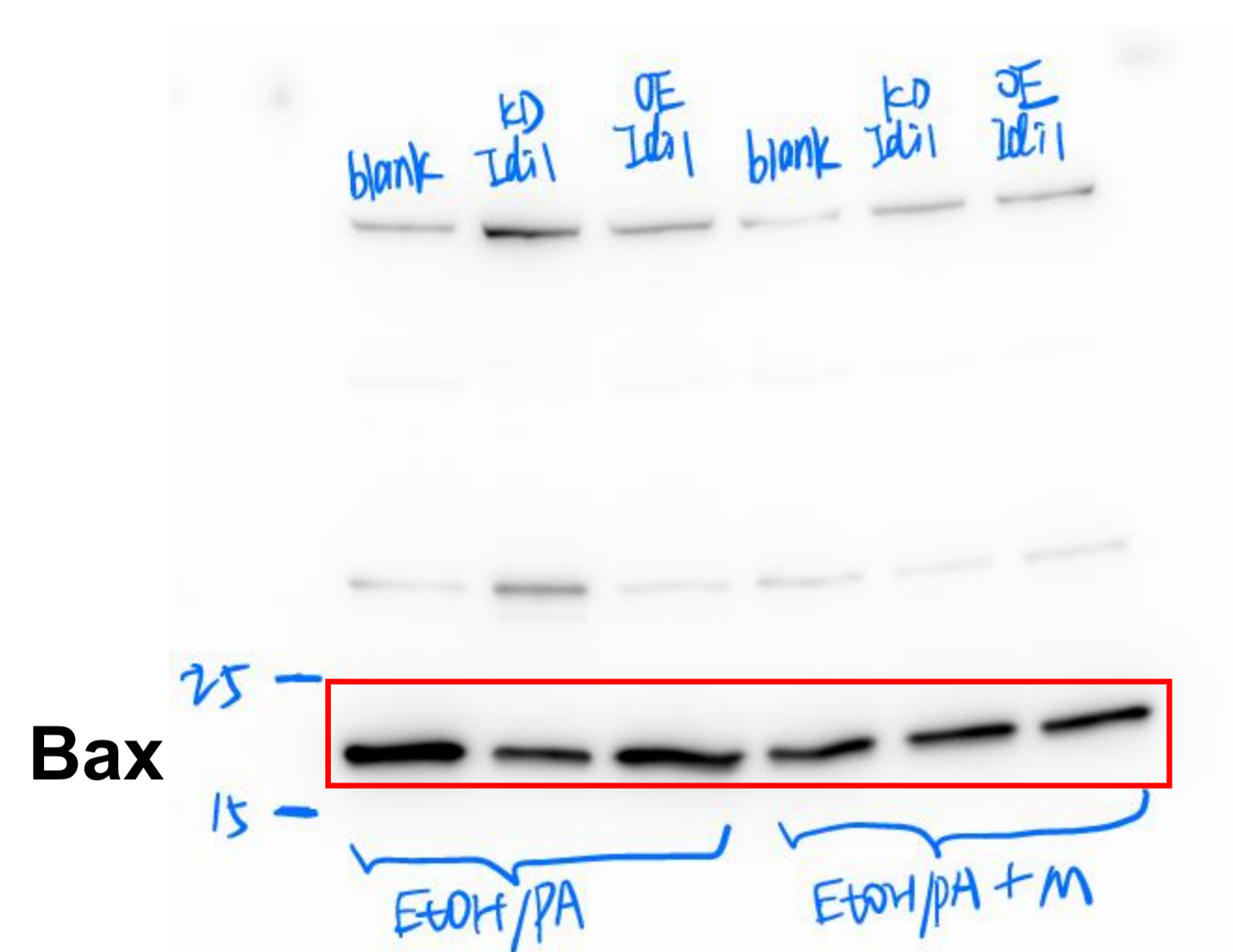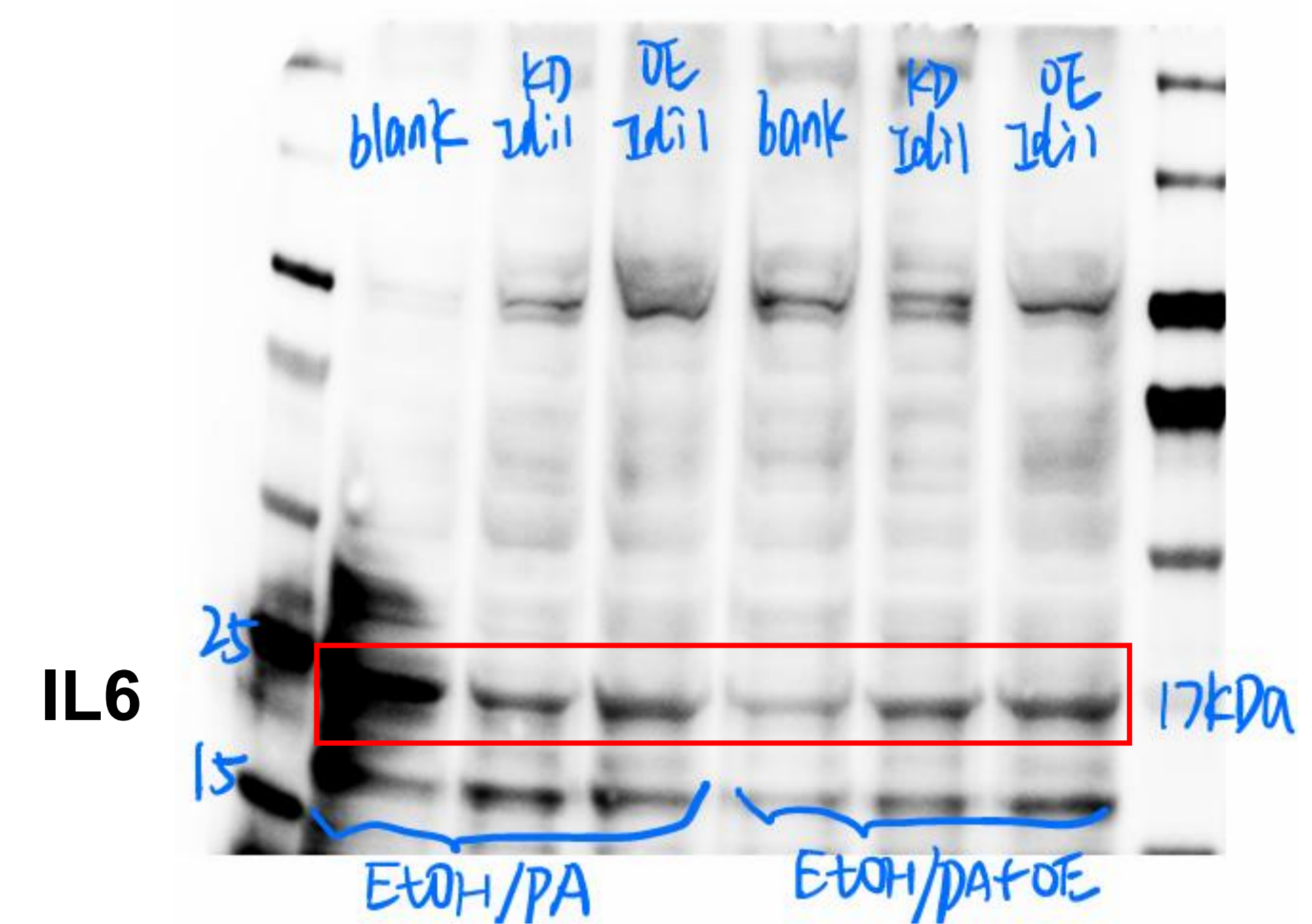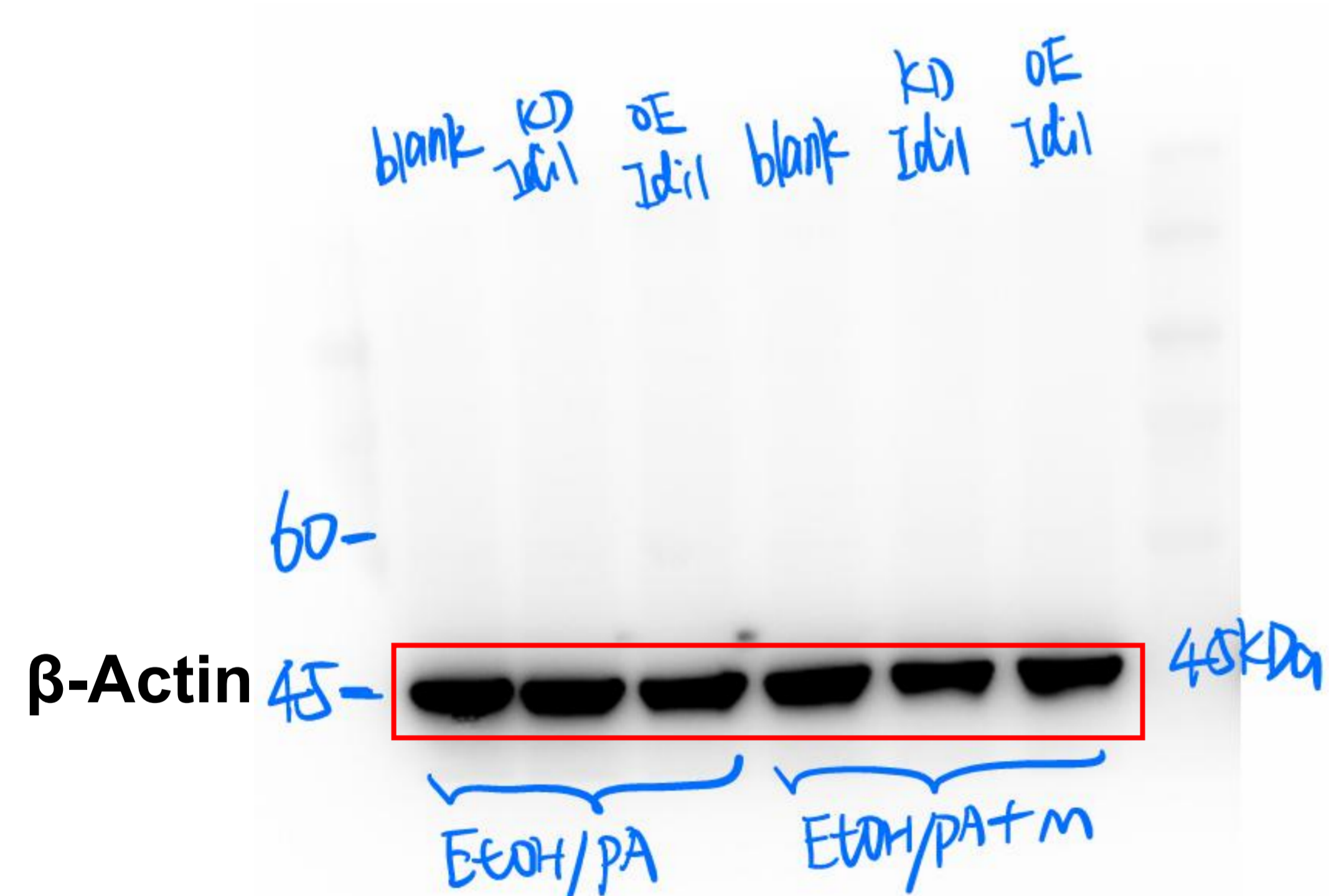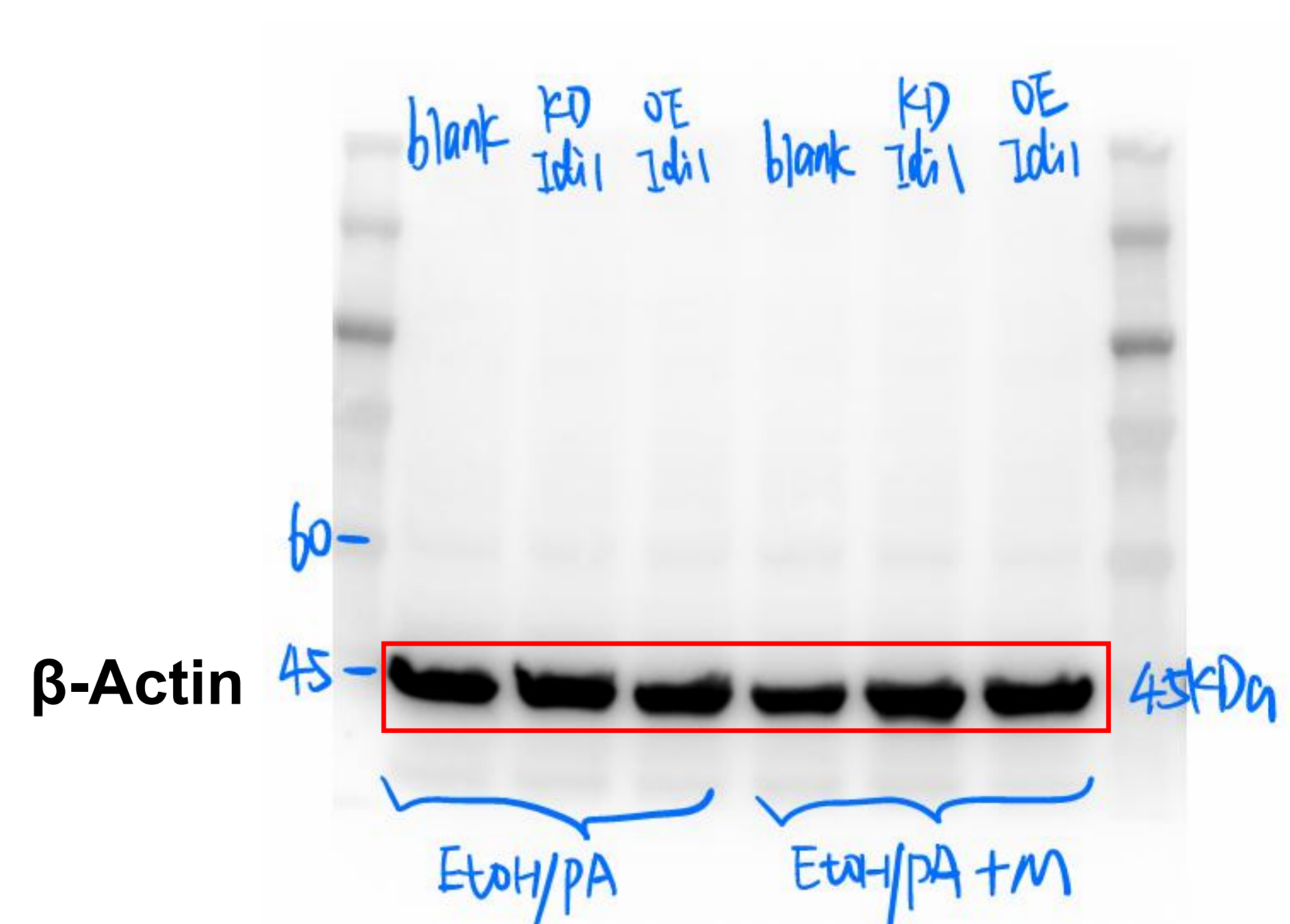

**Fig 3E, Source Data 1.** Original membranes corresponding to Figure 3E

Supplement: Figure 3—source data 1. [file elife-109174-fig3-data1.zip › Figure 3E-Source PDF.pdf]

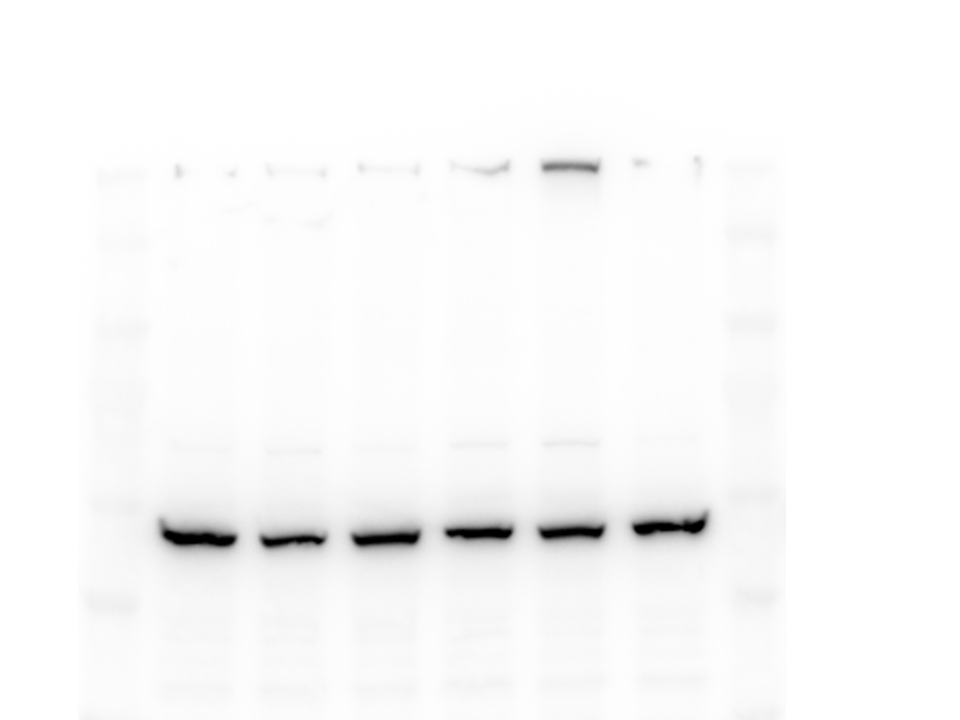

Supplement: Figure 3—source data 2. [file elife-109174-fig3-data2.zip › Fig 3E,Source data1-TNFa.tif]

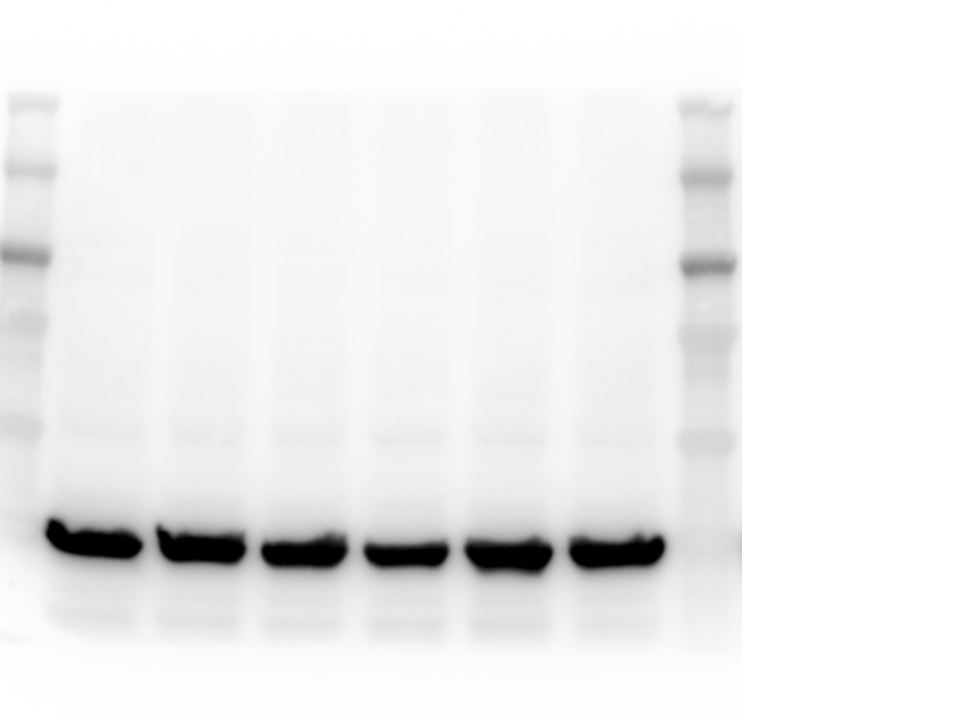

Supplement: Figure 3—source data 2. [file elife-109174-fig3-data2.zip › Fig 3E,Source data1-Actin TNFa-IL6.tif]

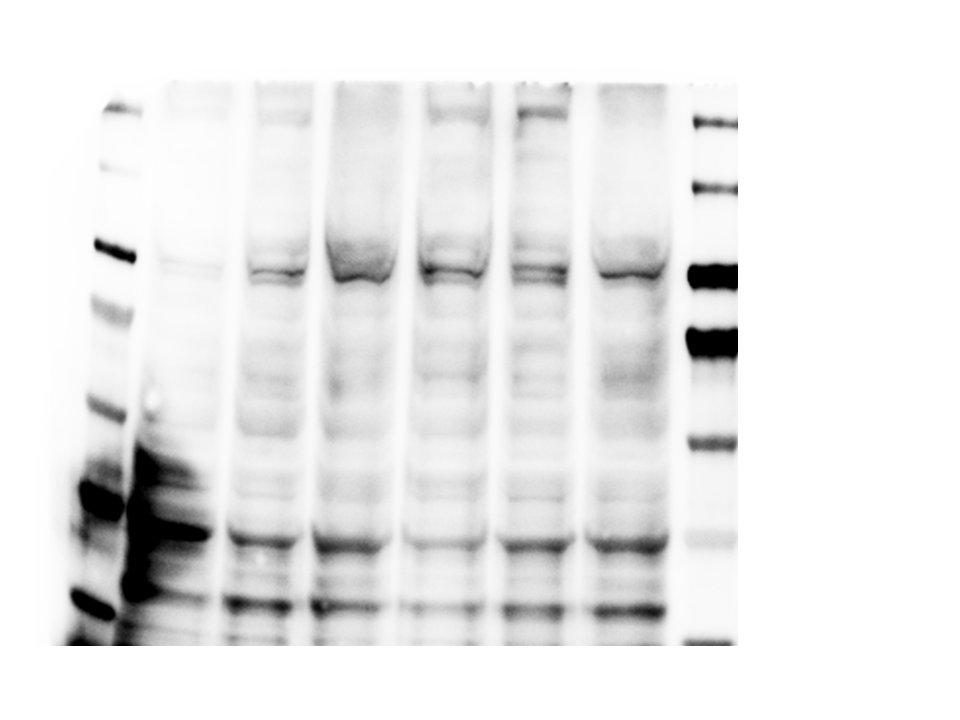

Supplement: Figure 3—source data 2. [file elife-109174-fig3-data2.zip › Fig 3E,Source data1-IL6.tif]

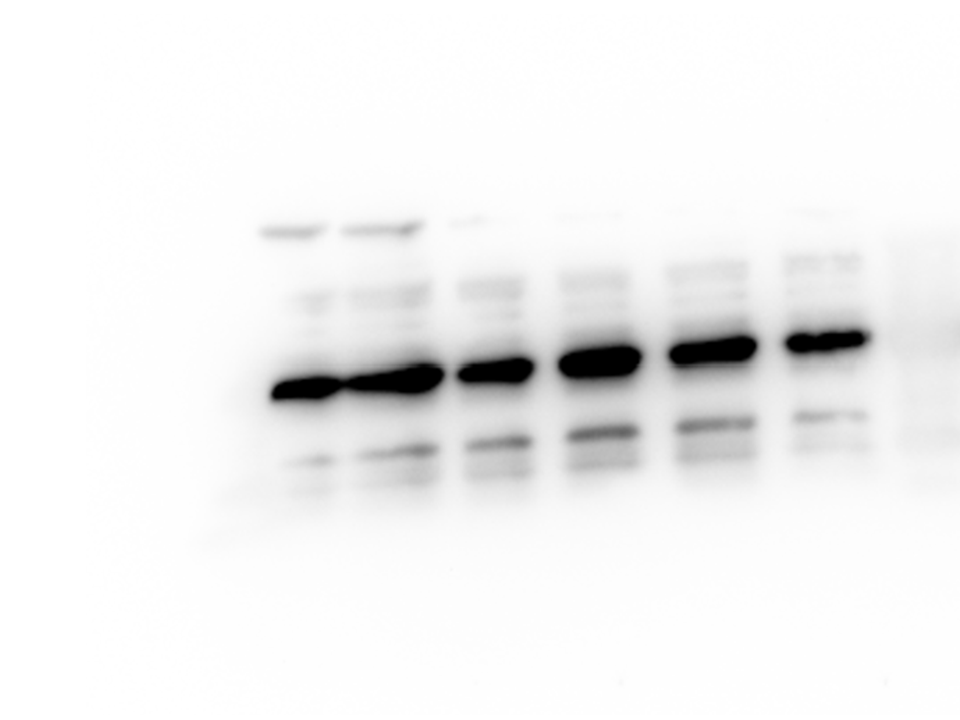

Supplement: Figure 3—source data 2. [file elife-109174-fig3-data2.zip › Fig 3E,Source data1-Bcl2.tif]

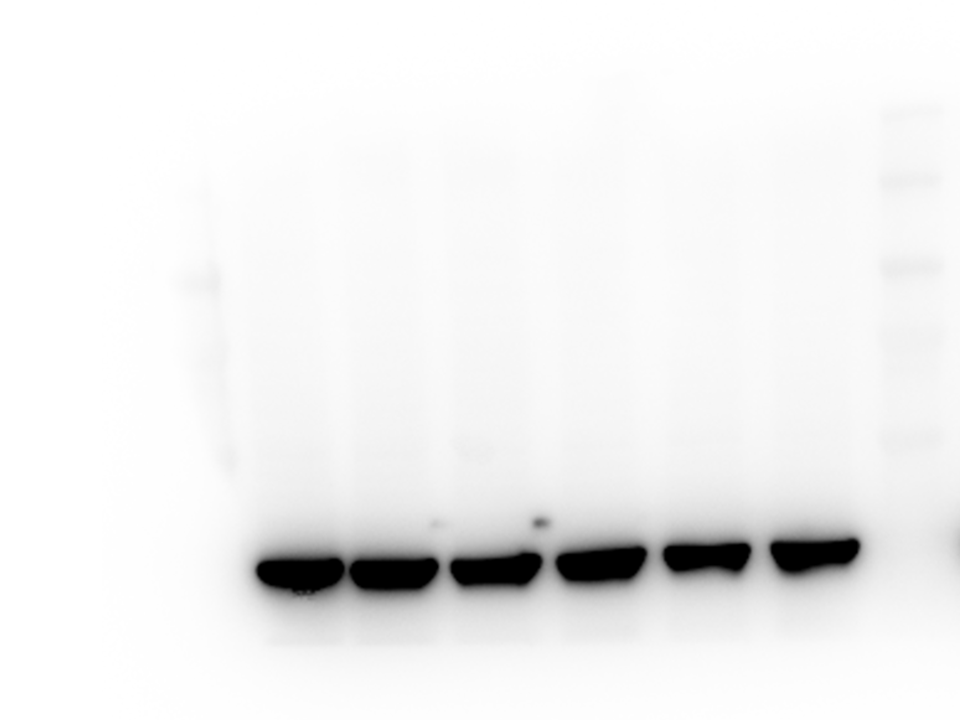

Supplement: Figure 3—source data 2. [file elife-109174-fig3-data2.zip › Fig 3E,Source data1-Actin Bax-Bcl2.tif]

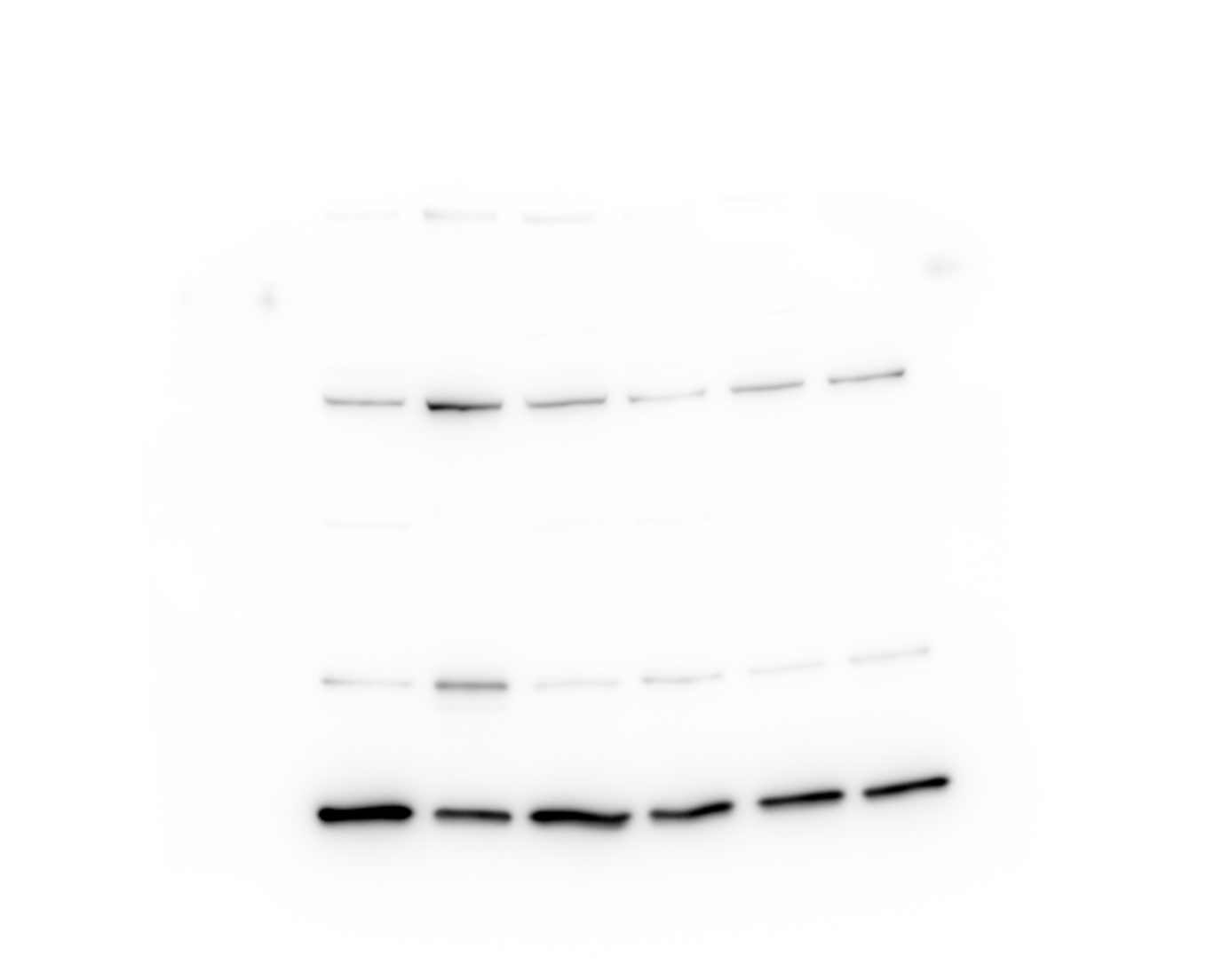

Supplement: Figure 3—source data 2. [file elife-109174-fig3-data2.zip › Fig 3E,Source data1-Bax.Tif]

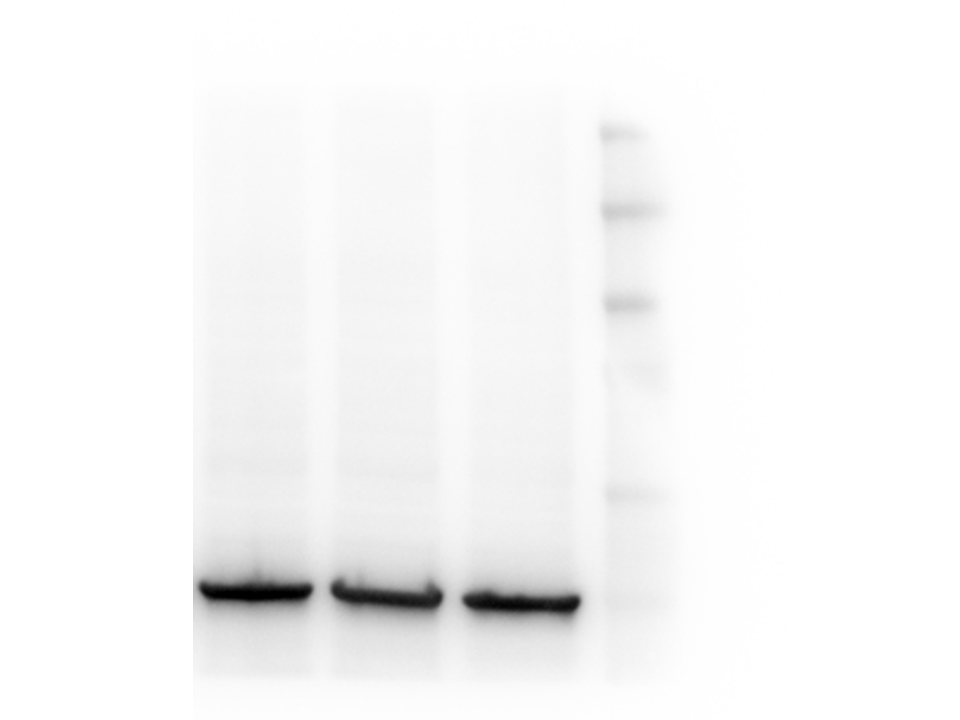

Supplement: Figure 3—figure supplement 1—source data 2. [file elife-109174-fig3-figsupp1-data2.zip › Figure 3-figure supplement 1-source data 2-Actin-Idi1-OE.tif]

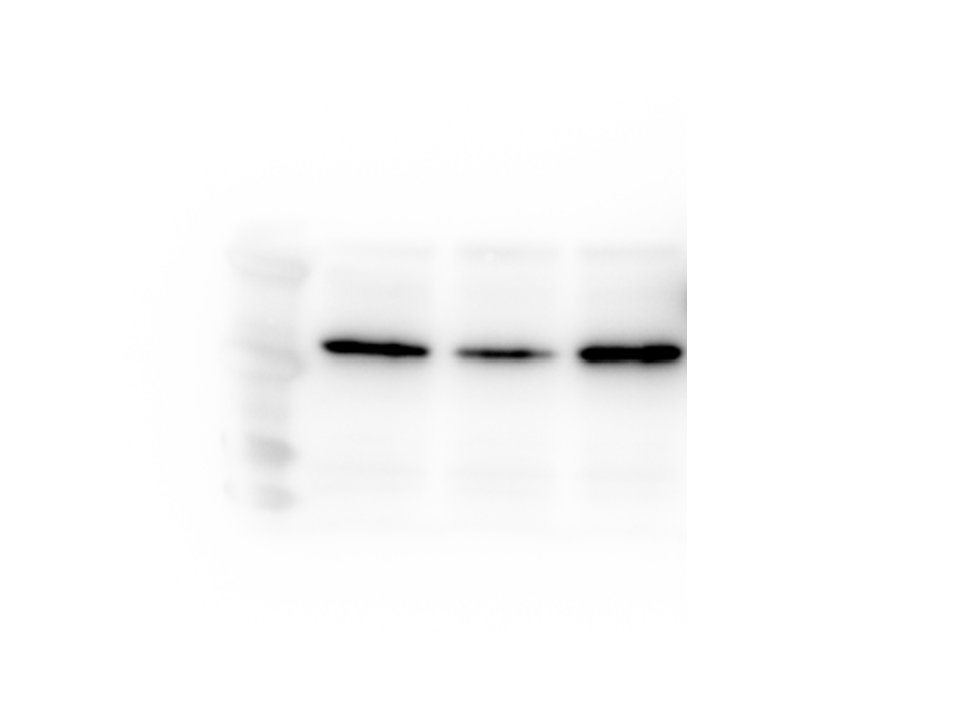

Supplement: Figure 3—figure supplement 1—source data 2. [file elife-109174-fig3-figsupp1-data2.zip › Figure 3-figure supplement 1-source data 2-Idi1-KD.tif]

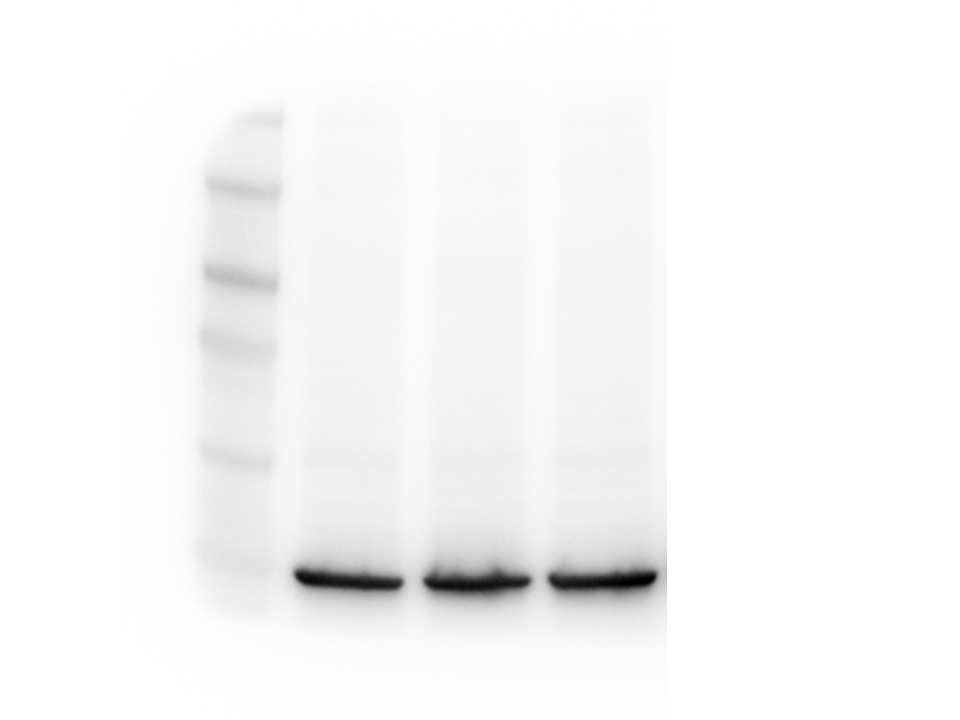

Supplement: Figure 3—figure supplement 1—source data 2. [file elife-109174-fig3-figsupp1-data2.zip › Figure 3-figure supplement 1-source data 2-Actin-Idi1-KD.tif]

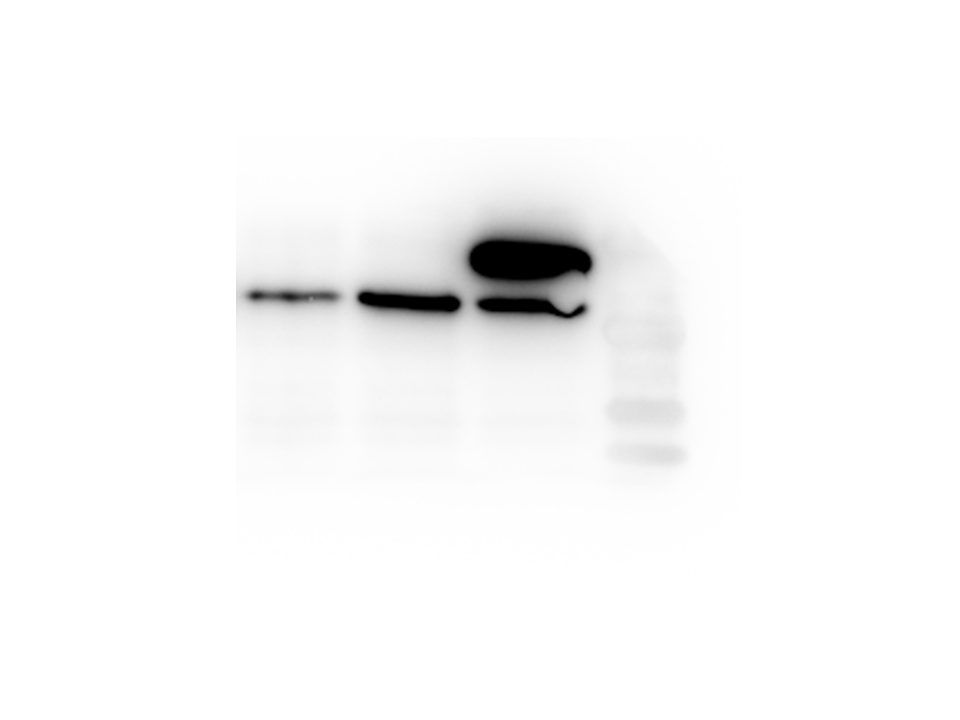

Supplement: Figure 3—figure supplement 1—source data 2. [file elife-109174-fig3-figsupp1-data2.zip › Figure 3-figure supplement 1-source data 2-Idi1-OE.tif]

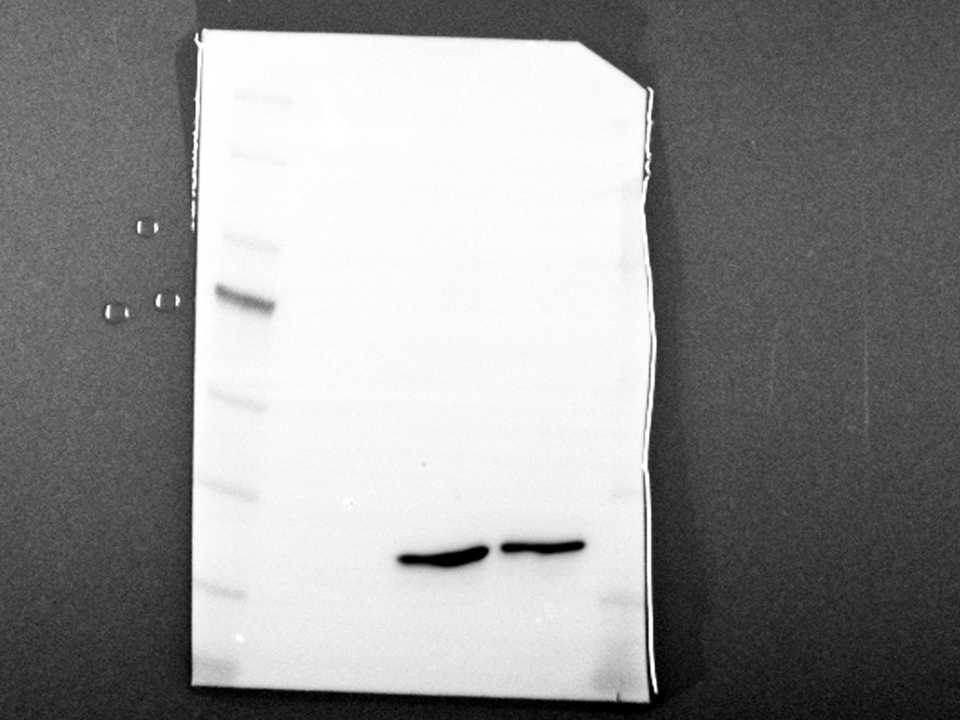

Supplement: Figure 3—figure supplement 2—source data 2. [file elife-109174-fig3-figsupp2-data2.zip › Figure 3-figure supplement 2,Source data2-E-IP Flag-WCL-Anti Flag.tif]

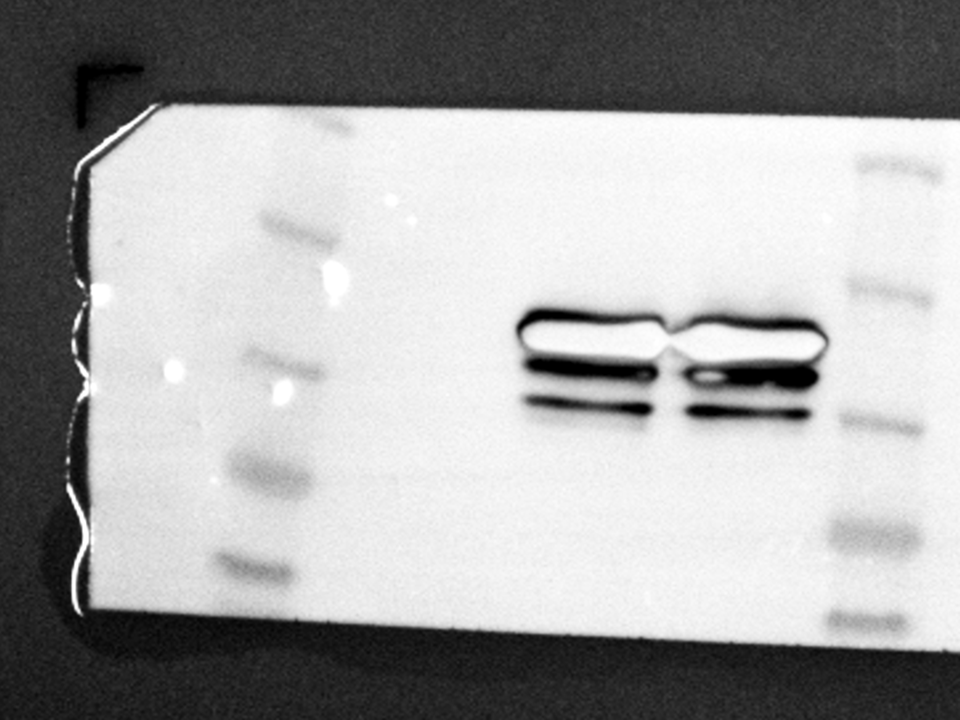

Supplement: Figure 3—figure supplement 2—source data 2. [file elife-109174-fig3-figsupp2-data2.zip › Figure 3-figure supplement 2,Source data2-E-IP Flag-Anti Flag.tif]

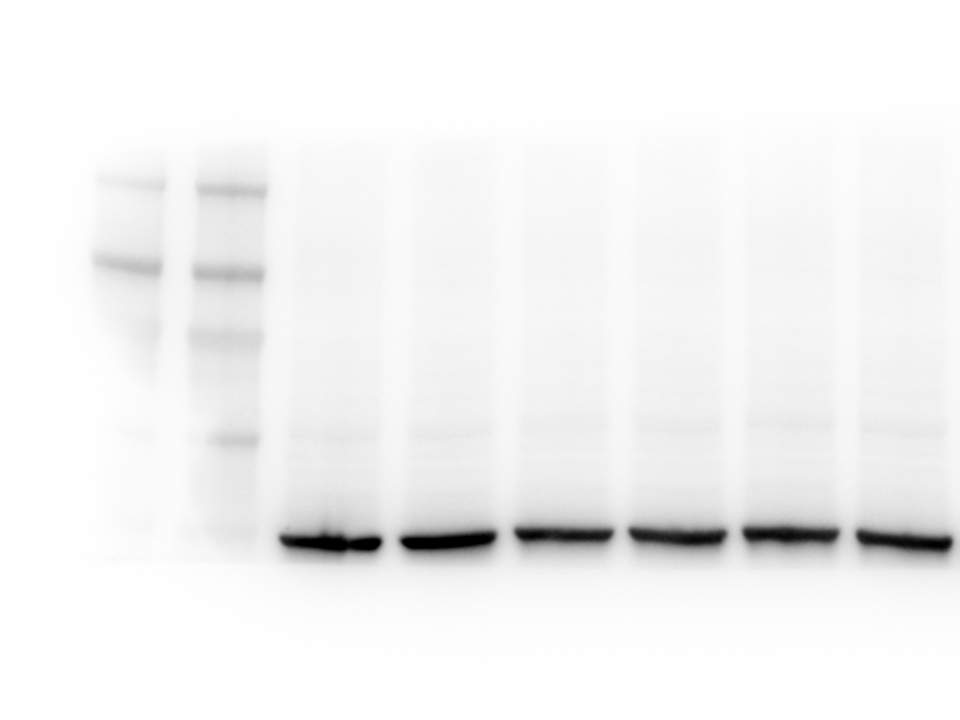

Supplement: Figure 3—figure supplement 2—source data 2. [file elife-109174-fig3-figsupp2-data2.zip › Figure 3-figure supplement 2,Source data2-D-Actin-Hsd11b1 KD.tif]

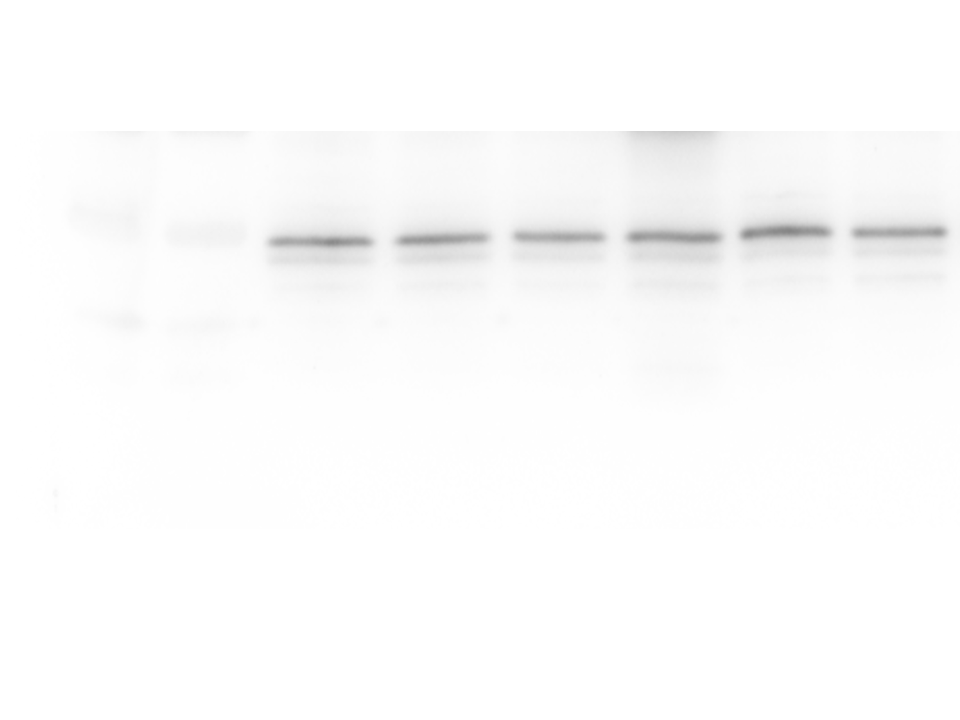

Supplement: Figure 3—figure supplement 2—source data 2. [file elife-109174-fig3-figsupp2-data2.zip › Figure 3-figure supplement 2,Source data2-D-Hsd11b1 KD.tif]

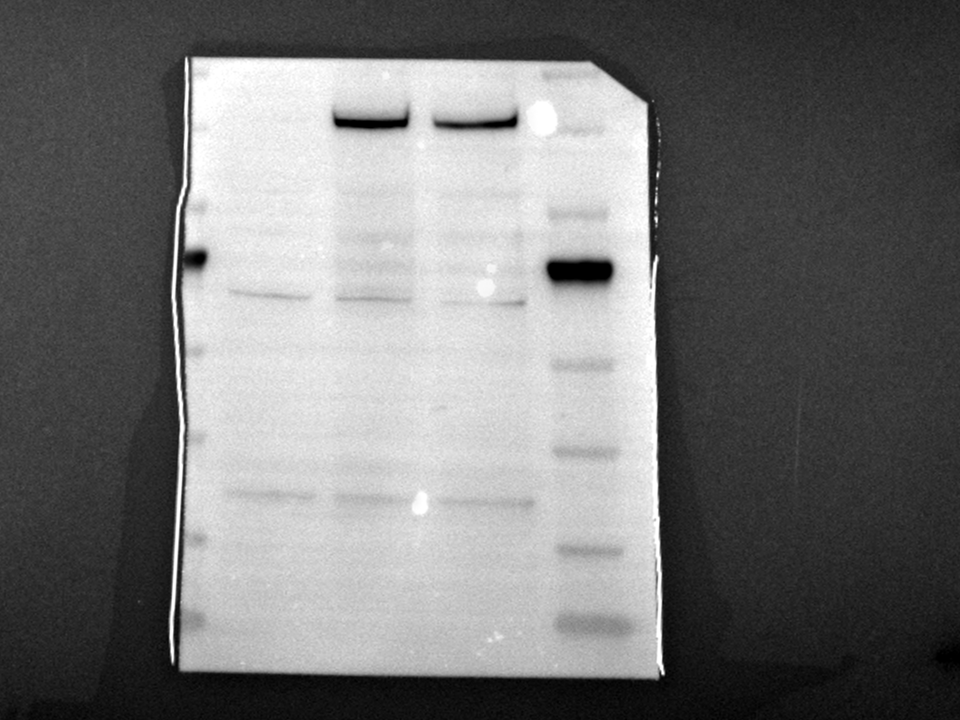

Supplement: Figure 3—figure supplement 2—source data 2. [file elife-109174-fig3-figsupp2-data2.zip › Figure 3-figure supplement 2,Source data2-E-IP HA-WCL-Anti HA.tif]

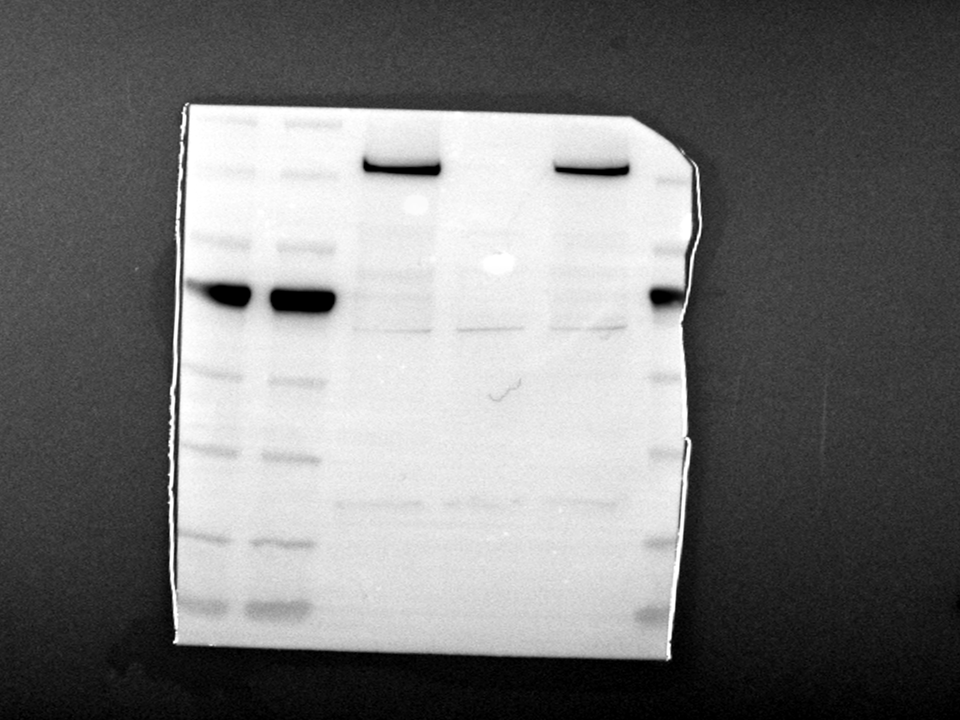

Supplement: Figure 3—figure supplement 2—source data 2. [file elife-109174-fig3-figsupp2-data2.zip › Figure 3-figure supplement 2,Source data2-E-IP Flag-WCL-Anti HA.tif]

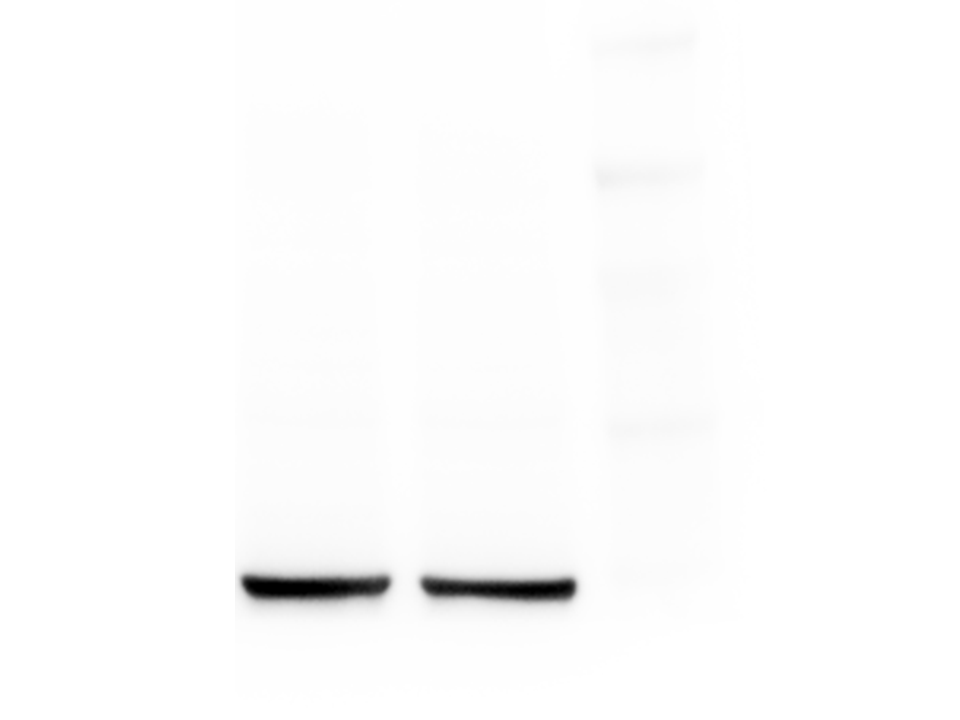

Supplement: Figure 3—figure supplement 2—source data 2. [file elife-109174-fig3-figsupp2-data2.zip › Figure 3-figure supplement 2,Source data2-D-Actin-Hsd11b1 OE.tif]

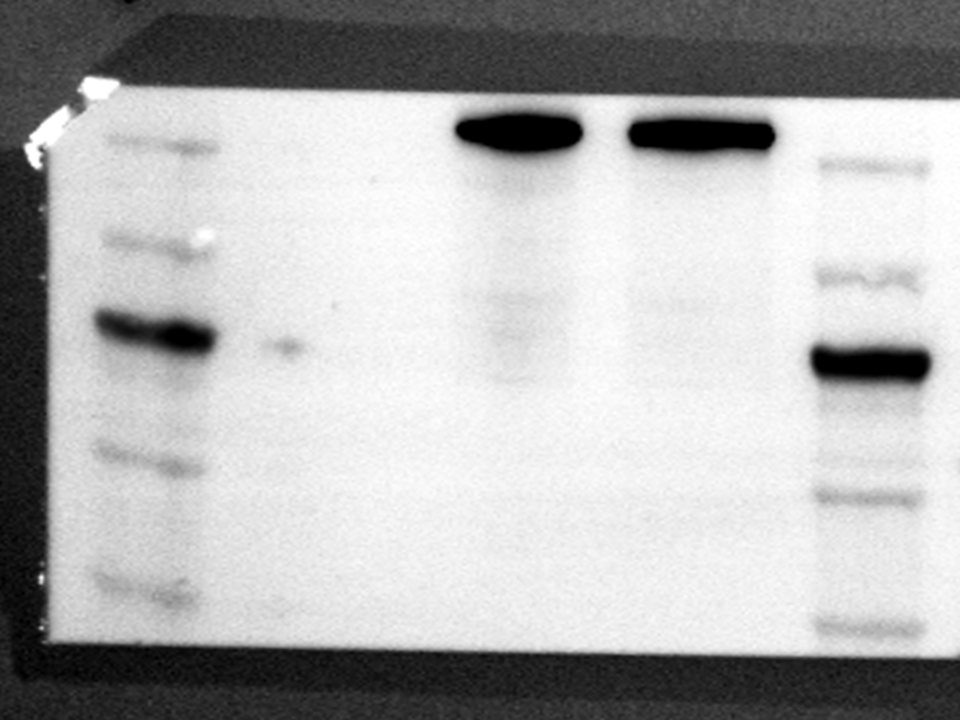

Supplement: Figure 3—figure supplement 2—source data 2. [file elife-109174-fig3-figsupp2-data2.zip › Figure 3-figure supplement 2,Source data2-E-IP HA-Anti HA.tif]

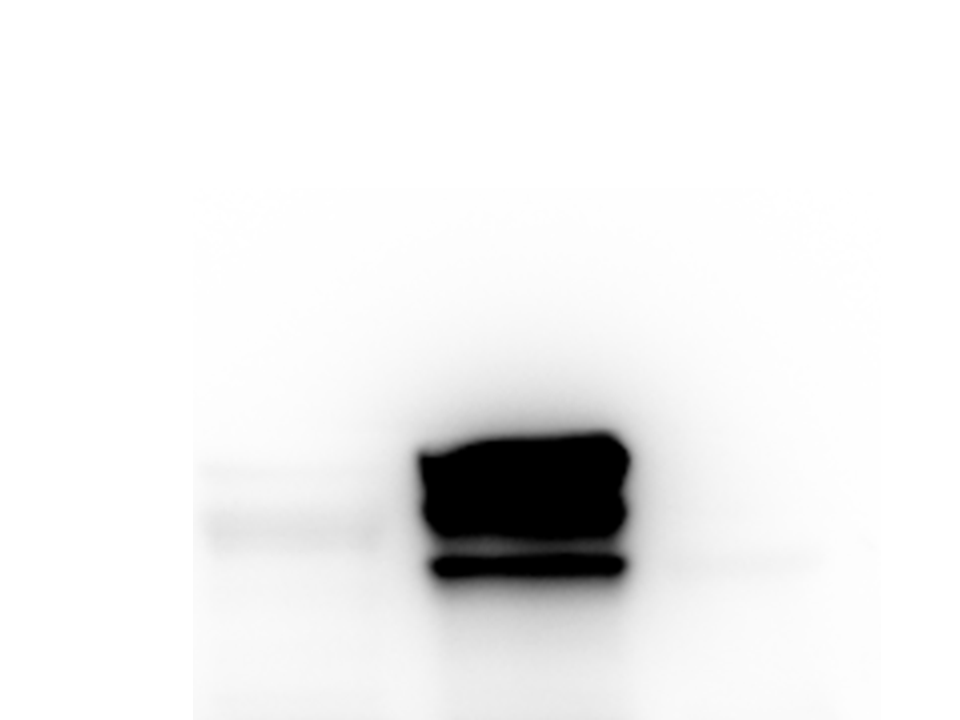

Supplement: Figure 3—figure supplement 2—source data 2. [file elife-109174-fig3-figsupp2-data2.zip › Figure 3-figure supplement 2,Source data2-D-Hsd11b1 OE.tif]

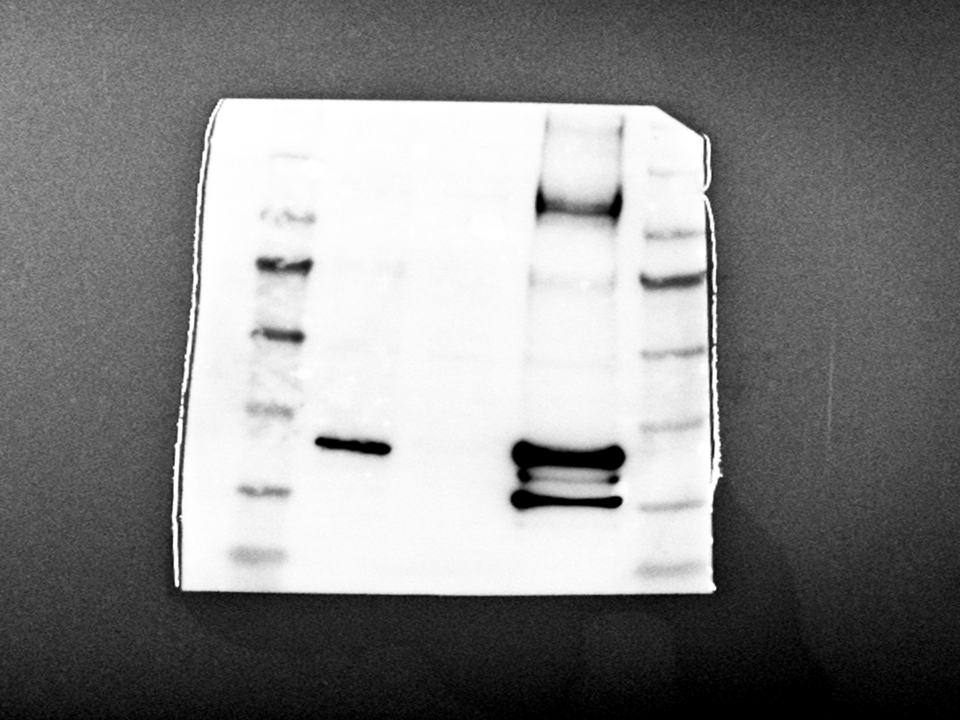

Supplement: Figure 3—figure supplement 2—source data 2. [file elife-109174-fig3-figsupp2-data2.zip › Figure 3-figure supplement 2,Source data2-E-IP HA-Anti Flag.tif]

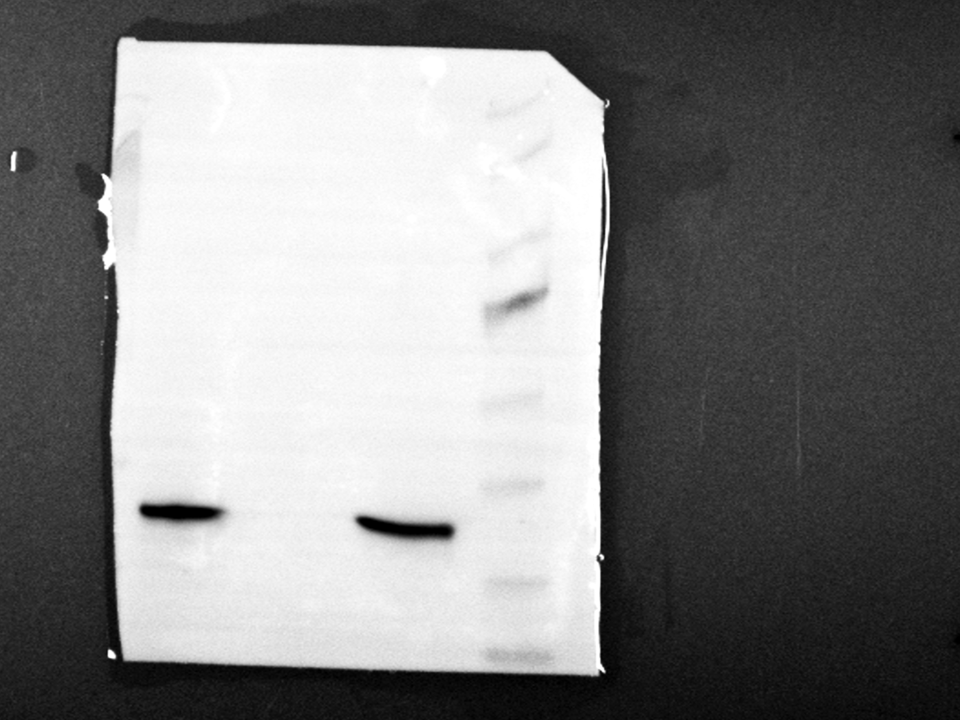

Supplement: Figure 3—figure supplement 2—source data 2. [file elife-109174-fig3-figsupp2-data2.zip › Figure 3-figure supplement 2,Source data2-E-IP HA-WCL-Anti Flag.tif]

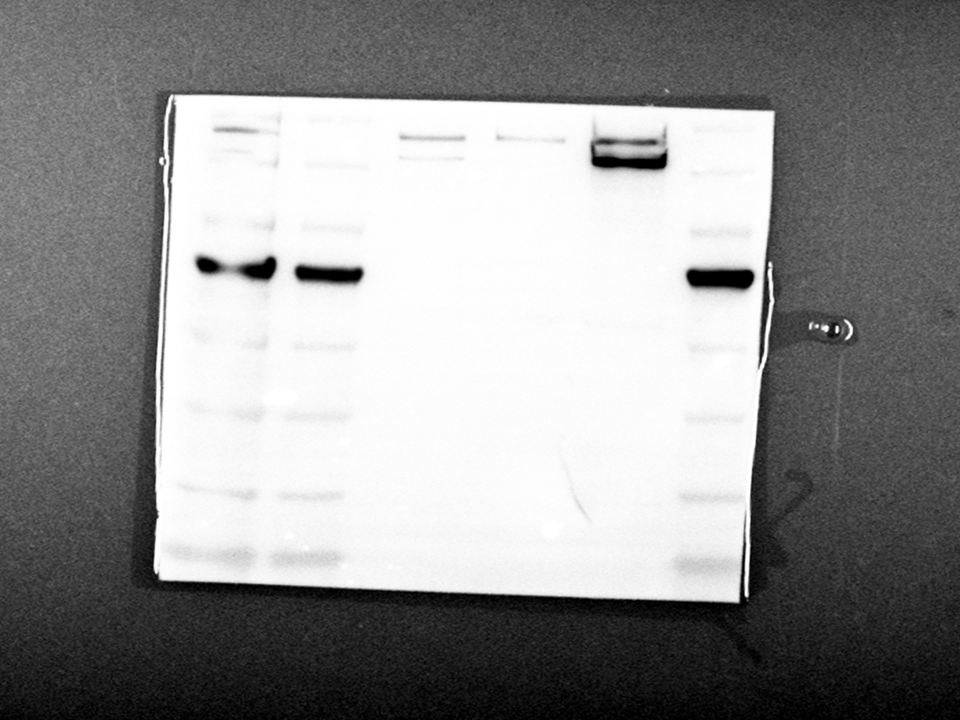

Supplement: Figure 3—figure supplement 2—source data 2. [file elife-109174-fig3-figsupp2-data2.zip › Figure 3-figure supplement 2,Source data2-E-IP Flag-Anti HA.tif]

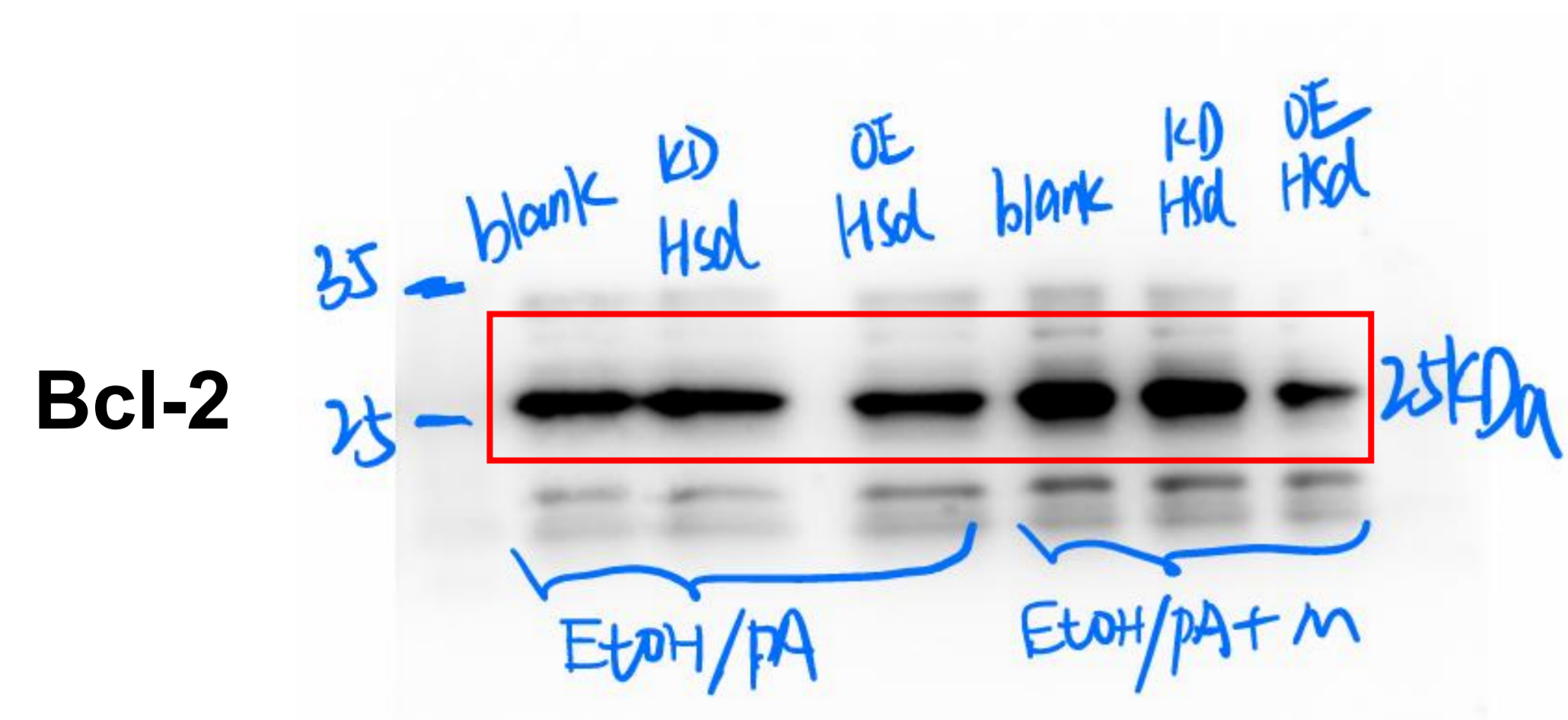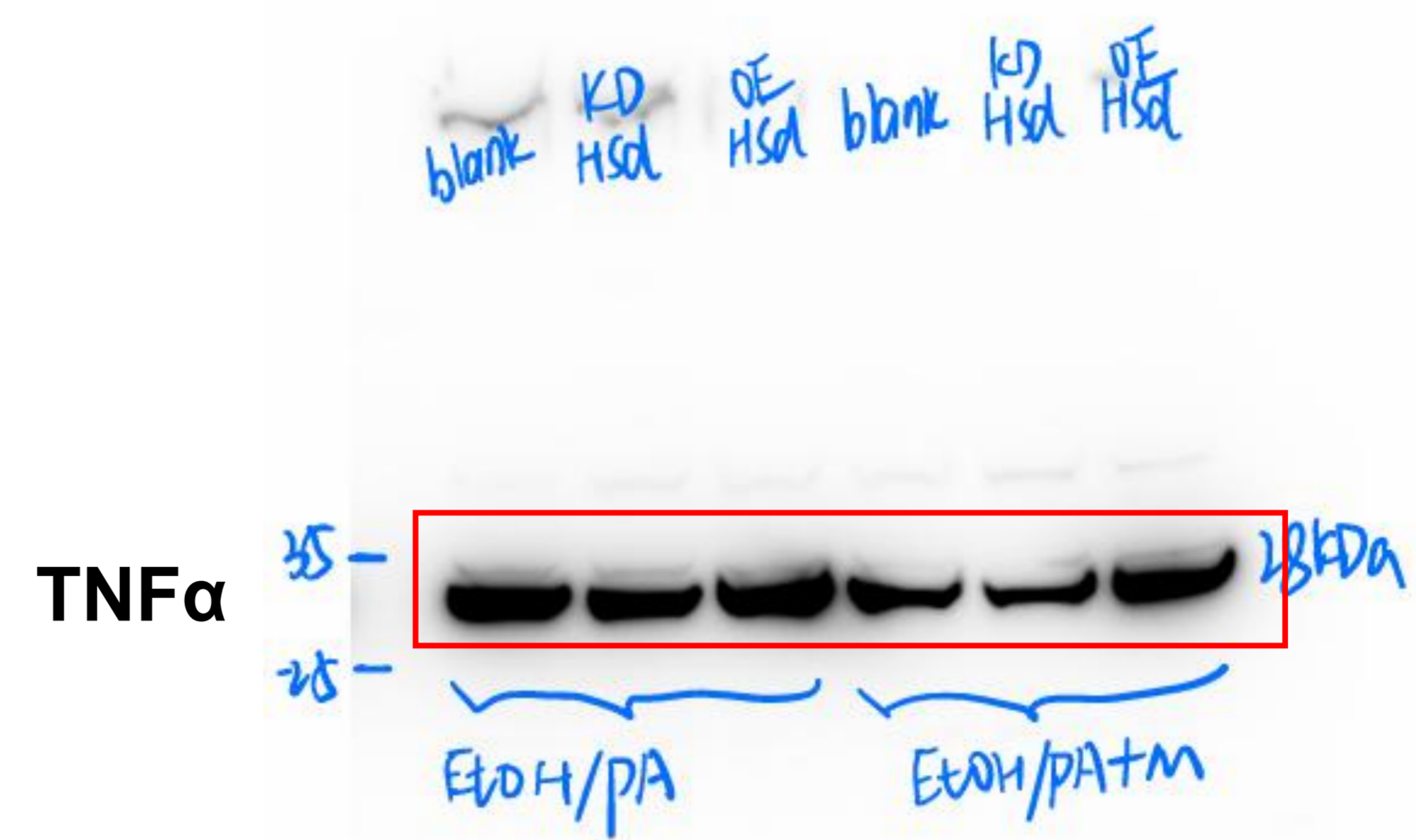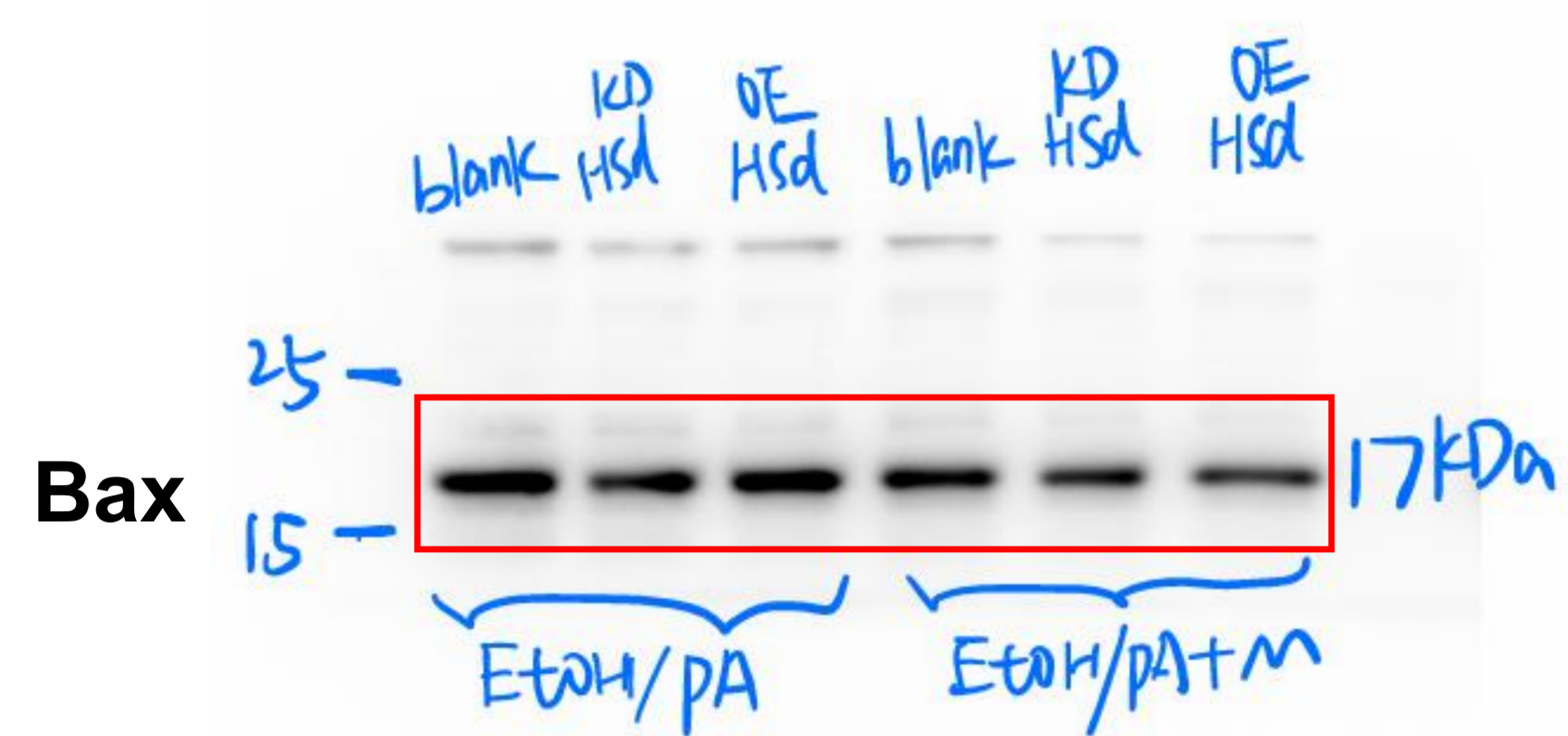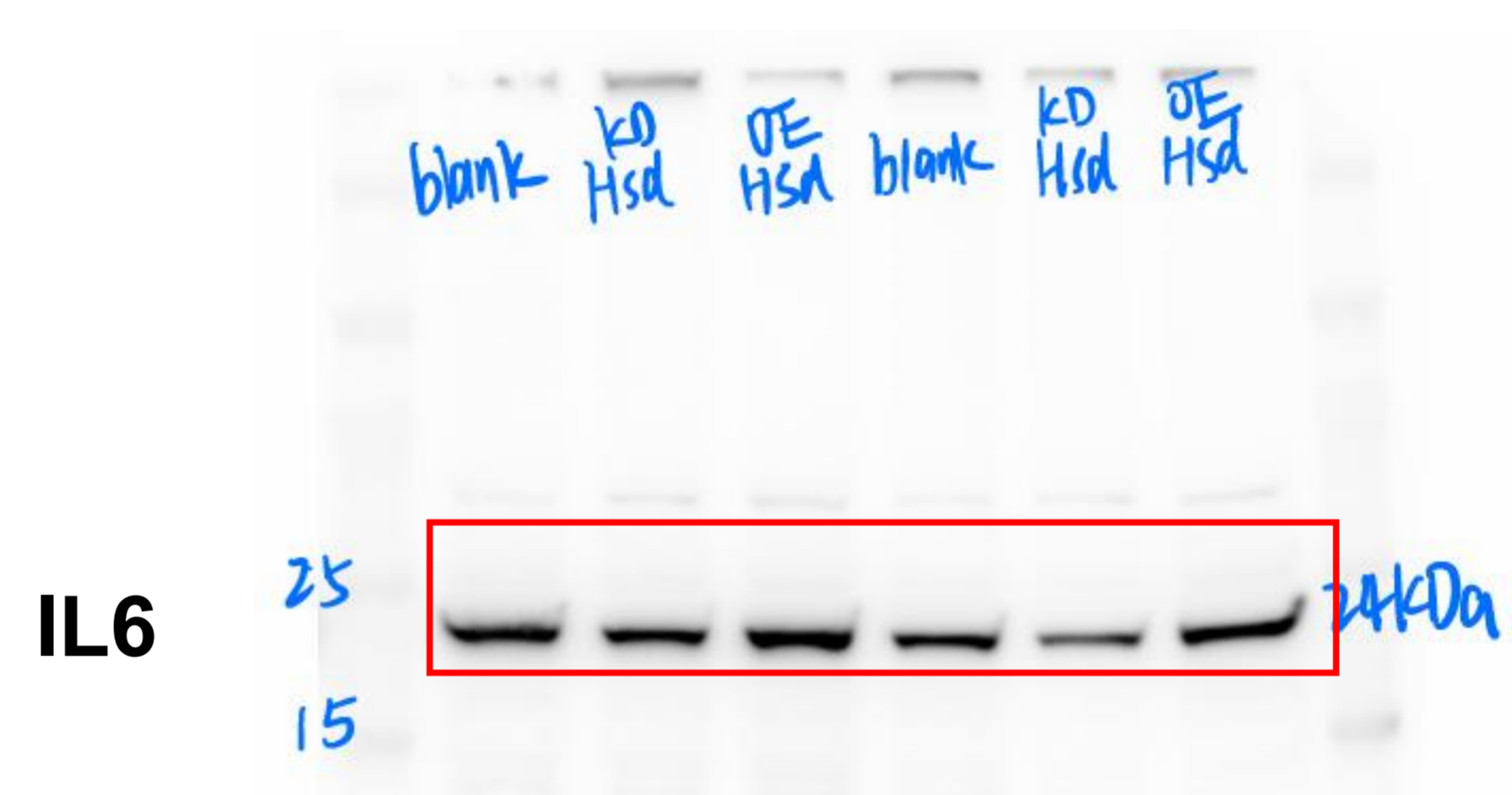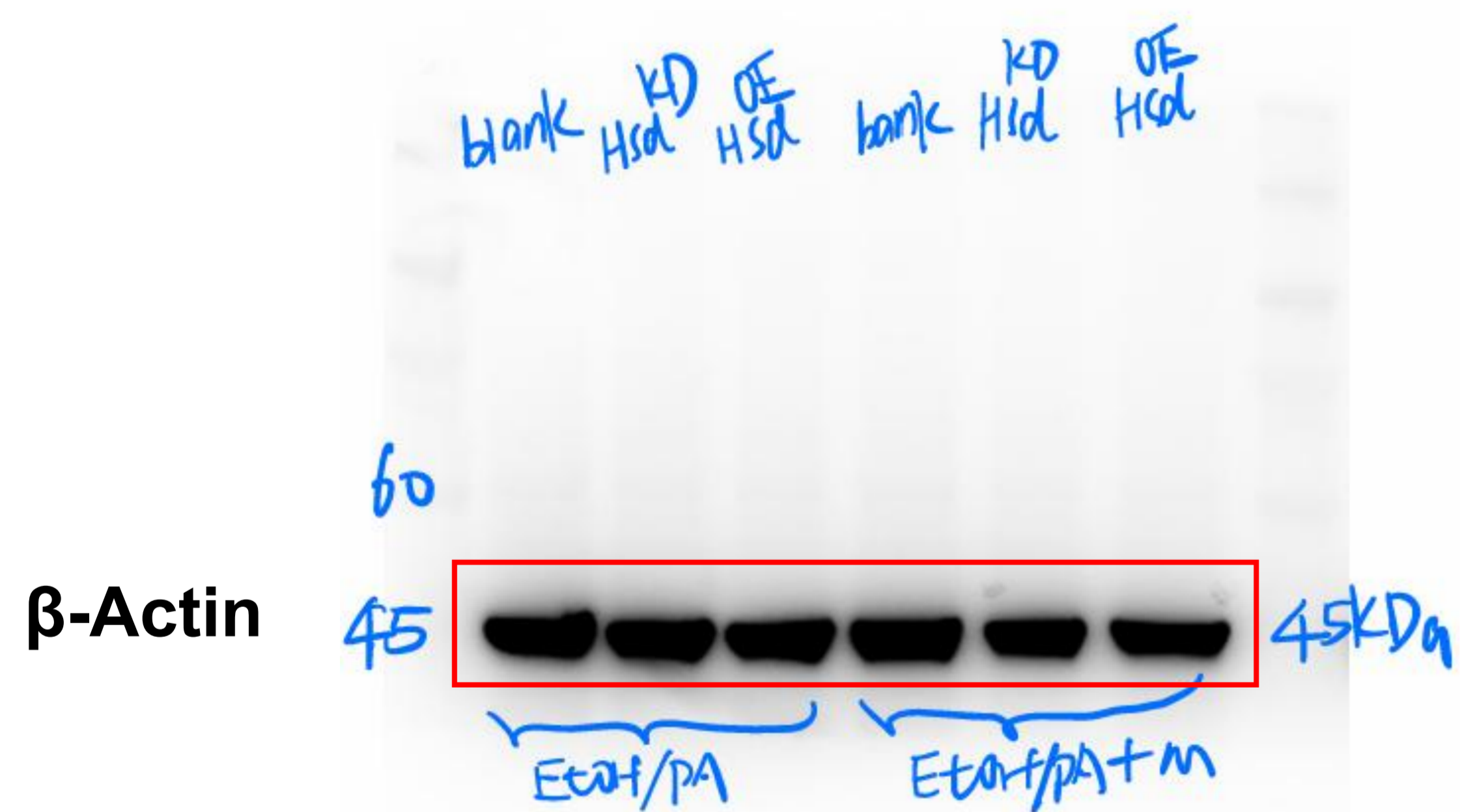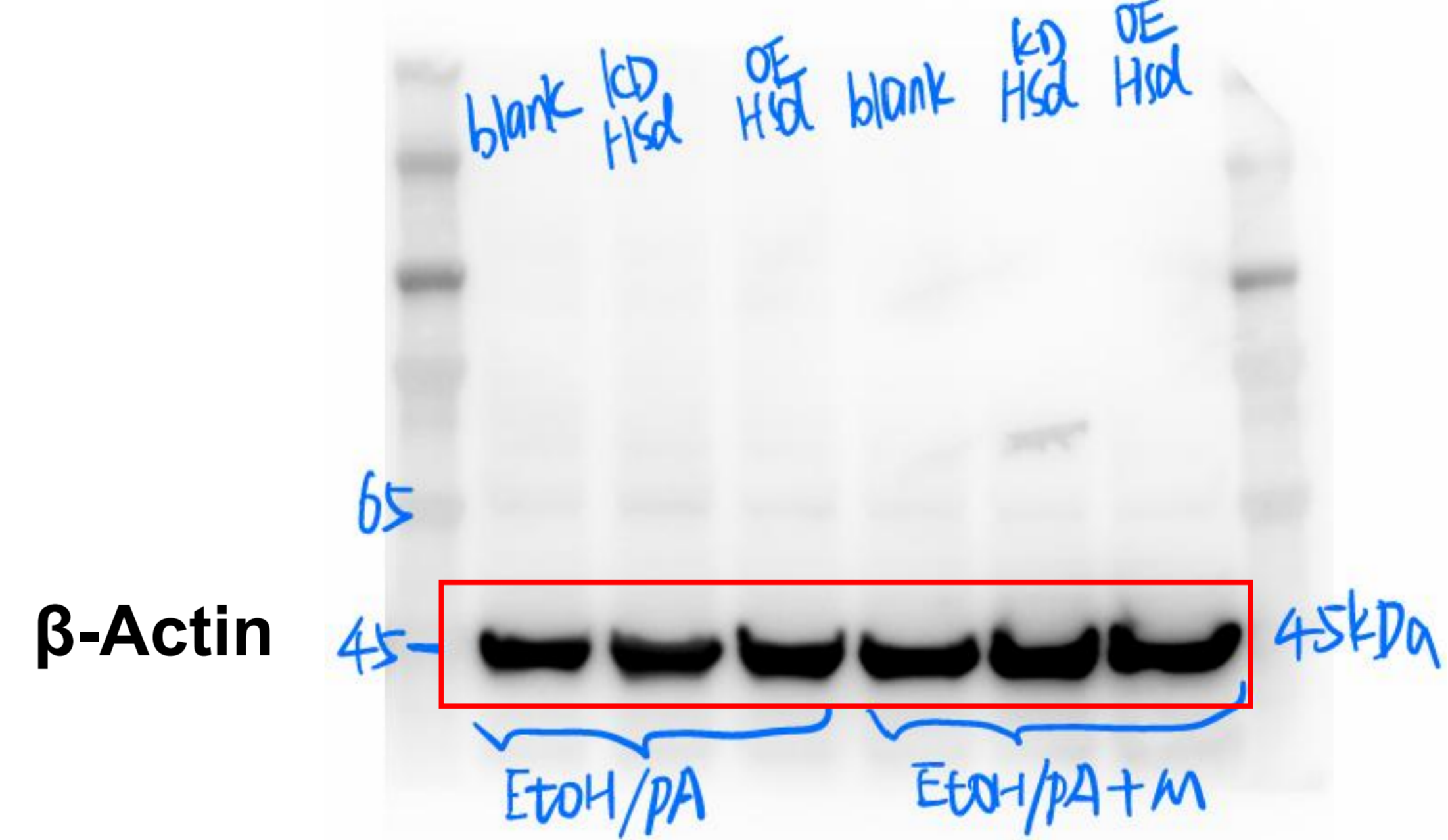

**Fig 5E, Source Data 1.** Original membranes corresponding to Figure 5E

Supplement: Figure 5—source data 1. [file elife-109174-fig5-data1.zip › Figure 5E-Source PDF.pdf]

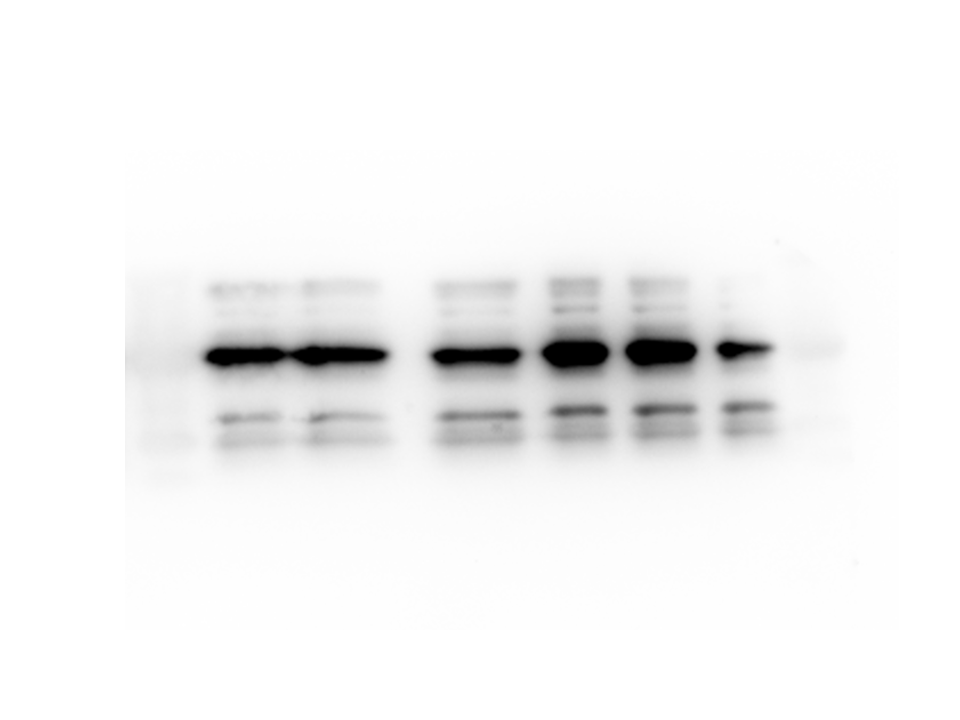

Supplement: Figure 5—source data 2. [file elife-109174-fig5-data2.zip › Fig 5E,Source data2-Bcl2.tif]

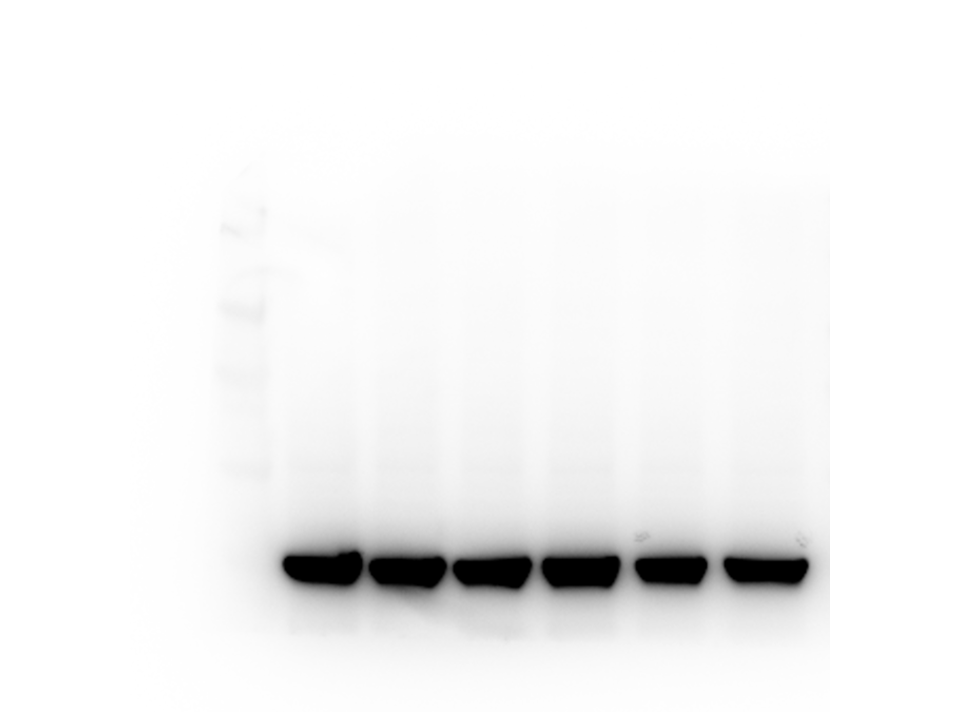

Supplement: Figure 5—source data 2. [file elife-109174-fig5-data2.zip › Fig 5E,Source data2-Actin-Bcl2-Bax.tif]

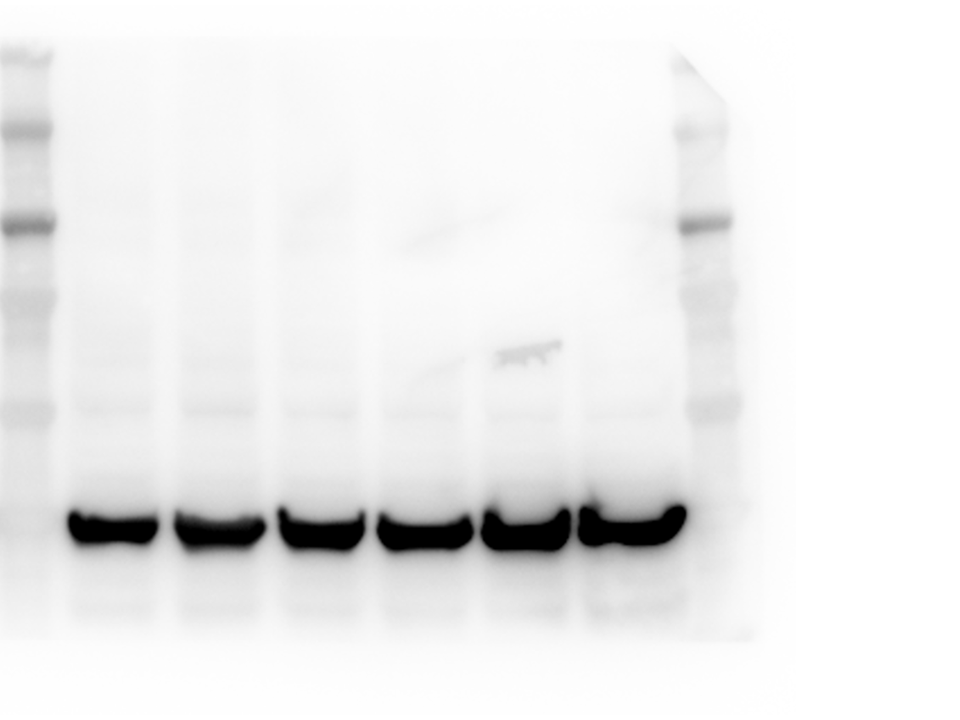

Supplement: Figure 5—source data 2. [file elife-109174-fig5-data2.zip › Fig 5E,Source data2-Actin-TNFa-IL6.tif]

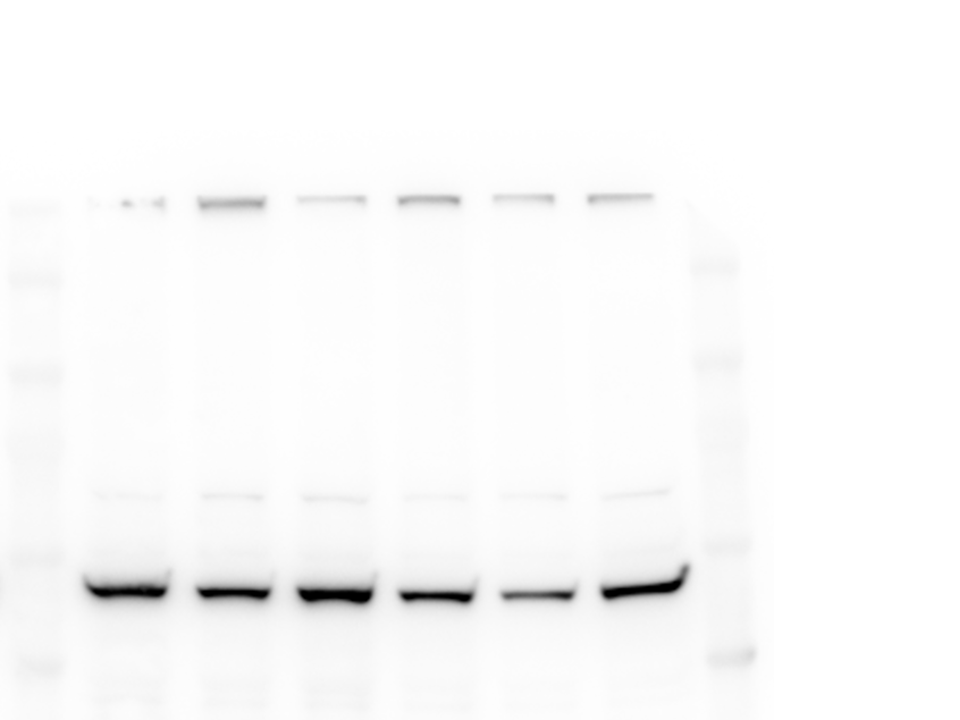

Supplement: Figure 5—source data 2. [file elife-109174-fig5-data2.zip › Fig 5E,Source data2-IL6.tif]

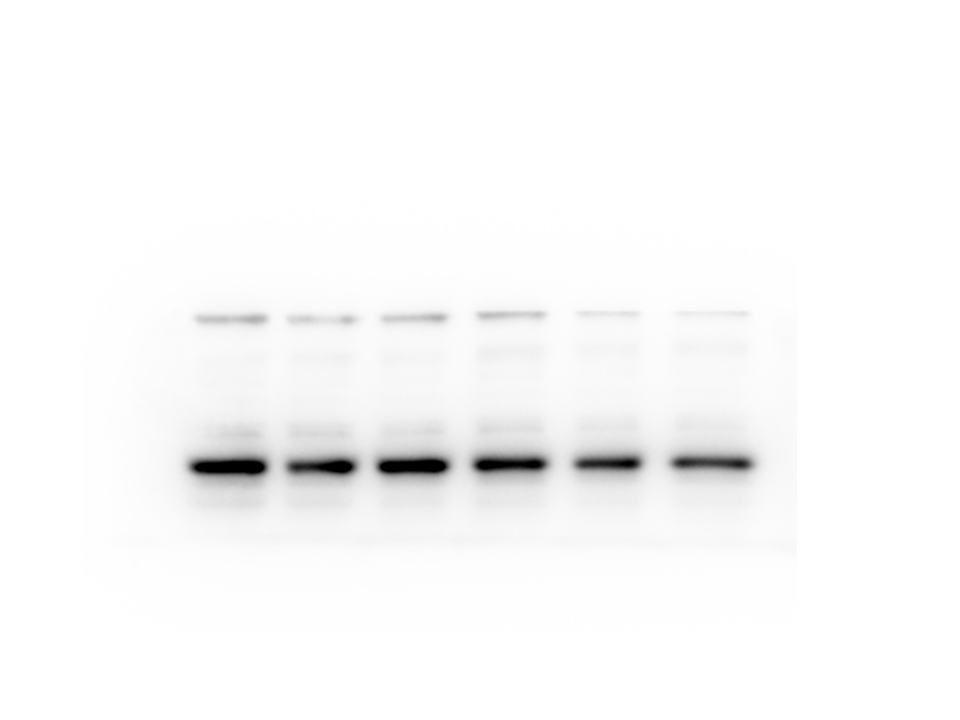

Supplement: Figure 5—source data 2. [file elife-109174-fig5-data2.zip › Fig 5E,Source data2-Bax.tif]

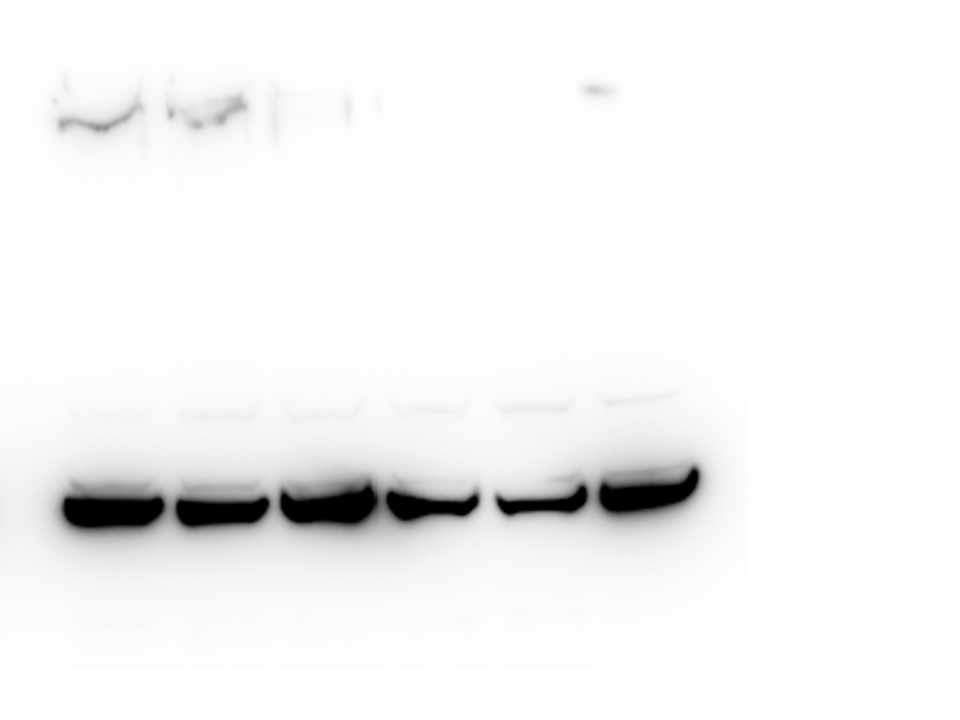

Supplement: Figure 5—source data 2. [file elife-109174-fig5-data2.zip › Fig 5E,Source data2-TNFa.tif]

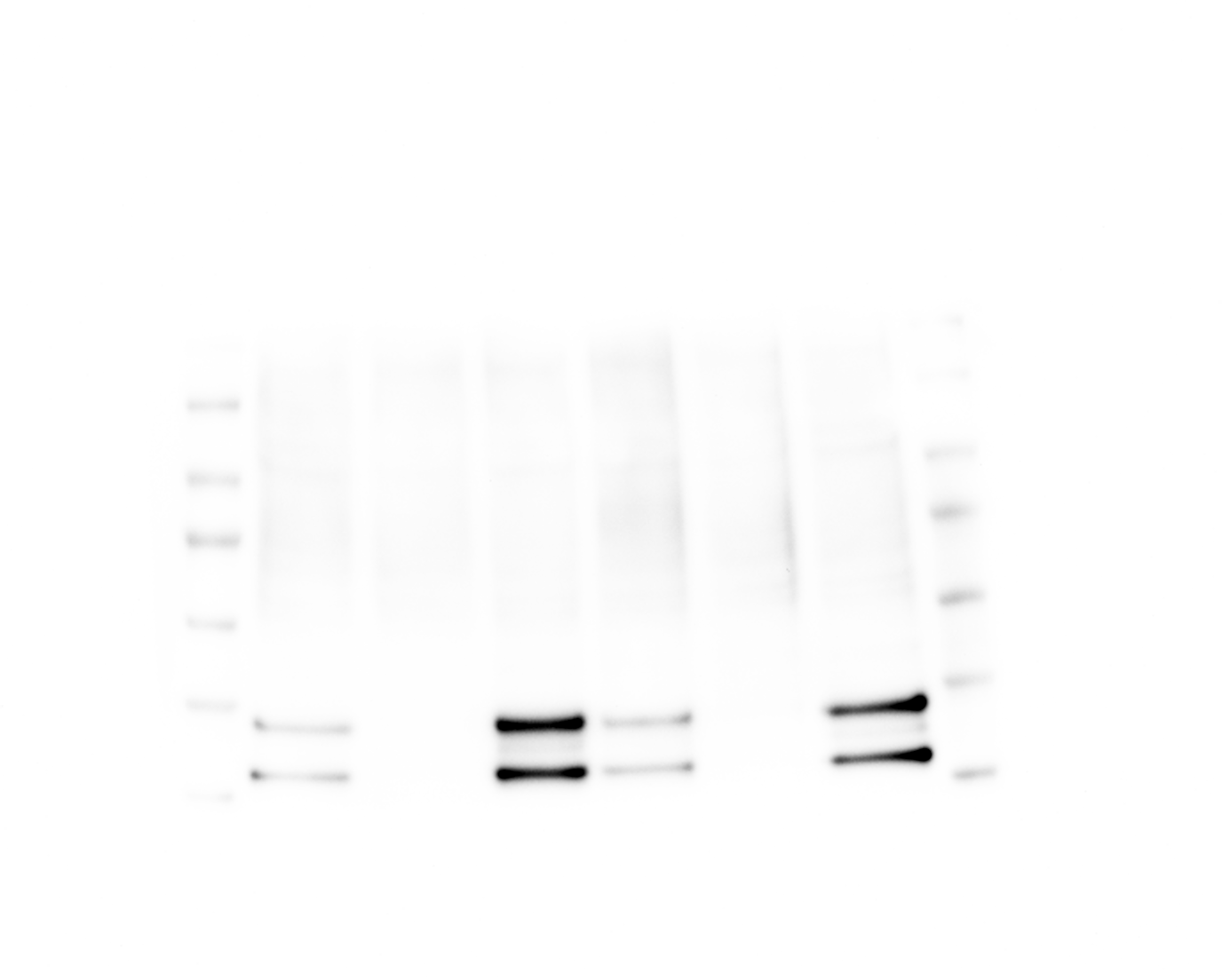

Supplement: Figure 6—source data 2. [file elife-109174-fig6-data2.zip › Fig 6,Source data2-F-IP Flag anti Myc.tif]

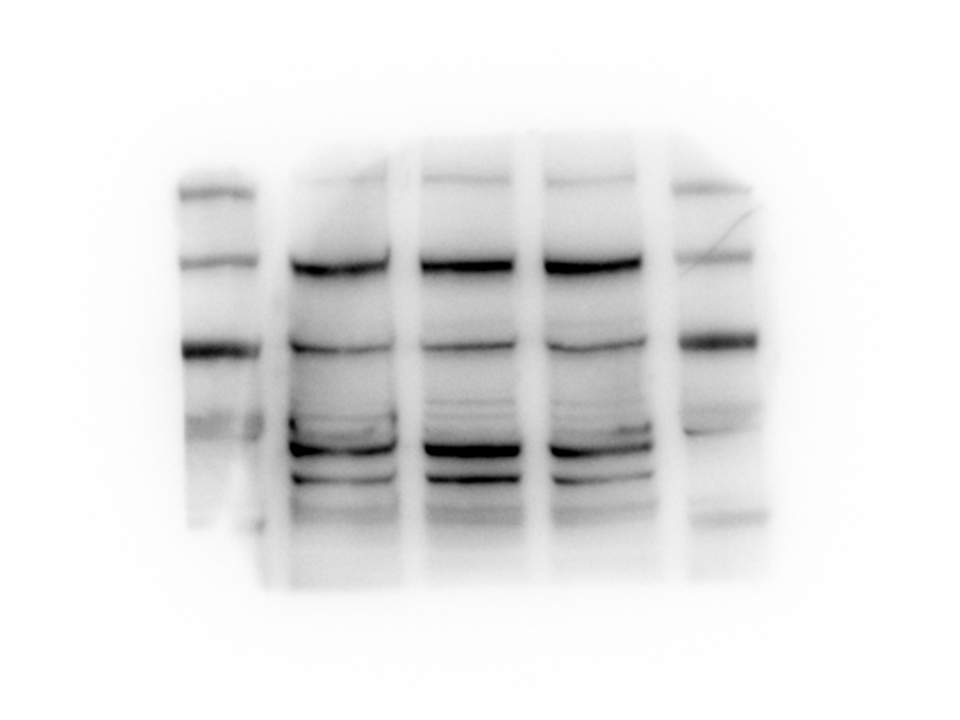

Supplement: Figure 6—source data 2. [file elife-109174-fig6-data2.zip › Fig 6,Source data2-C-Hsd11b1 OE Srebp2.tif]

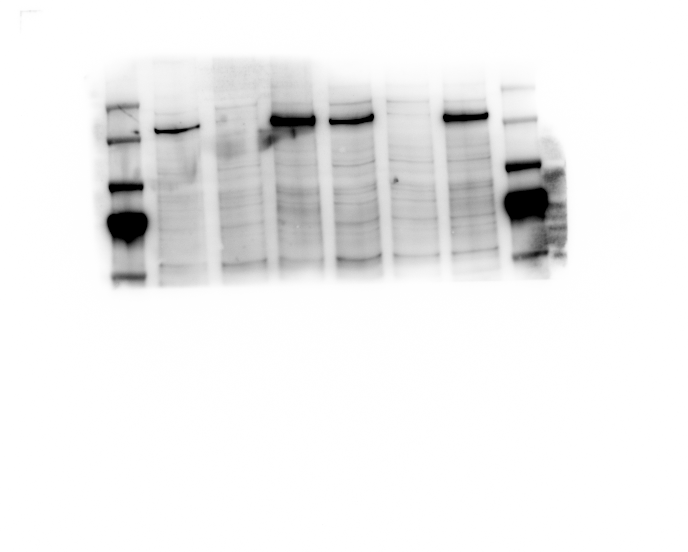

Supplement: Figure 6—source data 2. [file elife-109174-fig6-data2.zip › Fig 6,Source data2-A-WCL Anti HA.tif]

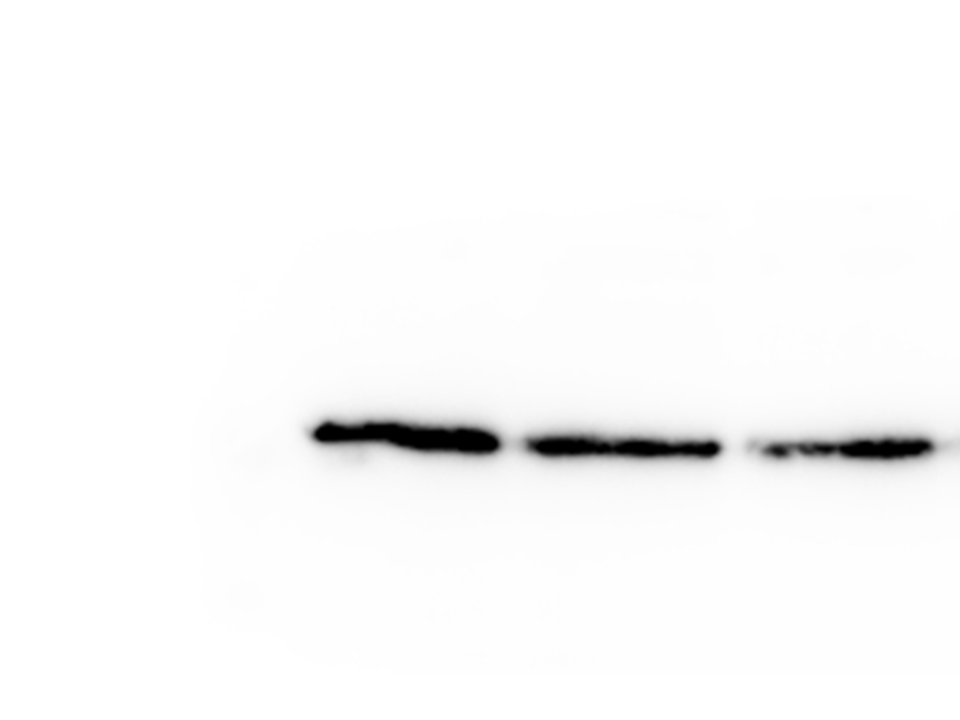

Supplement: Figure 6—source data 2. [file elife-109174-fig6-data2.zip › Fig 6,Source data2-D-Hsd11b1 KD Idi1.tif]

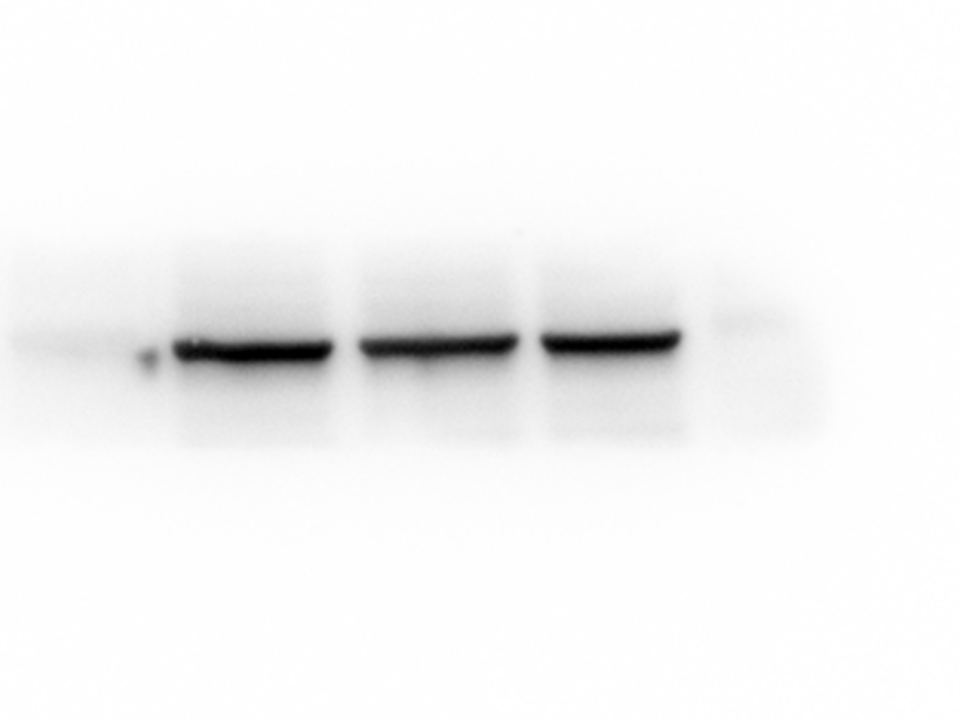

Supplement: Figure 6—source data 2. [file elife-109174-fig6-data2.zip › Fig 6,Source data2-C-Hsd11b1 OE Actin.tif]

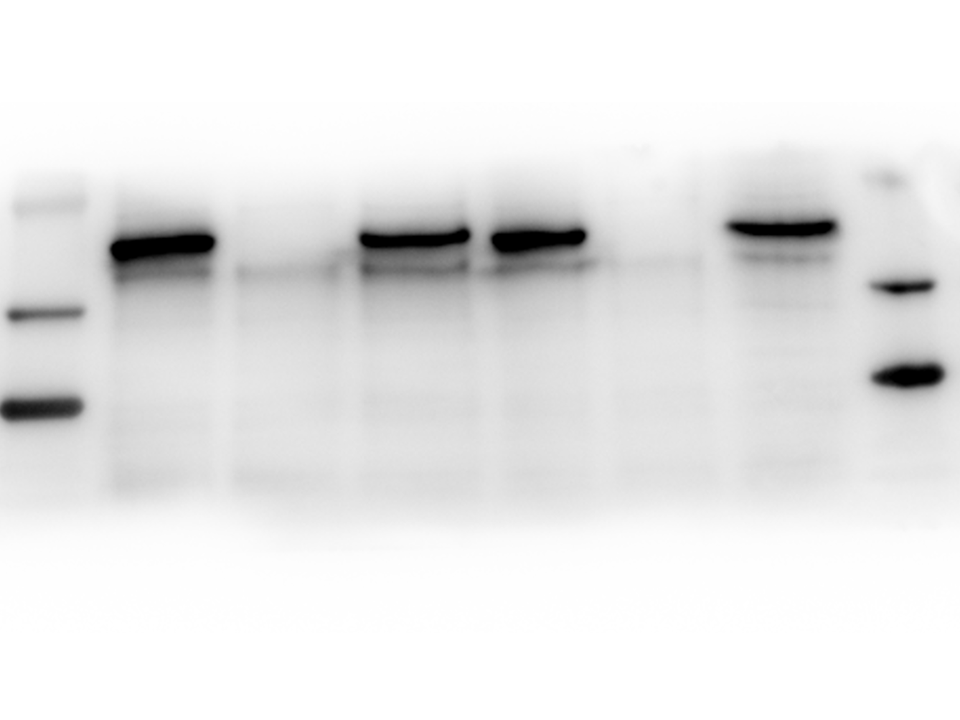

Supplement: Figure 6—source data 2. [file elife-109174-fig6-data2.zip › Fig 6,Source data2-B-WCL Anti Flag.Tif]

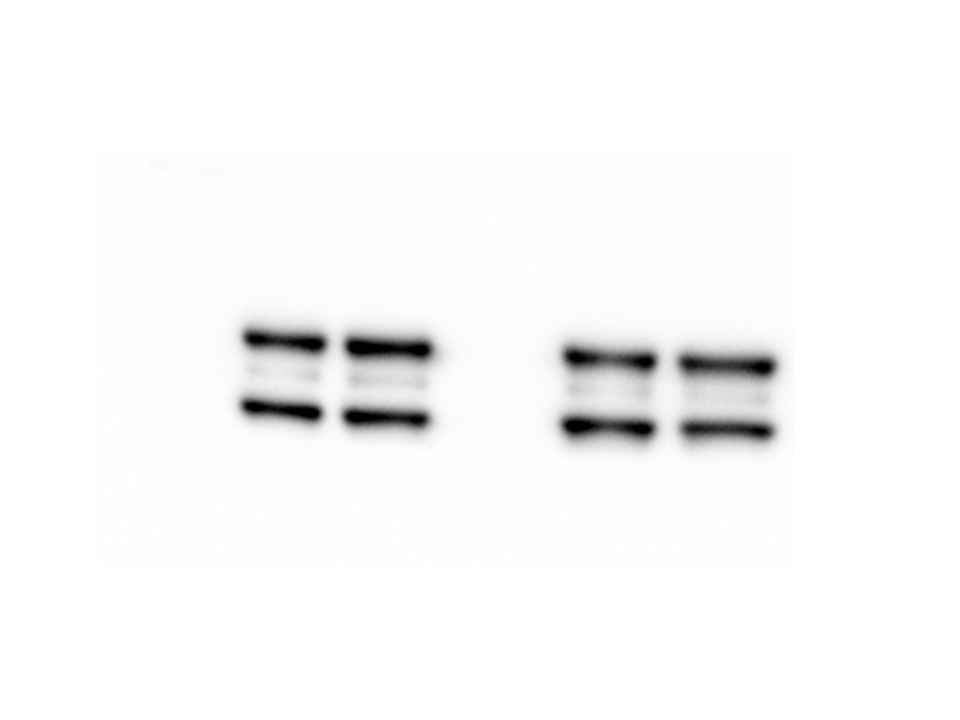

Supplement: Figure 6—source data 2. [file elife-109174-fig6-data2.zip › Fig 6,Source data2-F-IP Myc WCL anti Myc.tif]

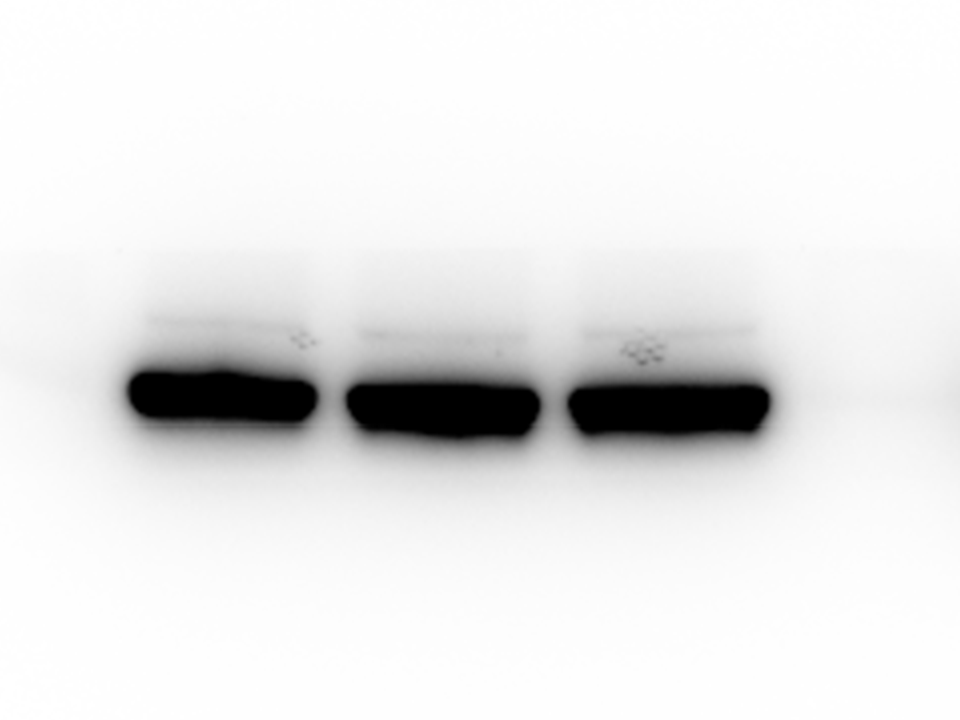

Supplement: Figure 6—source data 2. [file elife-109174-fig6-data2.zip › Fig 6,Source data2-D-Hsd11b1 KD Actin.tif]

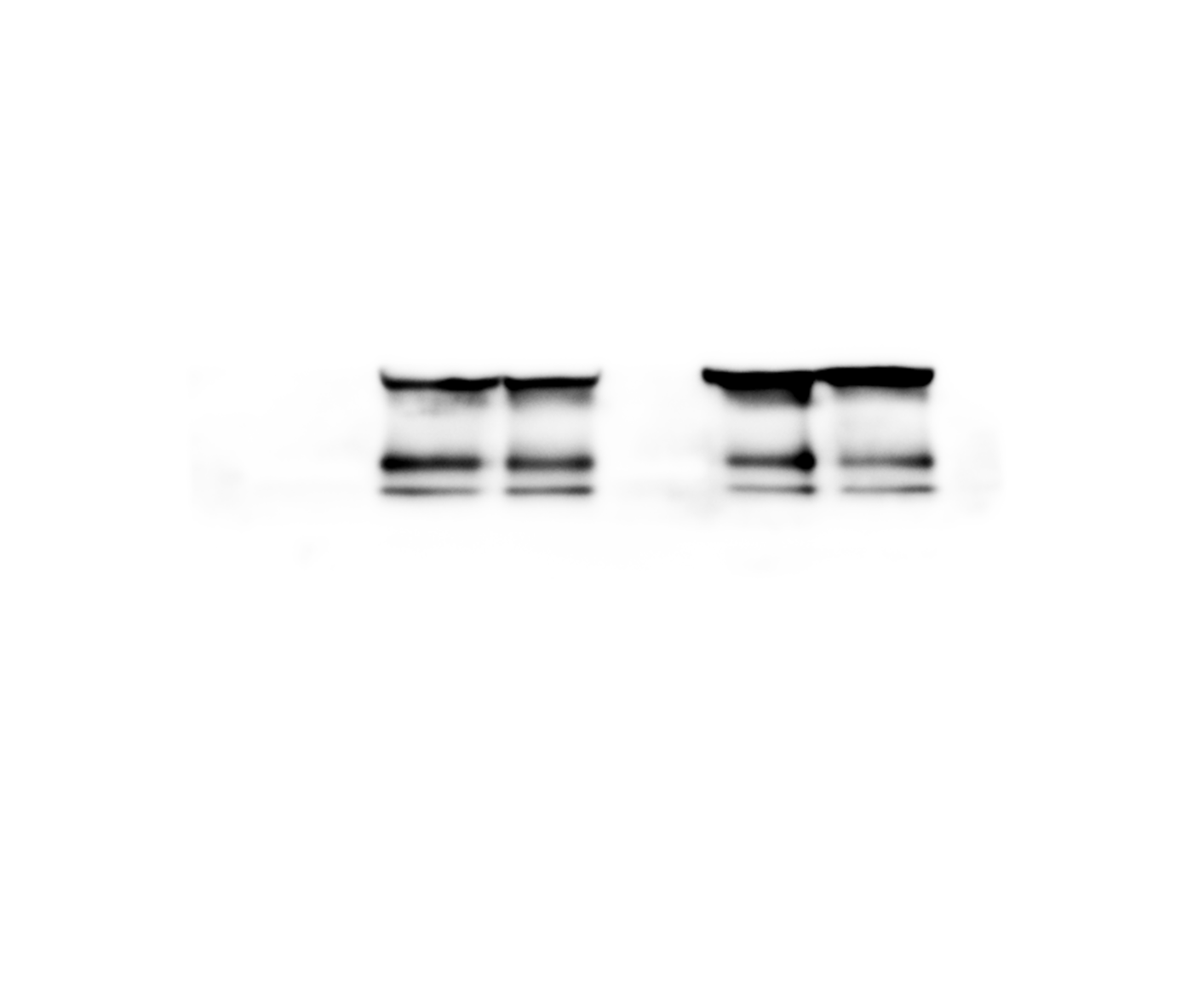

Supplement: Figure 6—source data 2. [file elife-109174-fig6-data2.zip › Fig 6,Source data2-F-IP Flag anti Flag.tif]

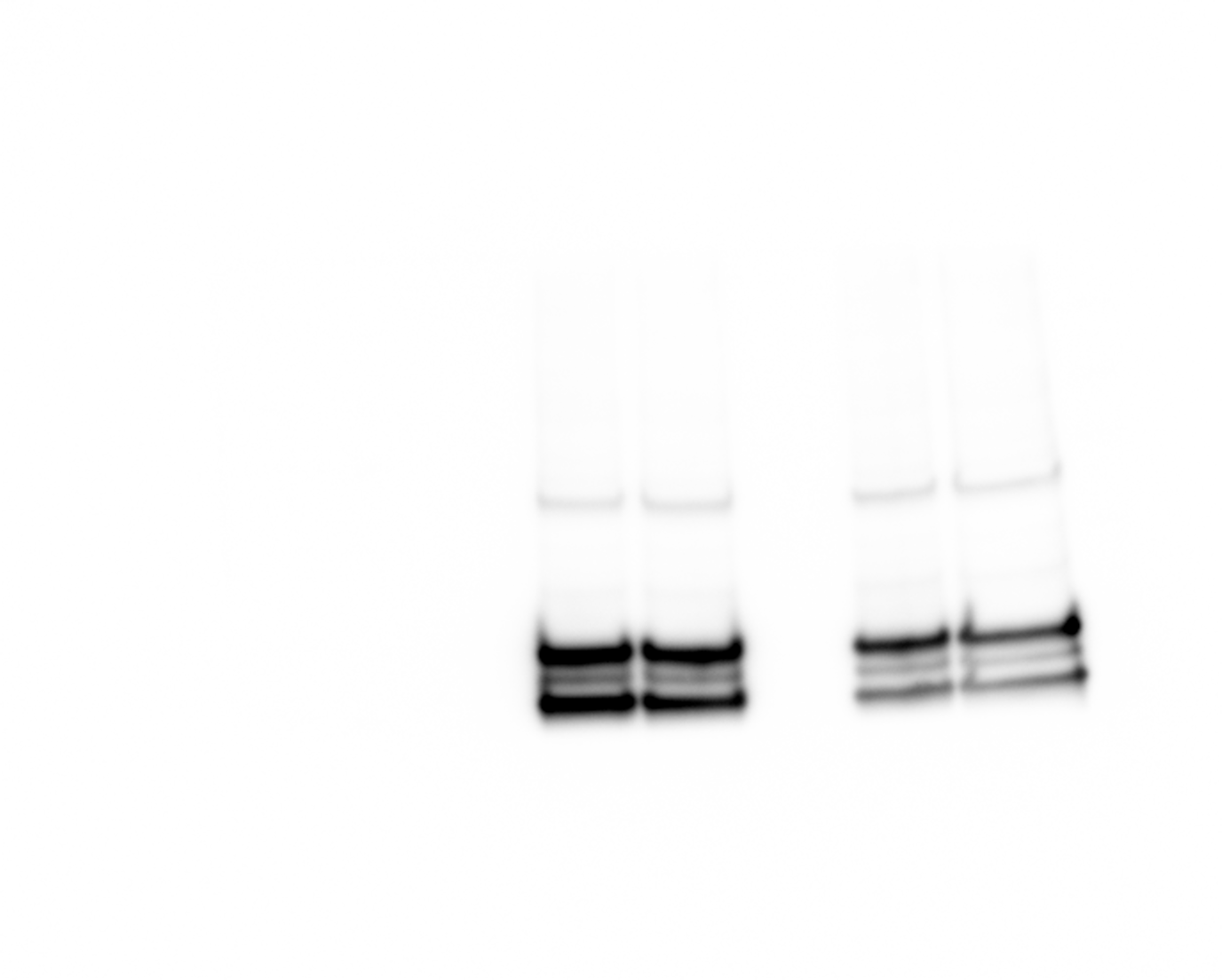

Supplement: Figure 6—source data 2. [file elife-109174-fig6-data2.zip › Fig 6,Source data2-F-IP Myc anti Myc.Tif]

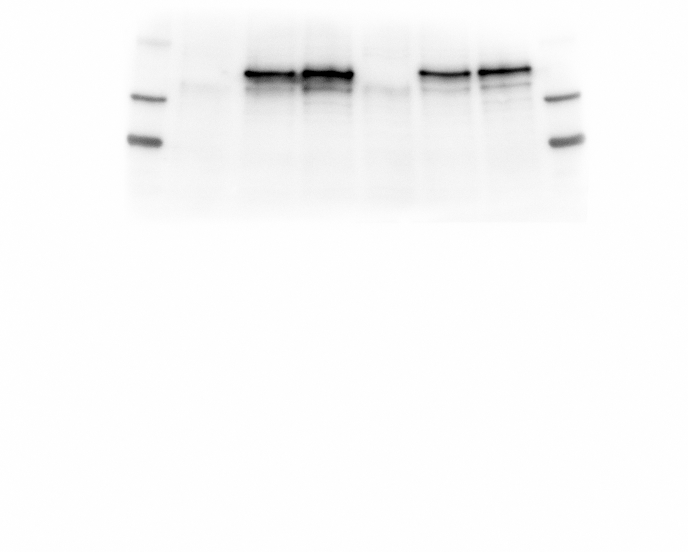

Supplement: Figure 6—source data 2. [file elife-109174-fig6-data2.zip › Fig 6,Source data2-A-WCL Anti Flag.tif]

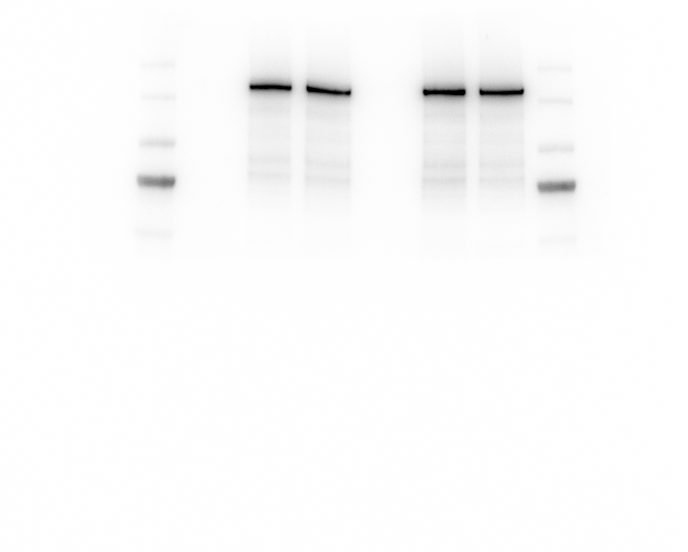

Supplement: Figure 6—source data 2. [file elife-109174-fig6-data2.zip › Fig 6,Source data2-B-IP HA Anti HA.Tif]

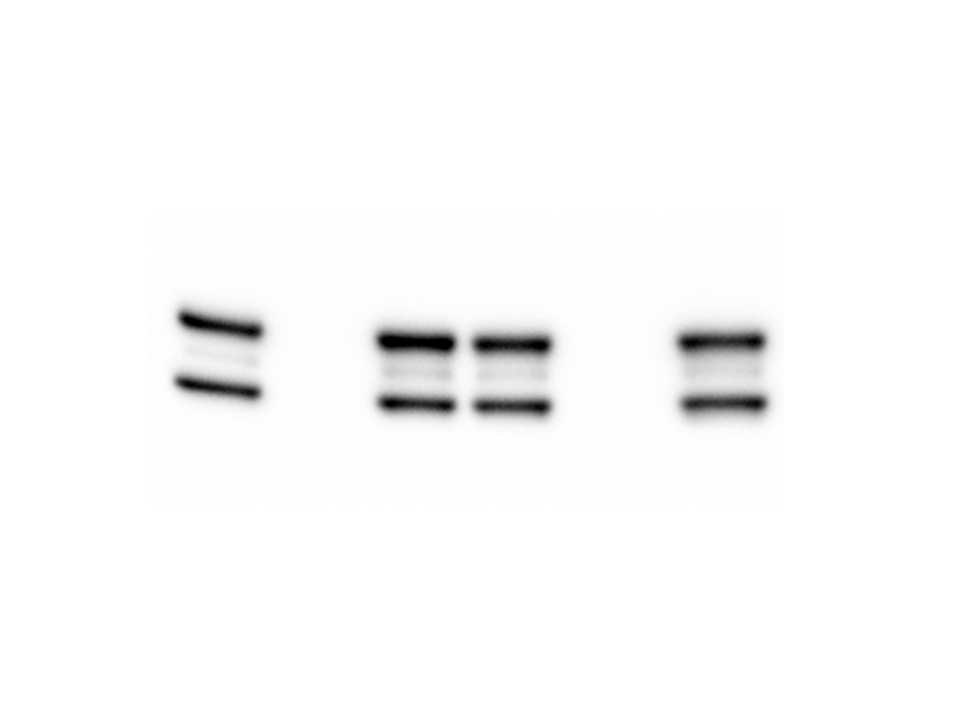

Supplement: Figure 6—source data 2. [file elife-109174-fig6-data2.zip › Fig 6,Source data2-F-IP Flag WCL anti Myc.tif]

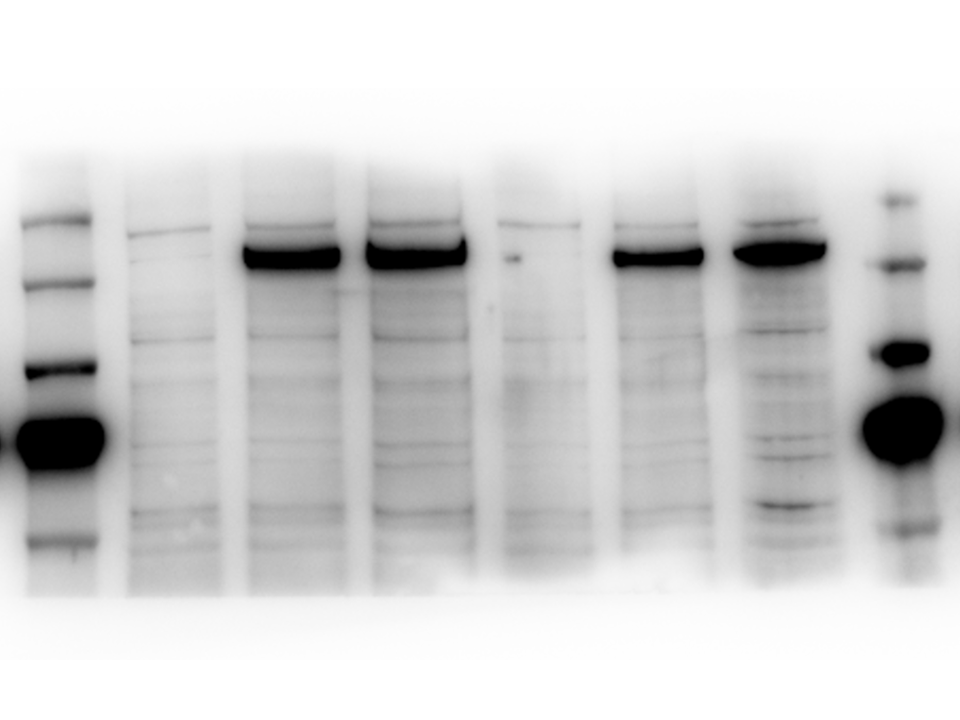

Supplement: Figure 6—source data 2. [file elife-109174-fig6-data2.zip › Fig 6,Source data2-B-WCL Anti HA.tif]

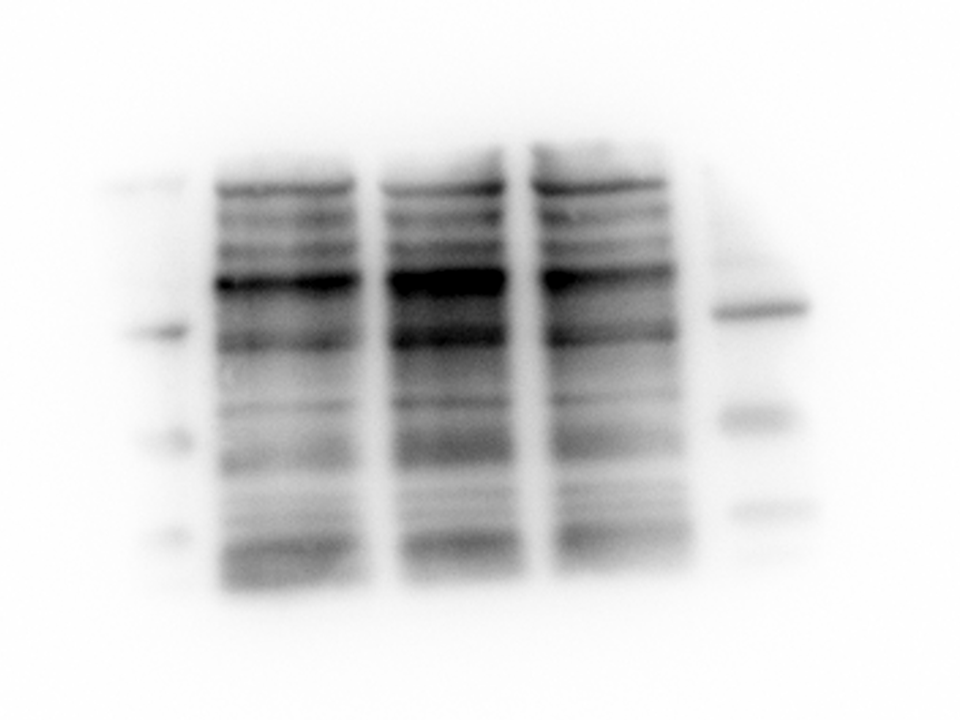

Supplement: Figure 6—source data 2. [file elife-109174-fig6-data2.zip › Fig 6,Source data2-C-Hsd11b1 OE Hsd11b1.tif]

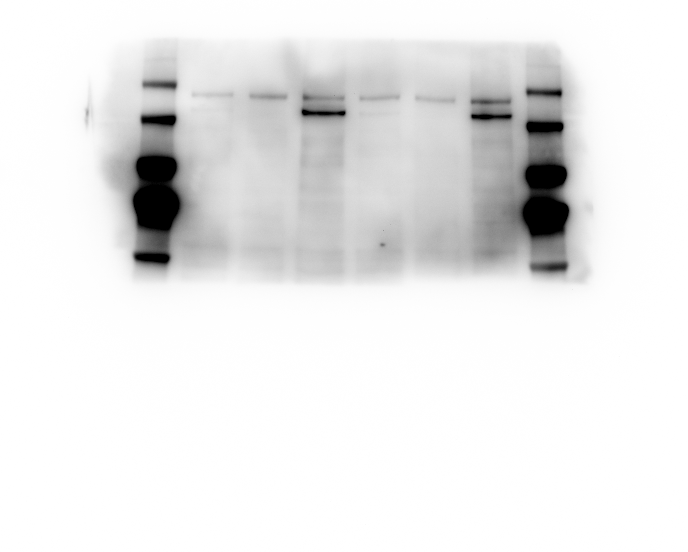

Supplement: Figure 6—source data 2. [file elife-109174-fig6-data2.zip › Fig 6,Source data2-A-IP Flag Anti HA.Tif]

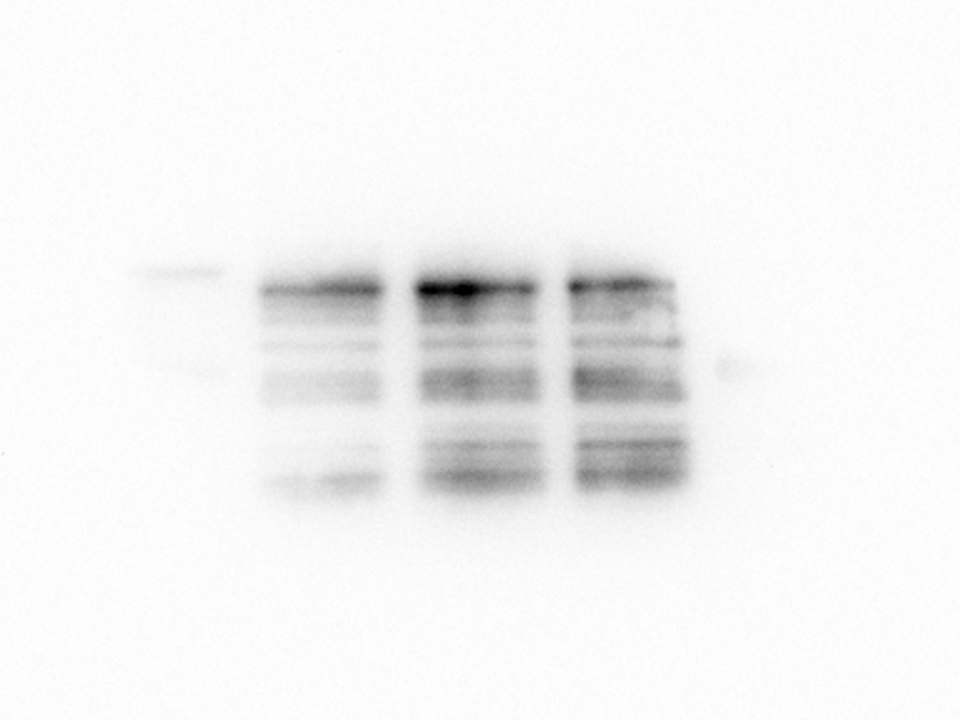

Supplement: Figure 6—source data 2. [file elife-109174-fig6-data2.zip › Fig 6,Source data2-C-Hsd11b1 OE Idi1.tif]

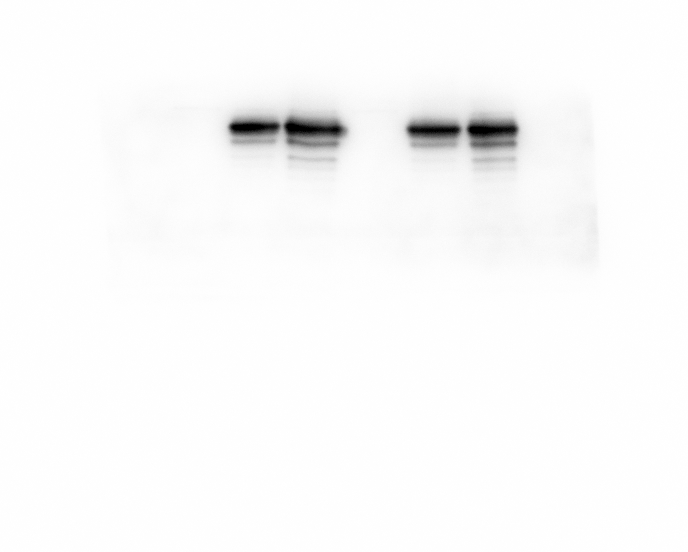

Supplement: Figure 6—source data 2. [file elife-109174-fig6-data2.zip › Fig 6,Source data2-A-IP Flag Anti Flag.Tif]

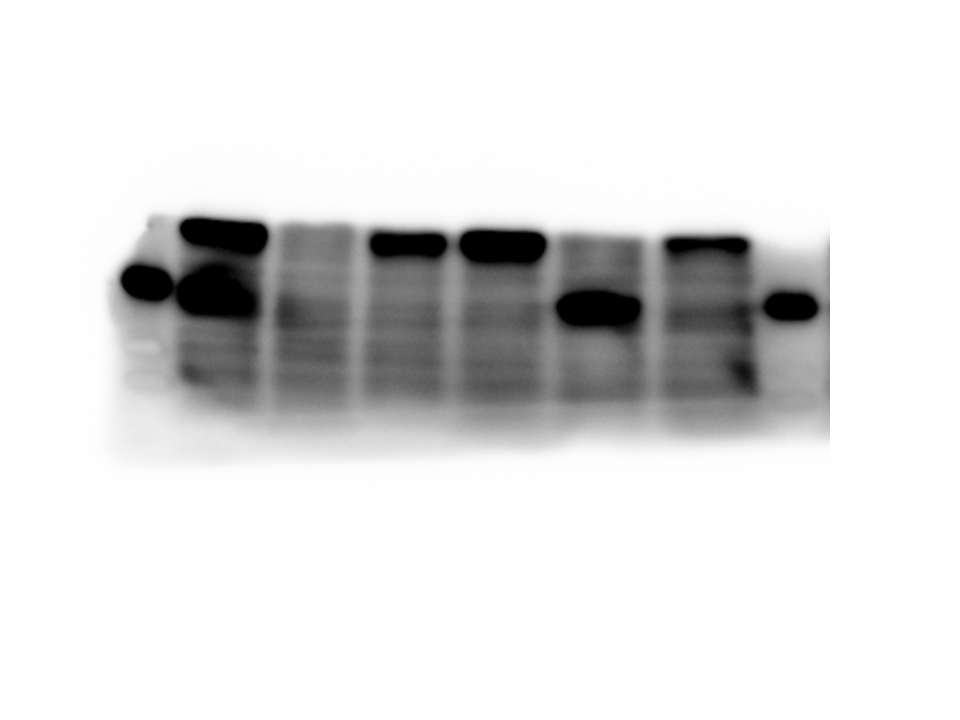

Supplement: Figure 6—source data 2. [file elife-109174-fig6-data2.zip › Fig 6,Source data2-F-IP Myc WCL anti Flag.tif]

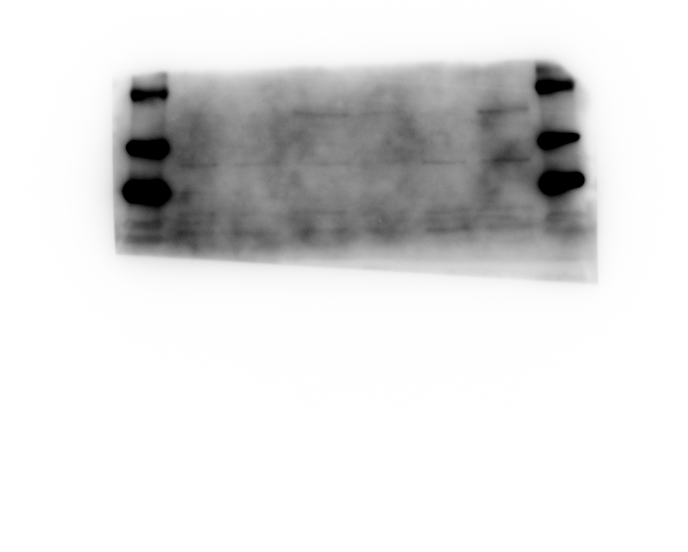

Supplement: Figure 6—source data 2. [file elife-109174-fig6-data2.zip › Fig 6,Source data2-B-IP HA Anti Flag.Tif]

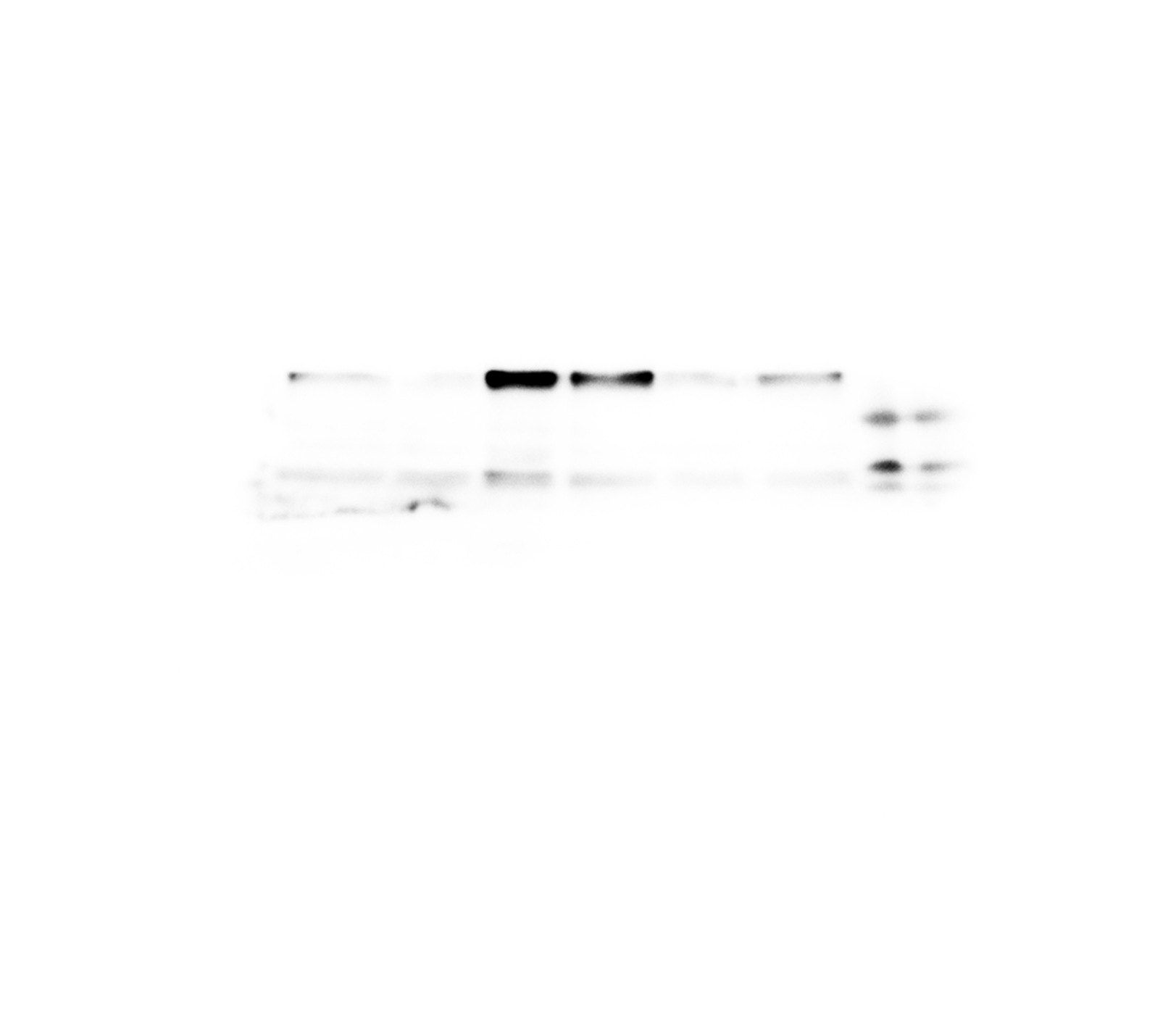

Supplement: Figure 6—source data 2. [file elife-109174-fig6-data2.zip › Fig 6,Source data2-F-IP Myc anti Flag.tif]

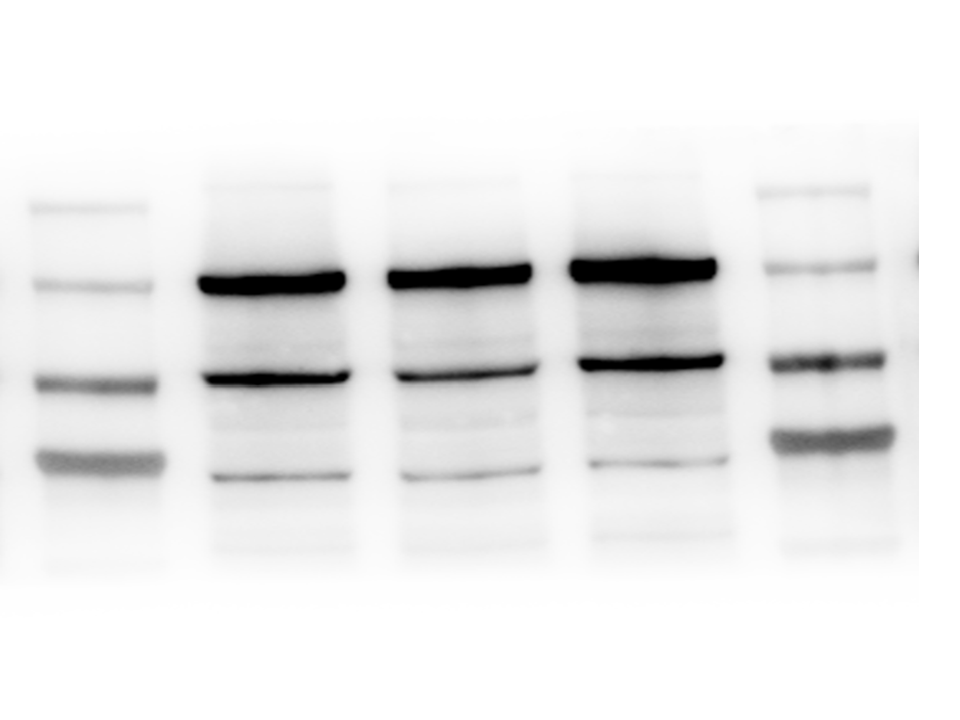

Supplement: Figure 6—source data 2. [file elife-109174-fig6-data2.zip › Fig 6,Source data2-D-Hsd11b1 KD Srebp2.tif]

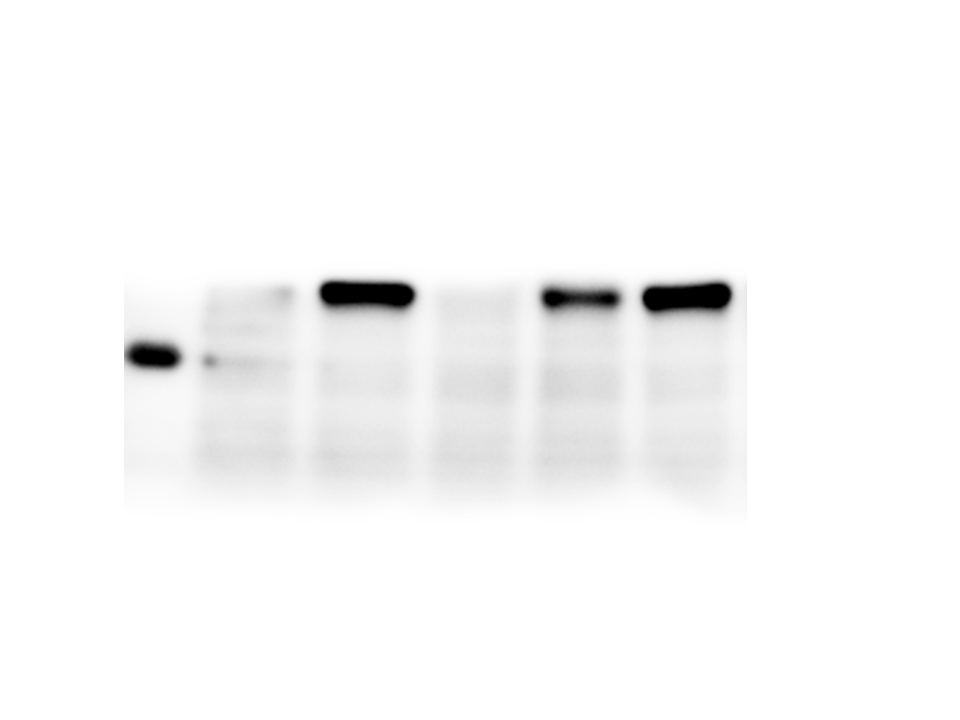

Supplement: Figure 6—source data 2. [file elife-109174-fig6-data2.zip › Fig 6,Source data2-F-IP Flag WCL anti Flag.tif]

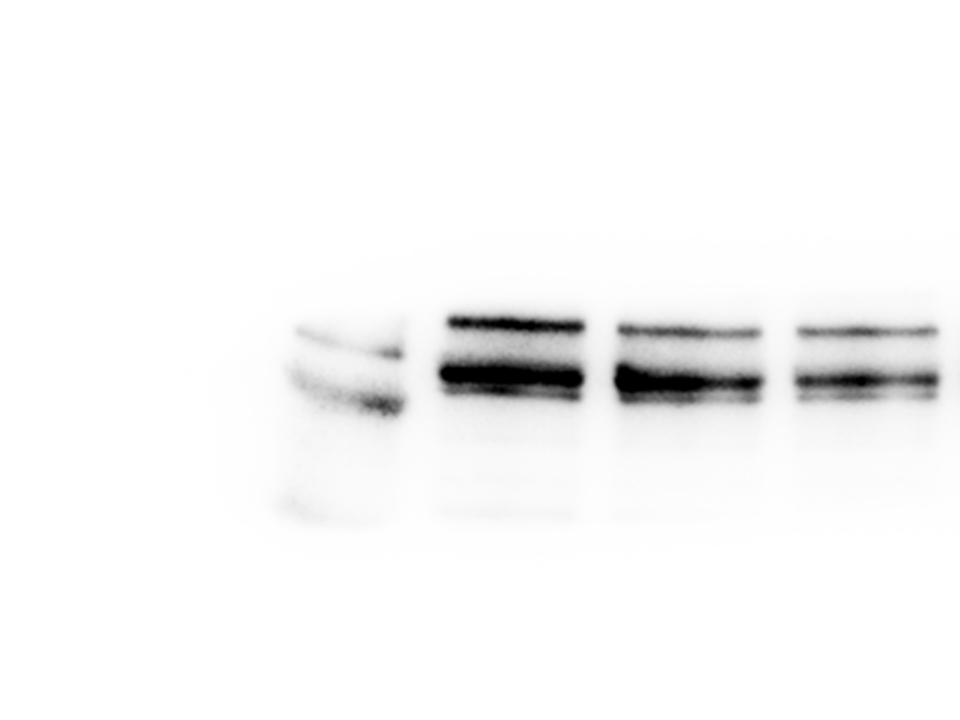

Supplement: Figure 6—source data 2. [file elife-109174-fig6-data2.zip › Fig 6,Source data2-D-Hsd11b1 KD Hsd11b1.tif]

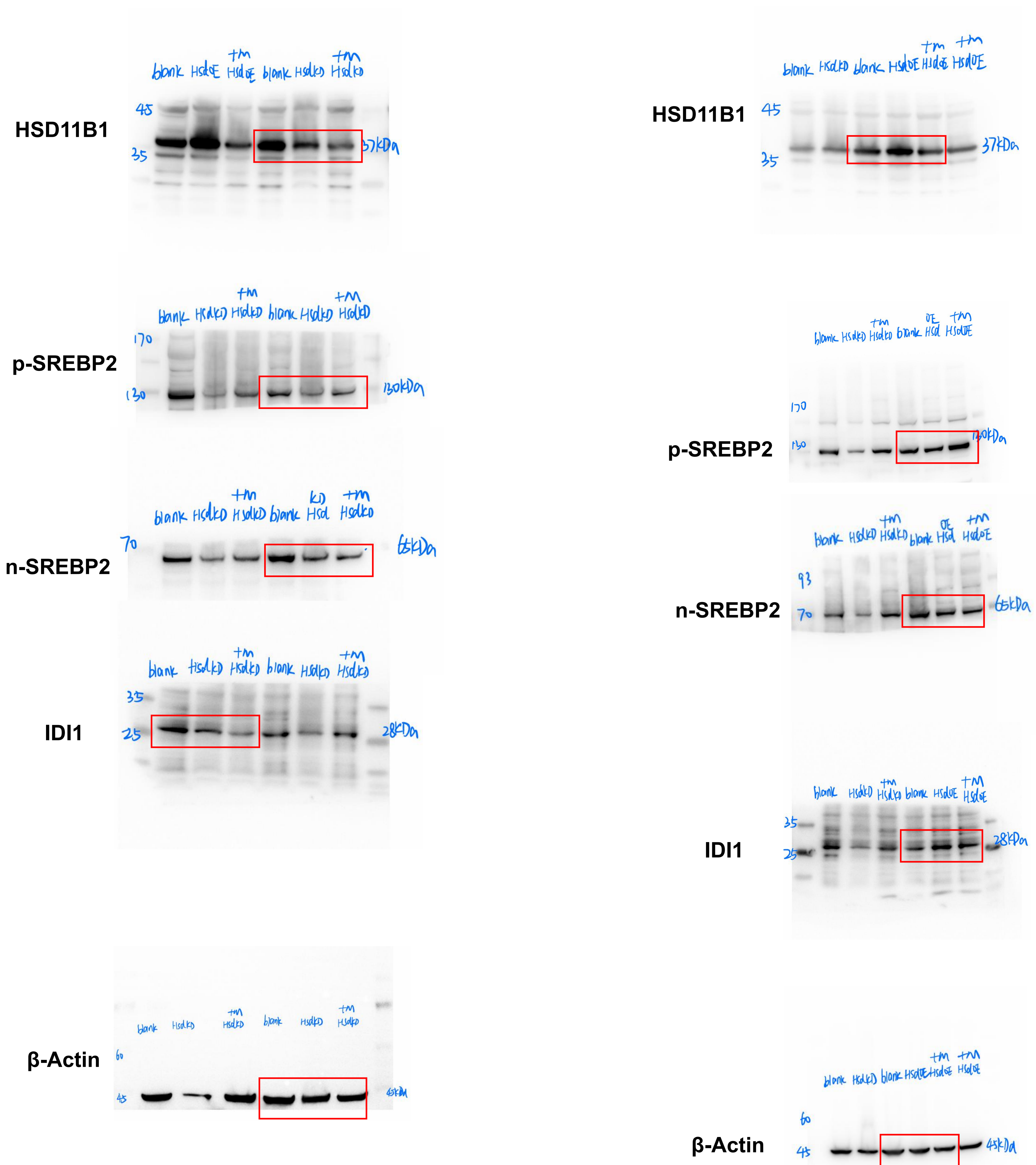

**Fig 7, Source Data 1.** Original membranes corresponding to Figure 7

Supplement: Figure 7—source data 1. [file elife-109174-fig7-data1.zip › Figure 7-Source PDF.pdf]

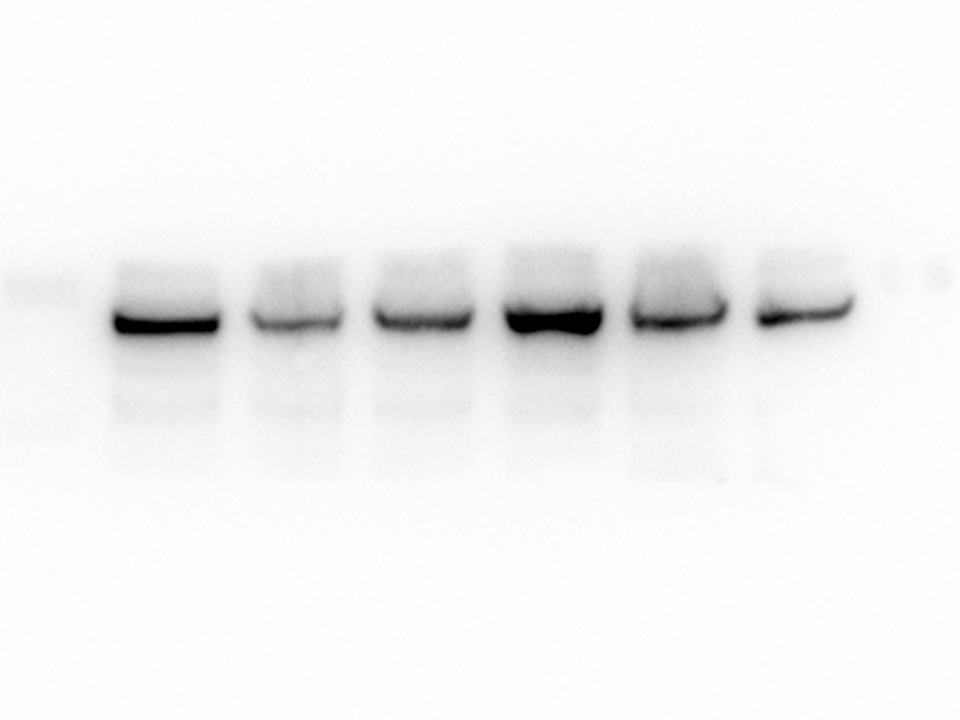

Supplement: Figure 7—source data 2. [file elife-109174-fig7-data2.zip › Fig 7,Source data2-Hsd11b1KD n-srebp2.tif]

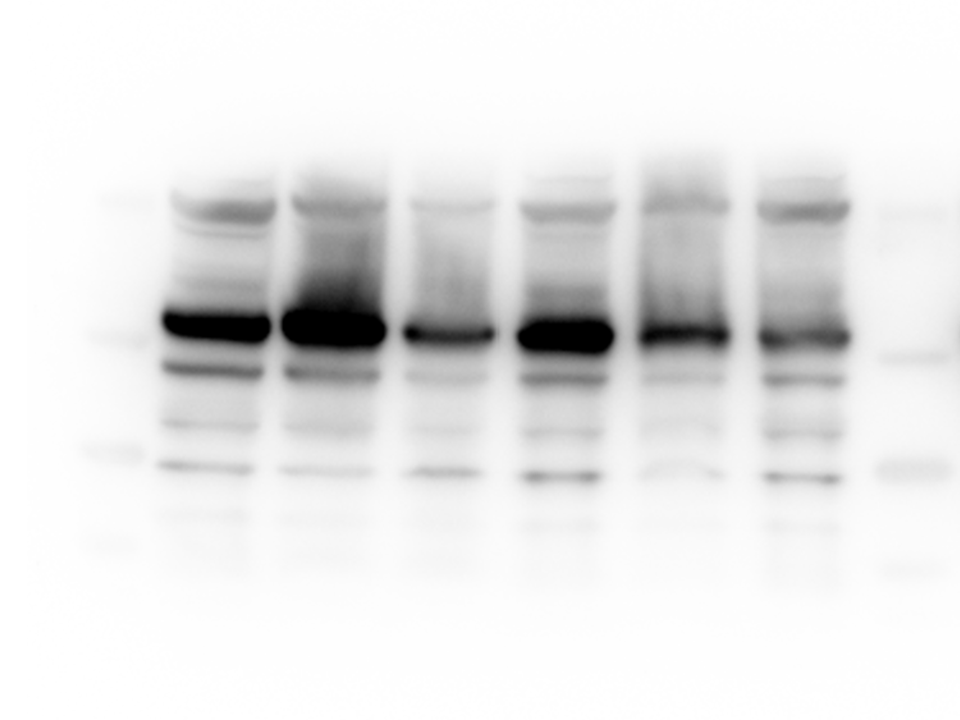

Supplement: Figure 7—source data 2. [file elife-109174-fig7-data2.zip › Fig 7,Source data2-Hsd11b1KD Hsd11b1.tif]

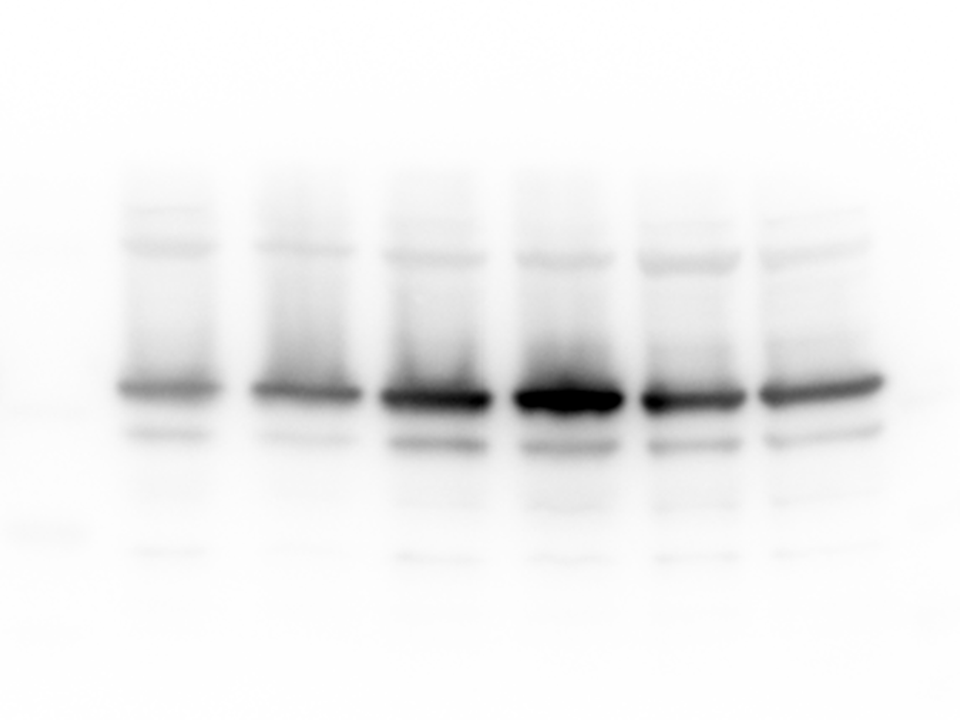

Supplement: Figure 7—source data 2. [file elife-109174-fig7-data2.zip › Fig 7,Source data2-Hsd11b1OE Hsd11b1.tif]

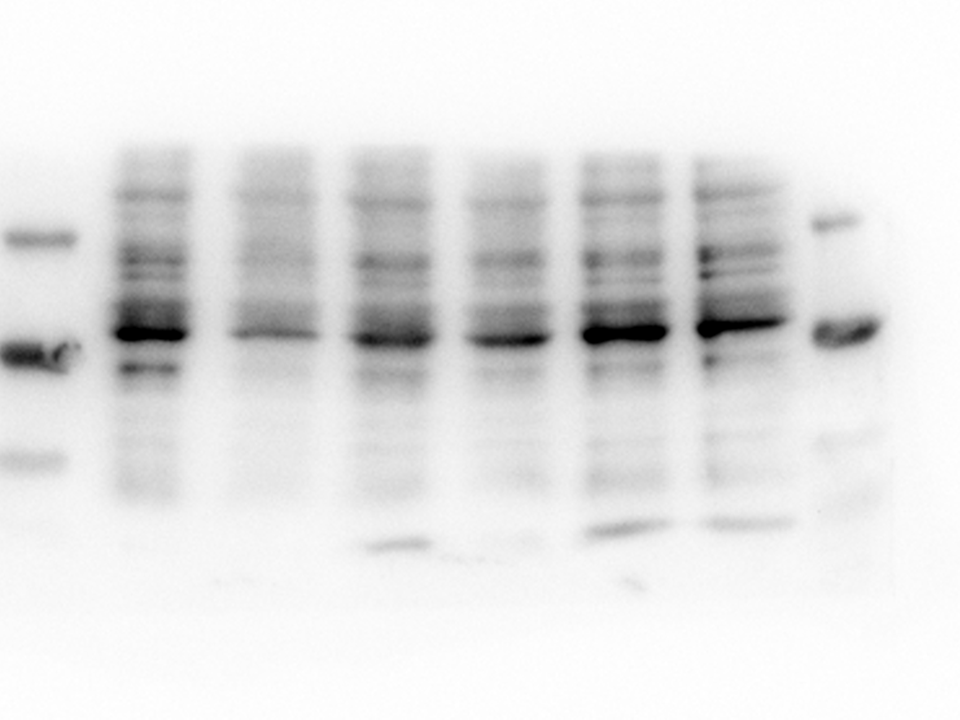

Supplement: Figure 7—source data 2. [file elife-109174-fig7-data2.zip › Fig 7,Source data2-Hsd11b1OE Idi1.tif]

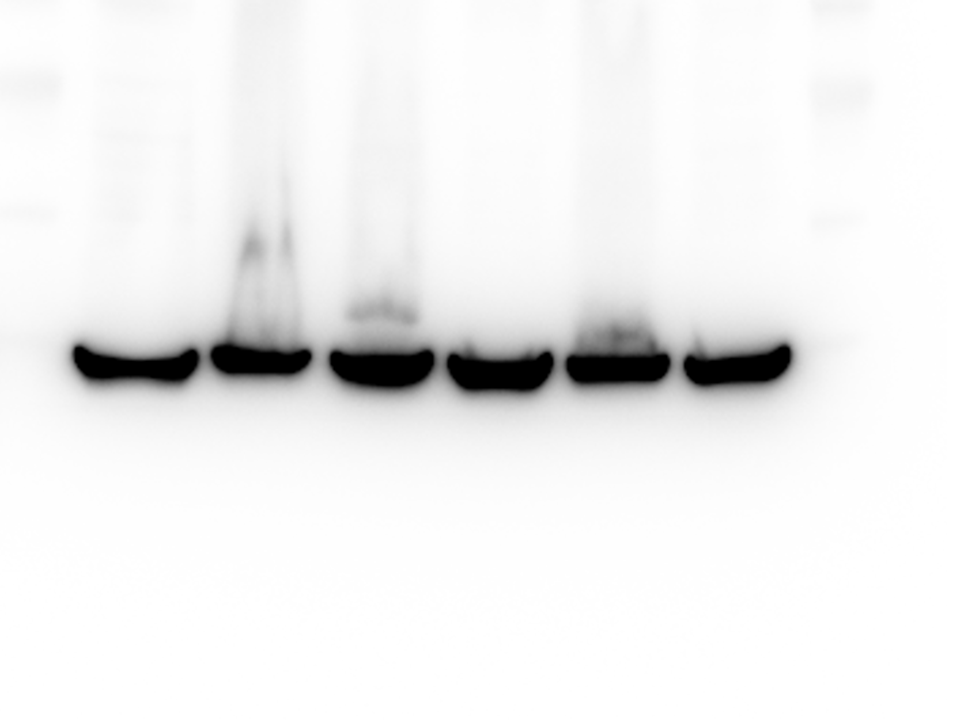

Supplement: Figure 7—source data 2. [file elife-109174-fig7-data2.zip › Fig 7,Source data2-Hsd11b1KD Actin.tif]

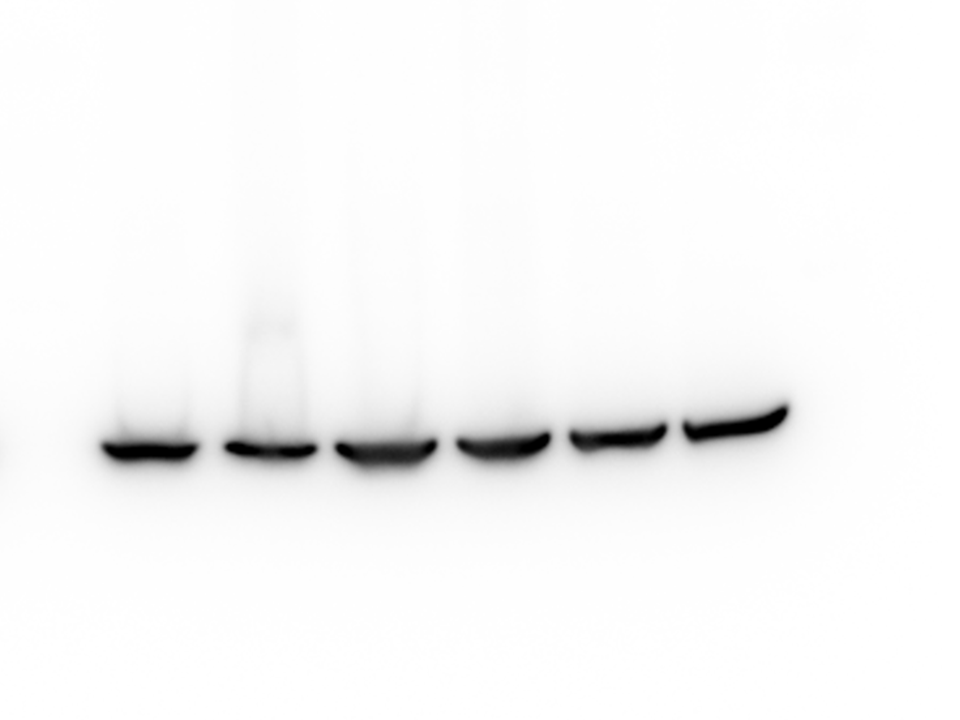

Supplement: Figure 7—source data 2. [file elife-109174-fig7-data2.zip › Fig 7,Source data2-Hsd11b1OE Actin.tif]

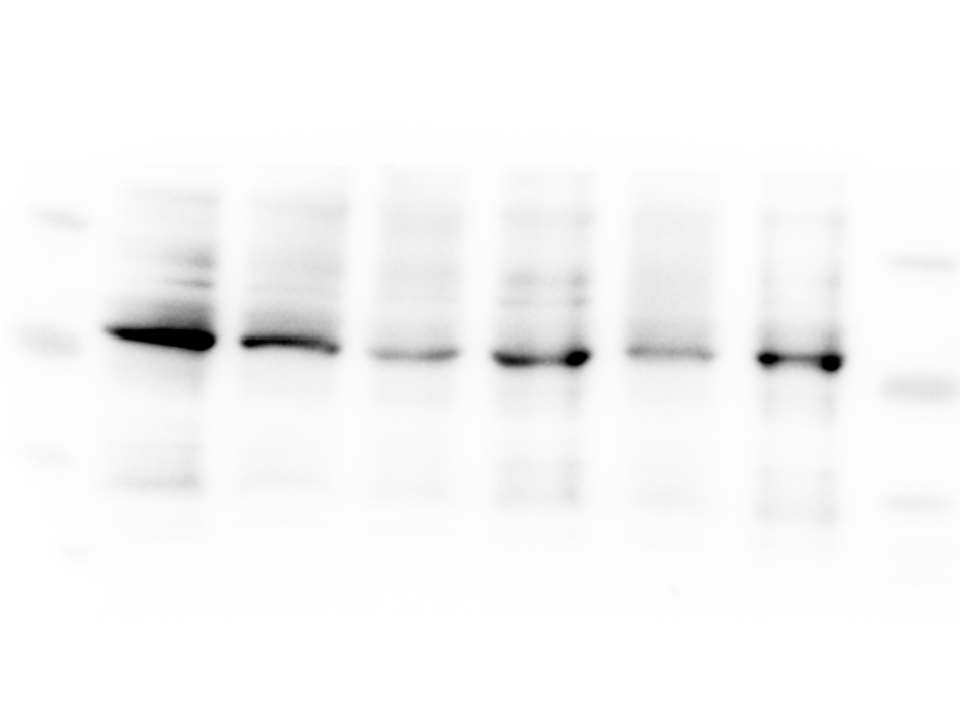

Supplement: Figure 7—source data 2. [file elife-109174-fig7-data2.zip › Fig 7,Source data2-Hsd11b1KD Idi1.tif]

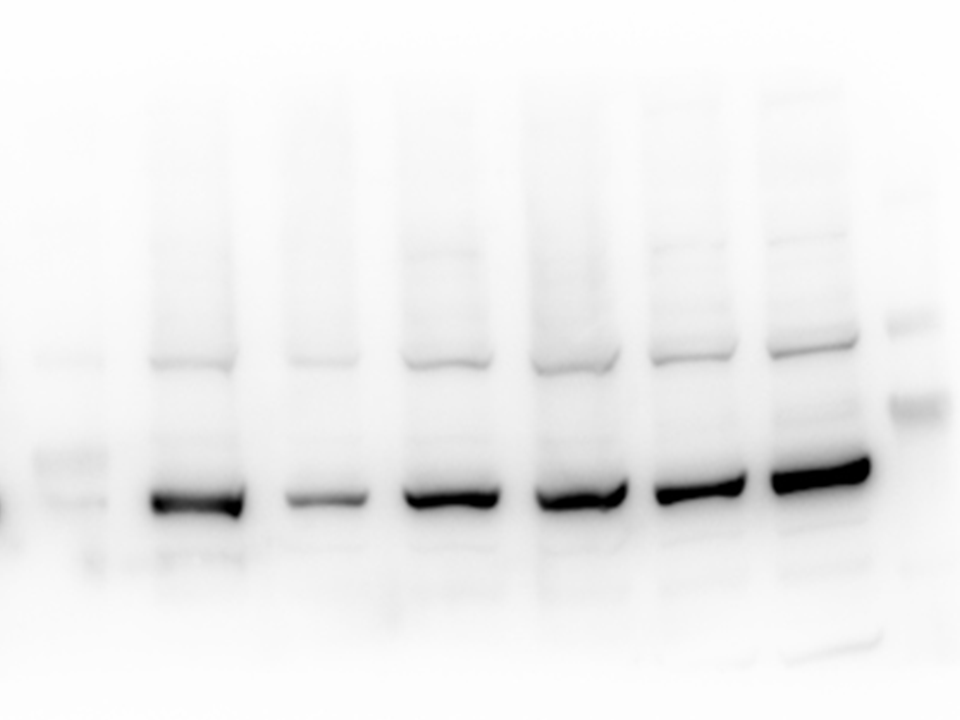

Supplement: Figure 7—source data 2. [file elife-109174-fig7-data2.zip › Fig 7,Source data2-Hsd11b1OE p-srebp2.tif]

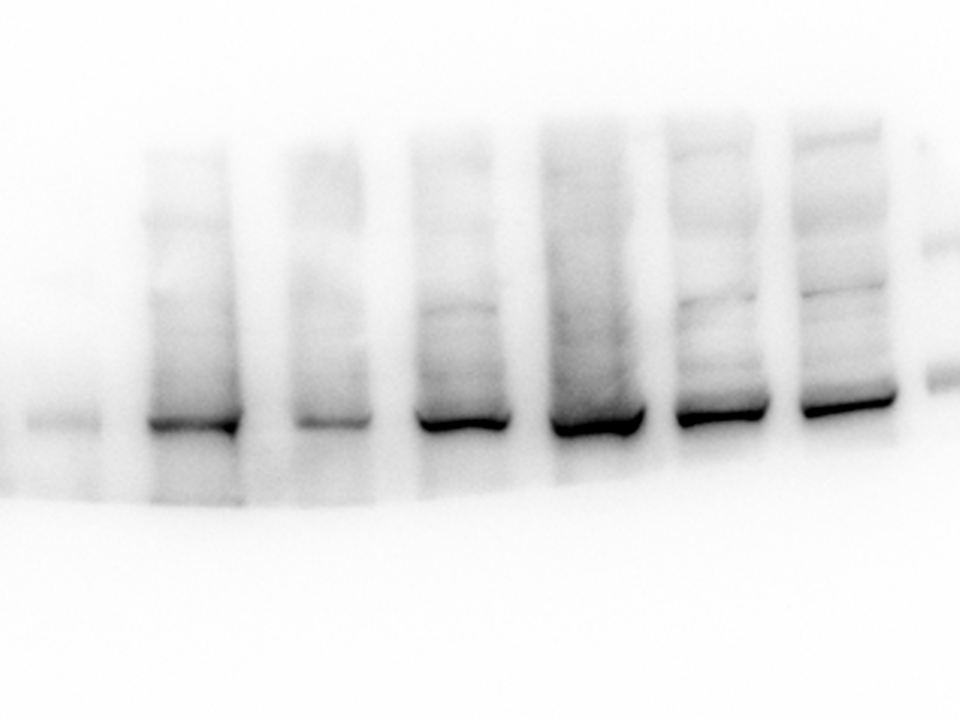

Supplement: Figure 7—source data 2. [file elife-109174-fig7-data2.zip › Fig 7,Source data2-Hsd11b1OE n-srebp2.tif]

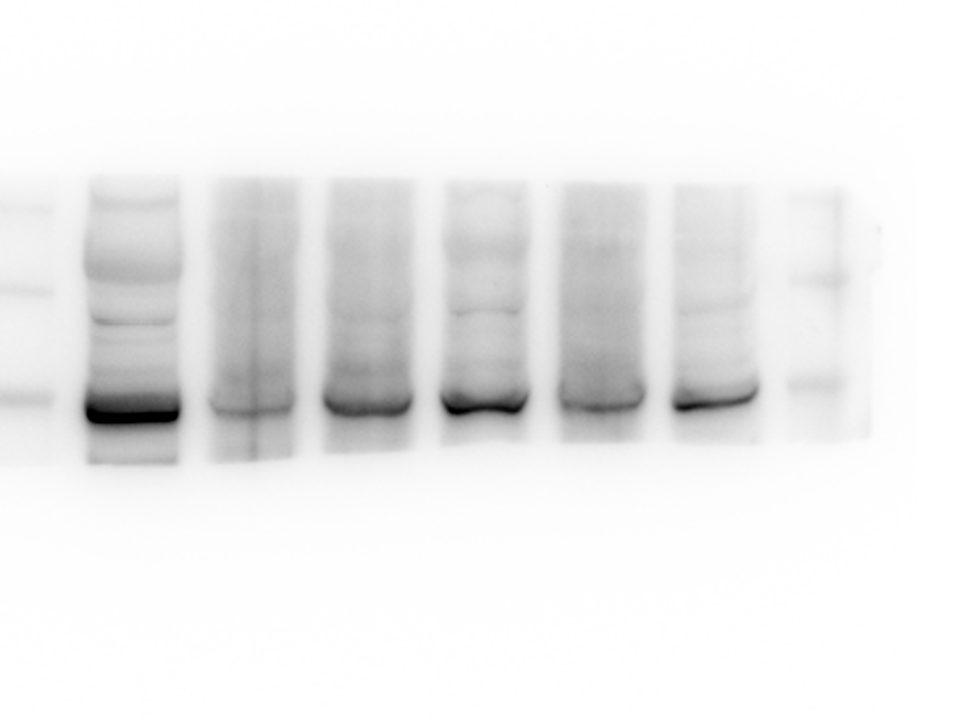

Supplement: Figure 7—source data 2. [file elife-109174-fig7-data2.zip › Fig 7,Source data2-Hsd11b1KD p-srebp2.tif]

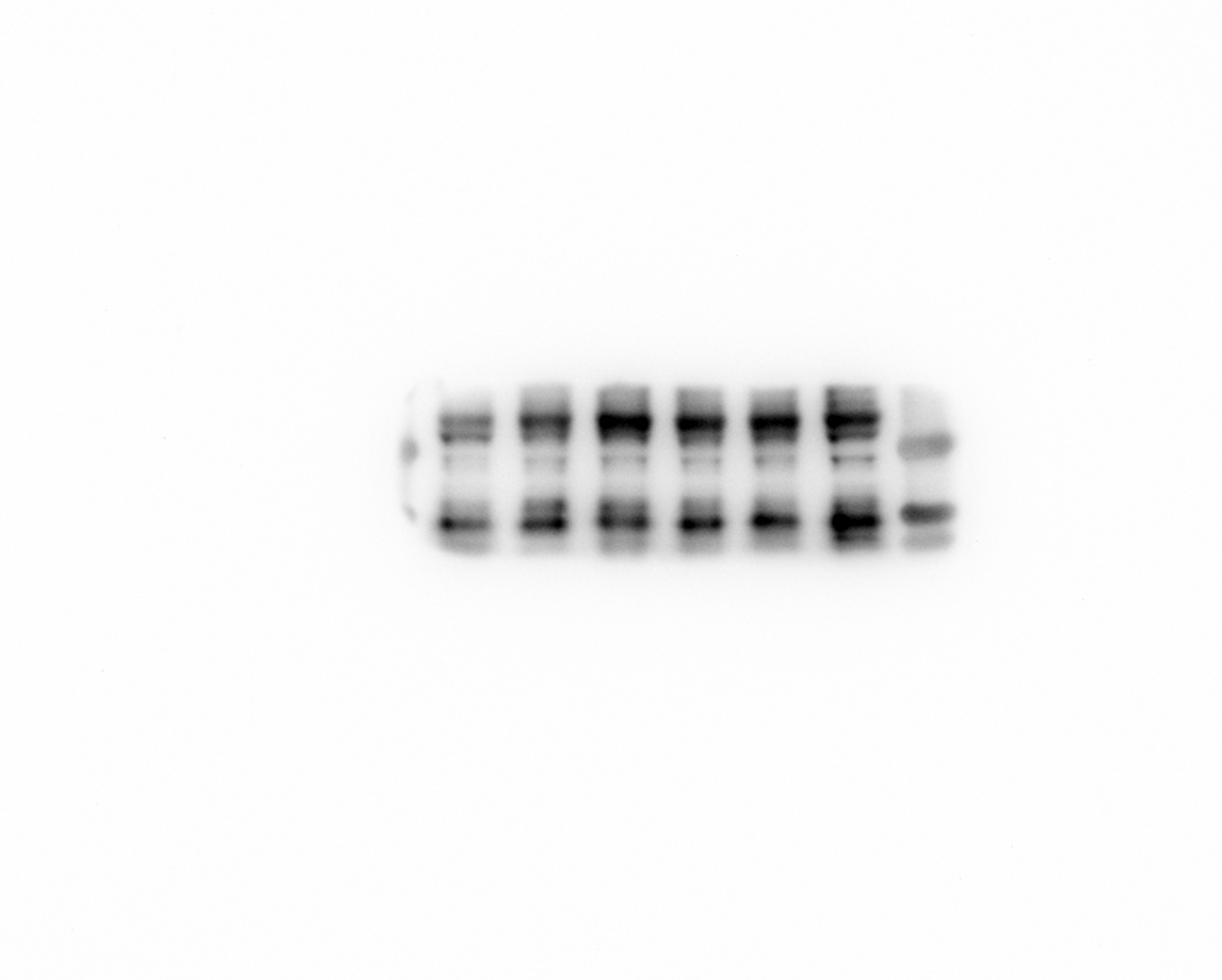

Supplement: Figure 7—figure supplement 1—source data 2. [file elife-109174-fig7-figsupp1-data2.zip › Figure 7-figure supplement 1-source data 2-Idi1-OE.tif]

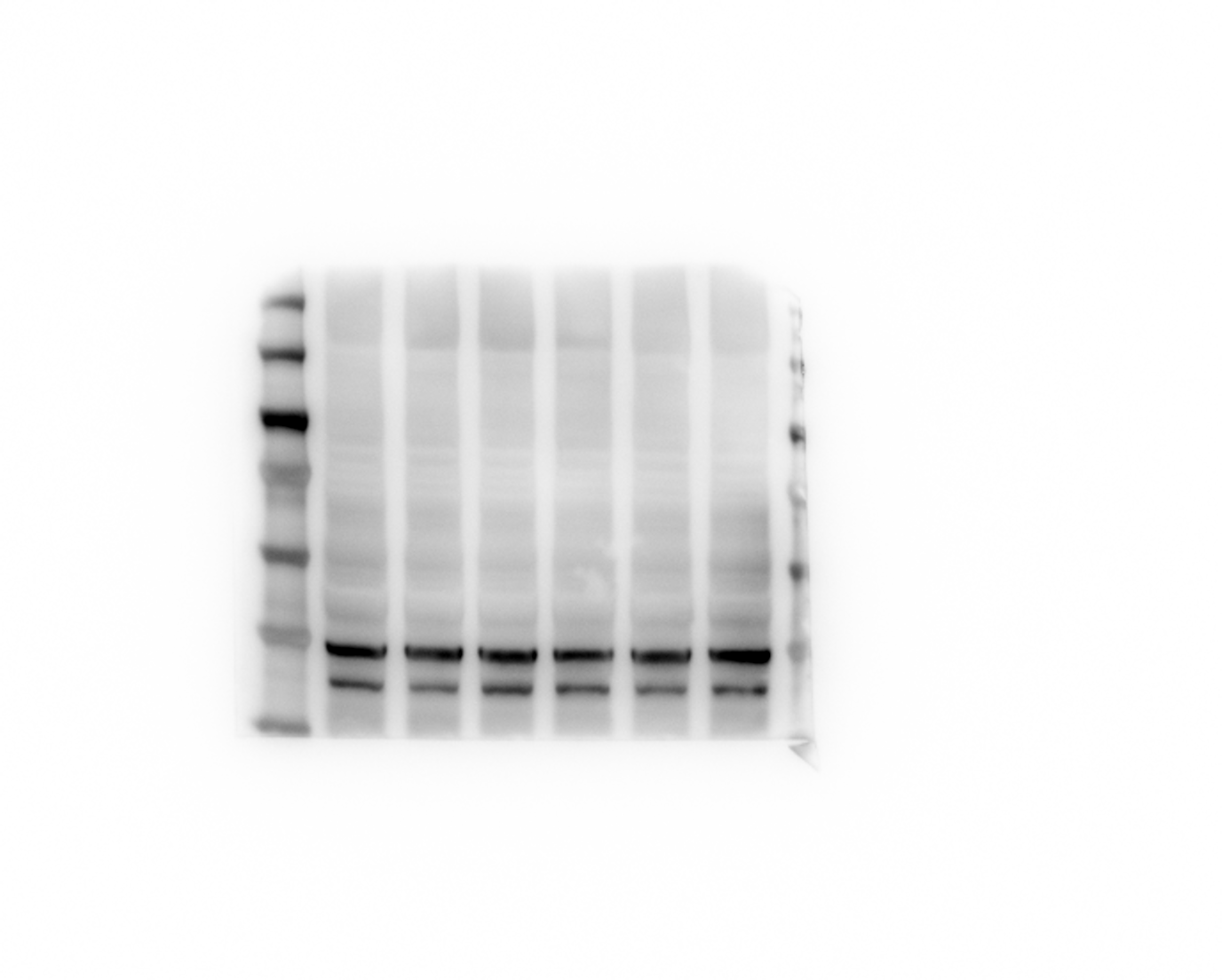

Supplement: Figure 7—figure supplement 1—source data 2. [file elife-109174-fig7-figsupp1-data2.zip › Figure 7-figure supplement 1-source data 2-Actin-Idi1-OE.Tif]

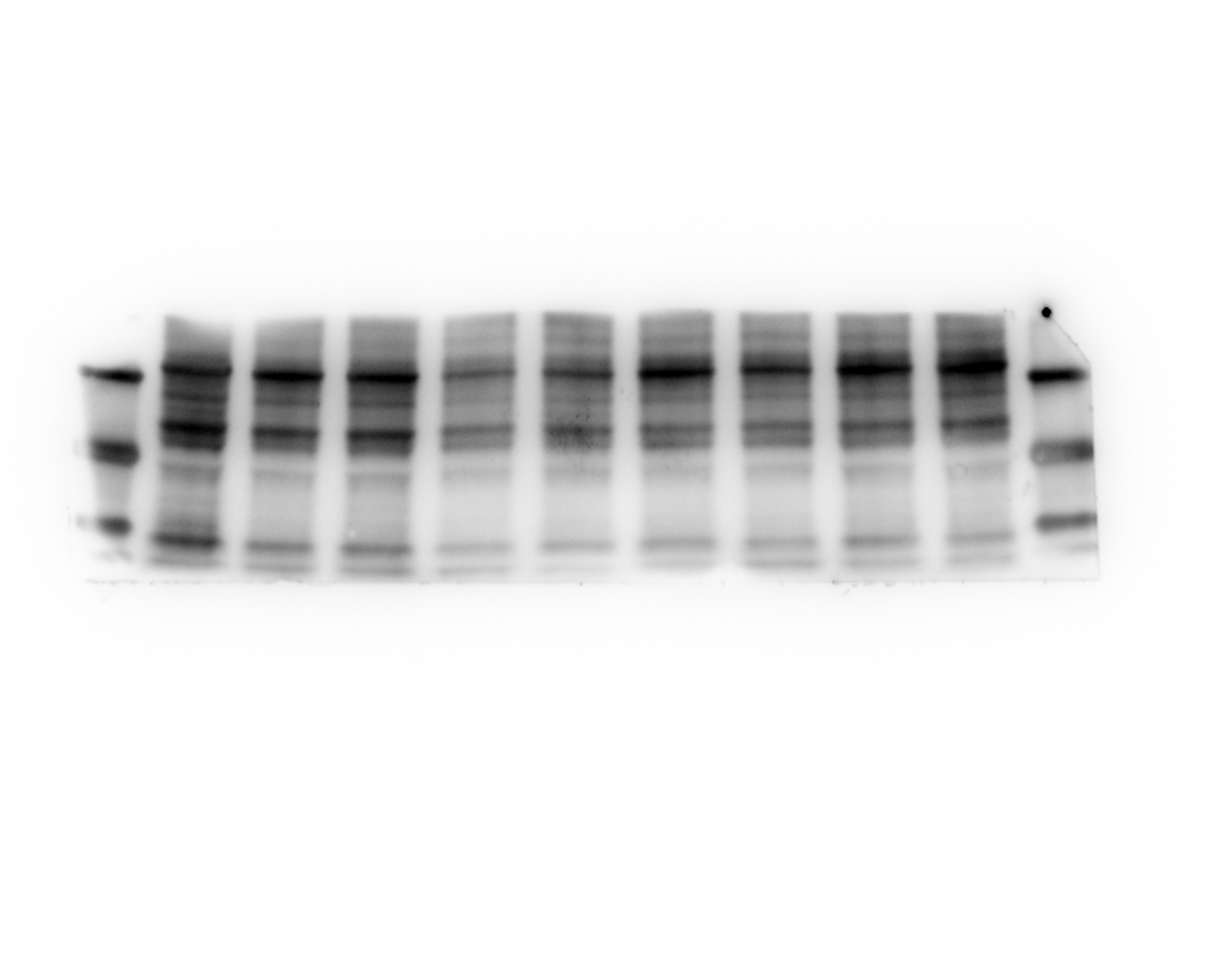

Supplement: Figure 7—figure supplement 1—source data 2. [file elife-109174-fig7-figsupp1-data2.zip › Figure 7-figure supplement 1-source data 2-Hsd11b1-KD.Tif]

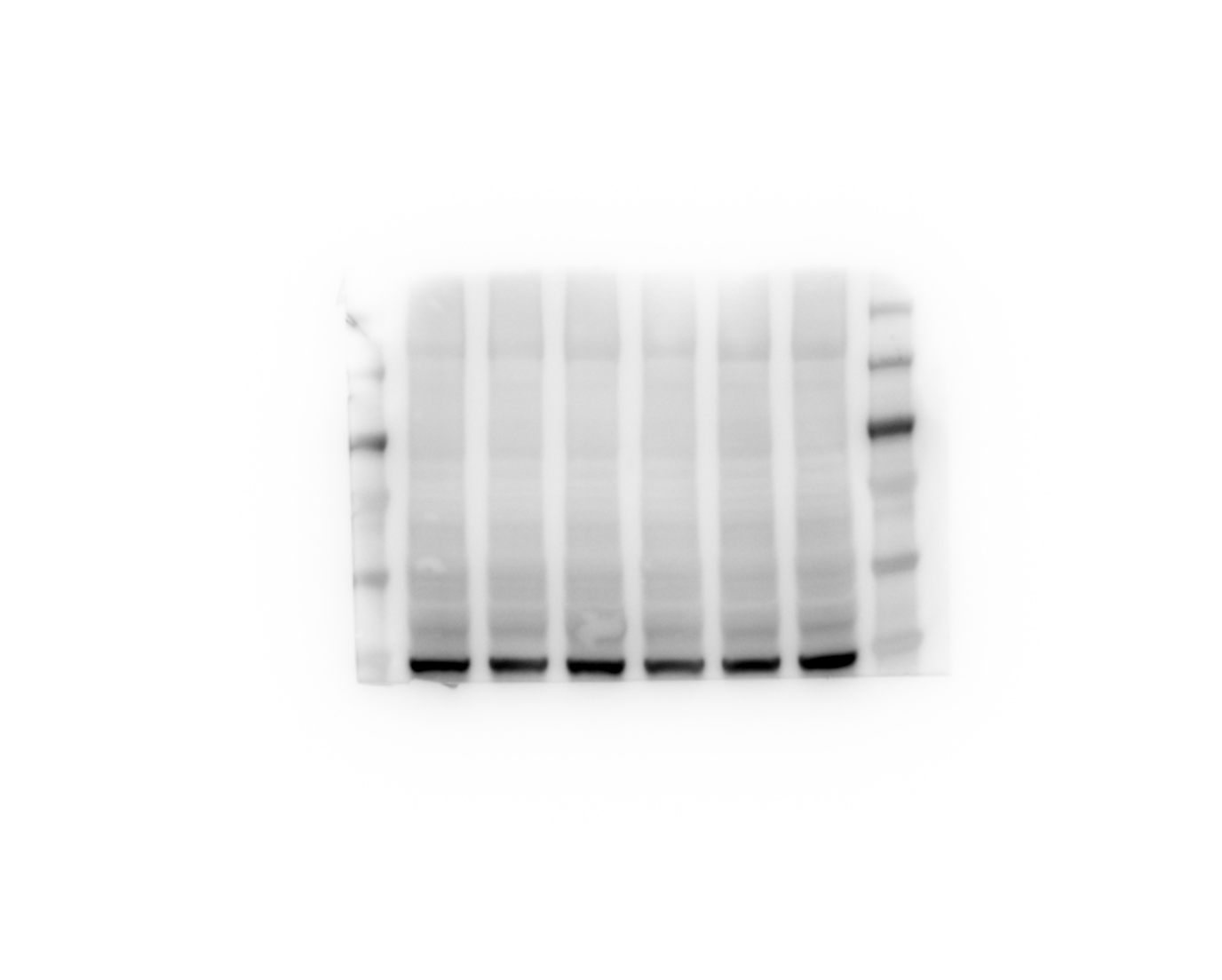

Supplement: Figure 7—figure supplement 1—source data 2. [file elife-109174-fig7-figsupp1-data2.zip › Figure 7-figure supplement 1-source data 2-Actin-Hsd11b1-OE.Tif]

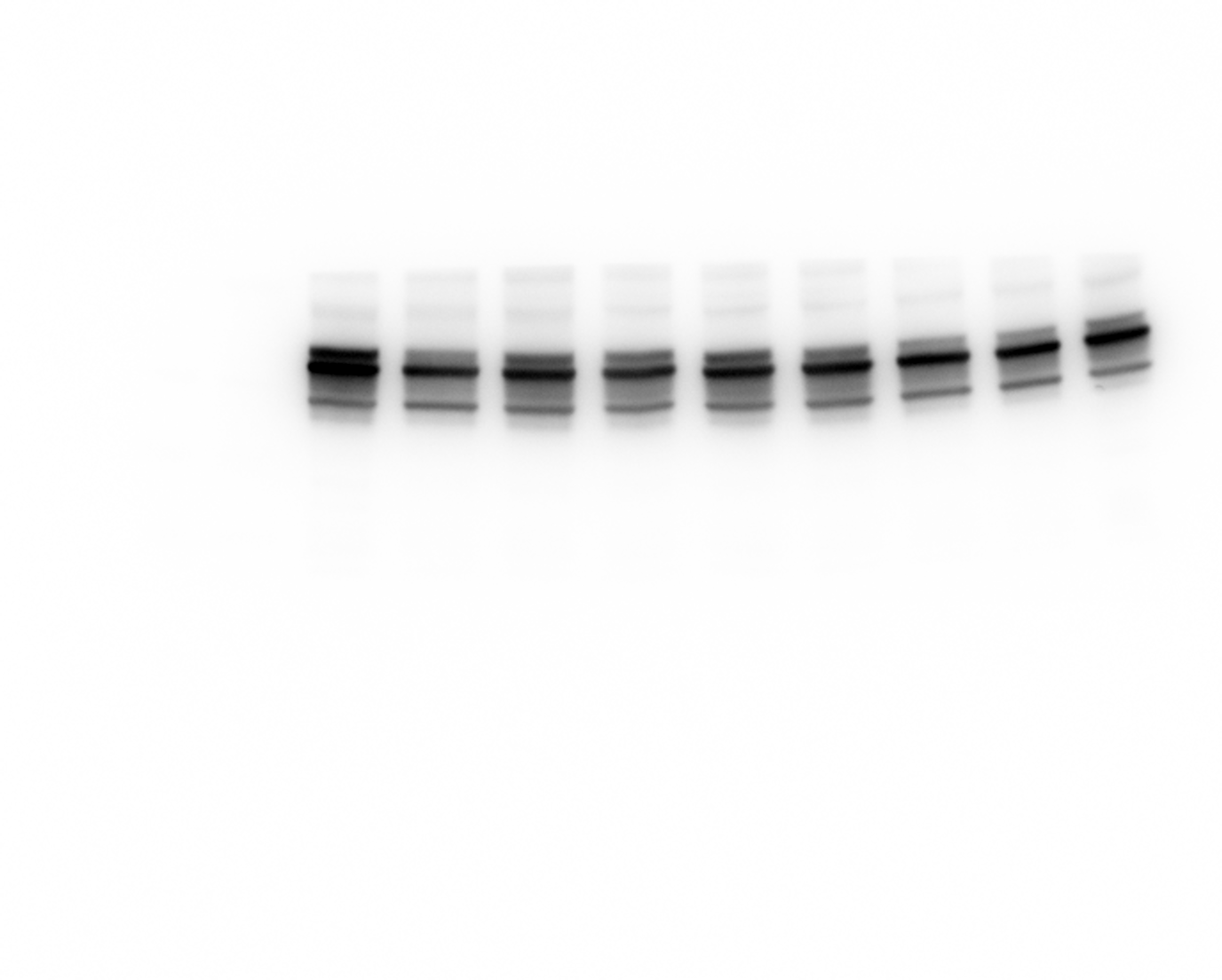

Supplement: Figure 7—figure supplement 1—source data 2. [file elife-109174-fig7-figsupp1-data2.zip › Figure 7-figure supplement 1-source data 2-Actin-Srebp2-KD.tif]

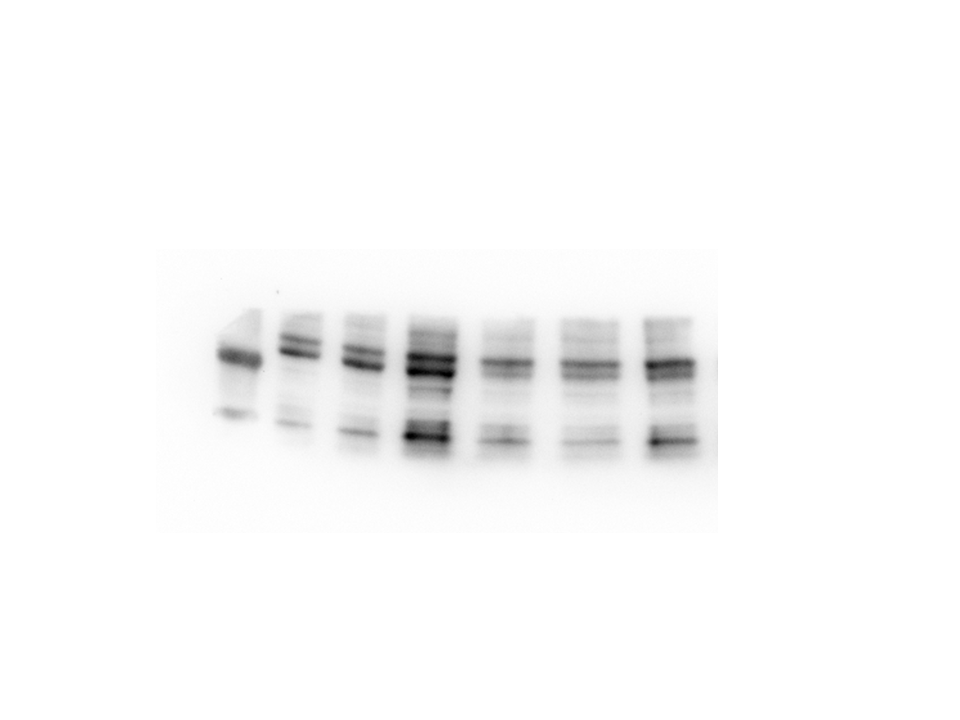

Supplement: Figure 7—figure supplement 1—source data 2. [file elife-109174-fig7-figsupp1-data2.zip › Figure 7-figure supplement 1-source data 2-Idi1-KD.tif]

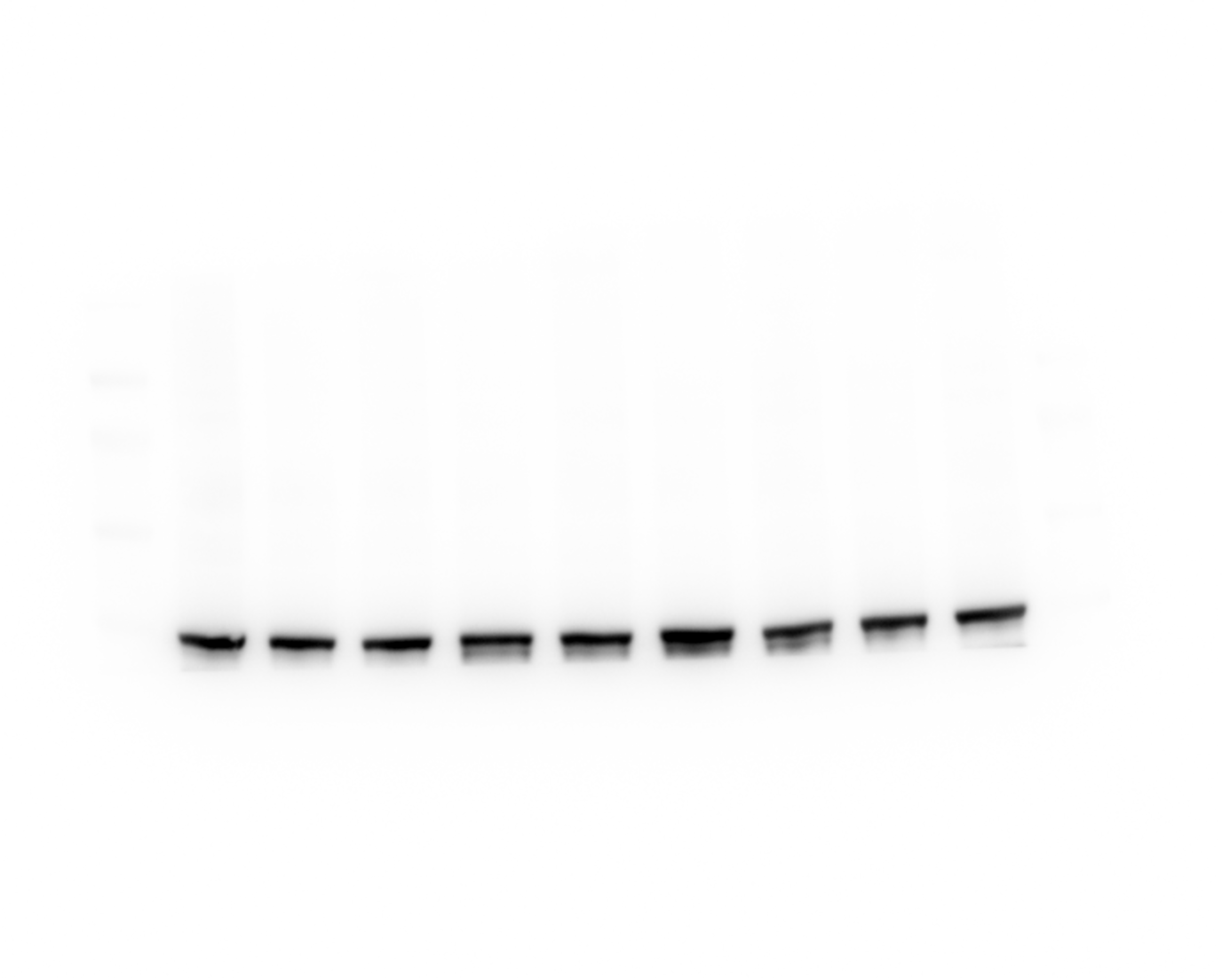

Supplement: Figure 7—figure supplement 1—source data 2. [file elife-109174-fig7-figsupp1-data2.zip › Figure 7-figure supplement 1-source data 2-Actin-Hsd11b1-KD.Tif]

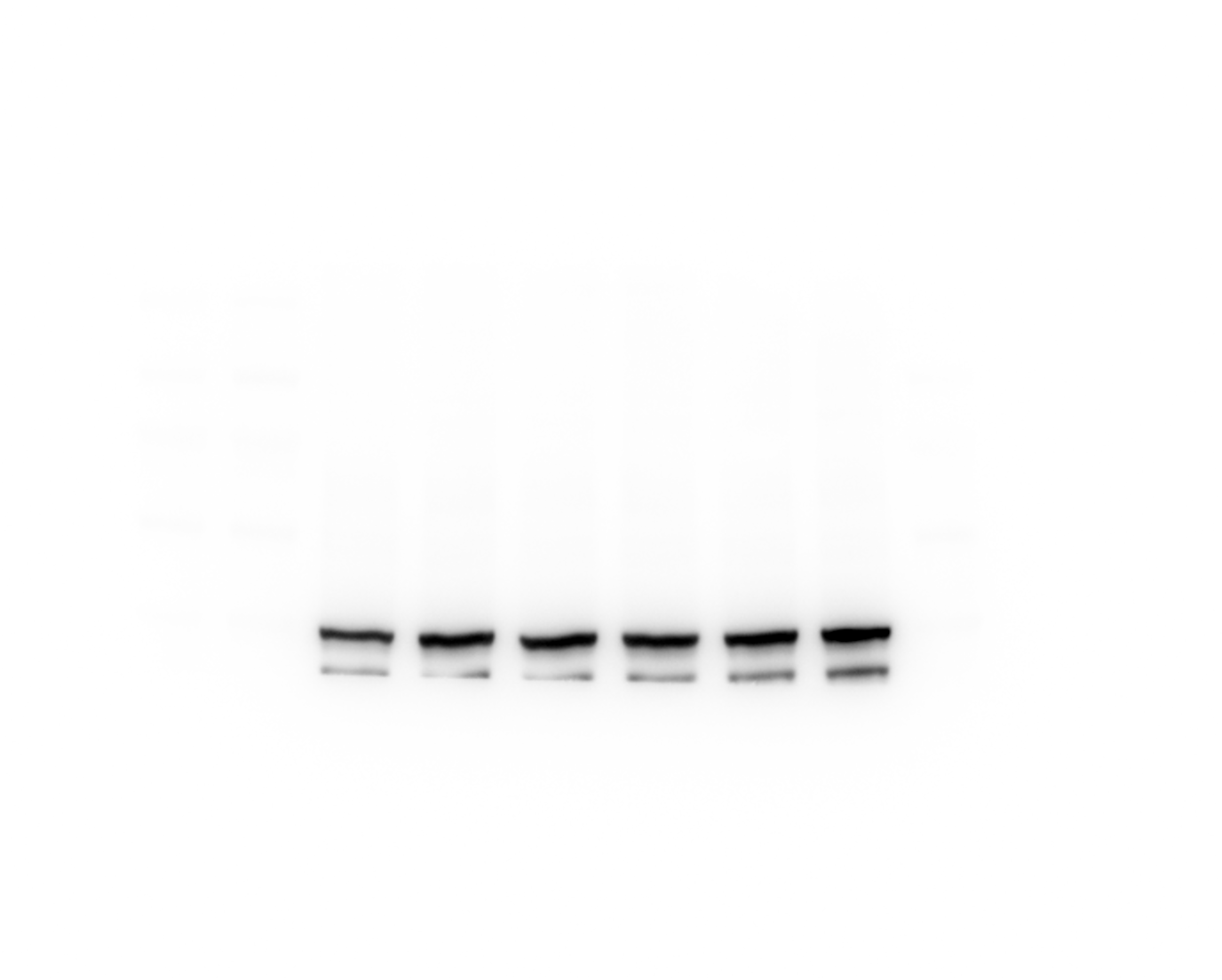

Supplement: Figure 7—figure supplement 1—source data 2. [file elife-109174-fig7-figsupp1-data2.zip › Figure 7-figure supplement 1-source data 2-Actin-Idi1-KD.Tif]

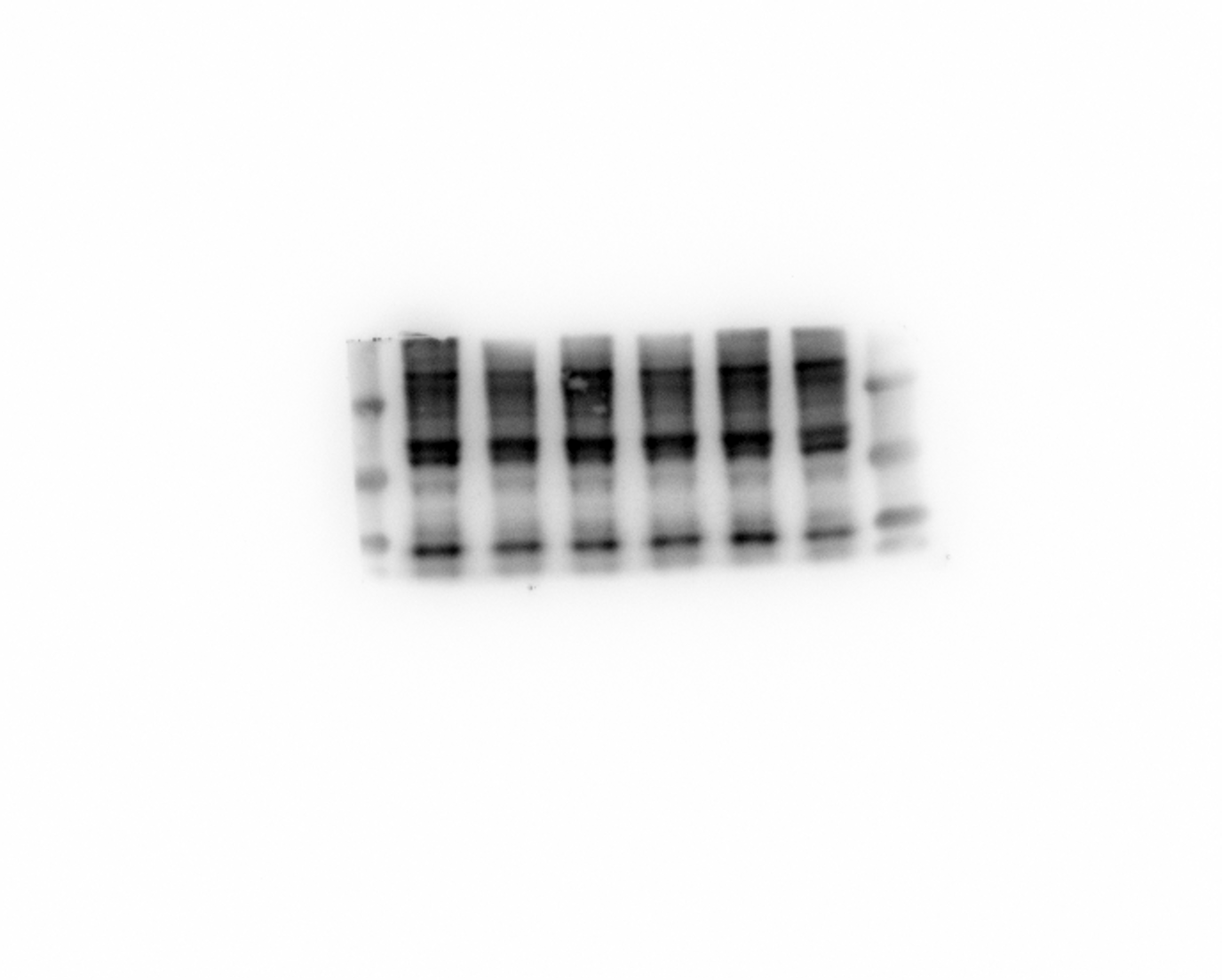

Supplement: Figure 7—figure supplement 1—source data 2. [file elife-109174-fig7-figsupp1-data2.zip › Figure 7-figure supplement 1-source data 2-Hsd11b1-OE.Tif]

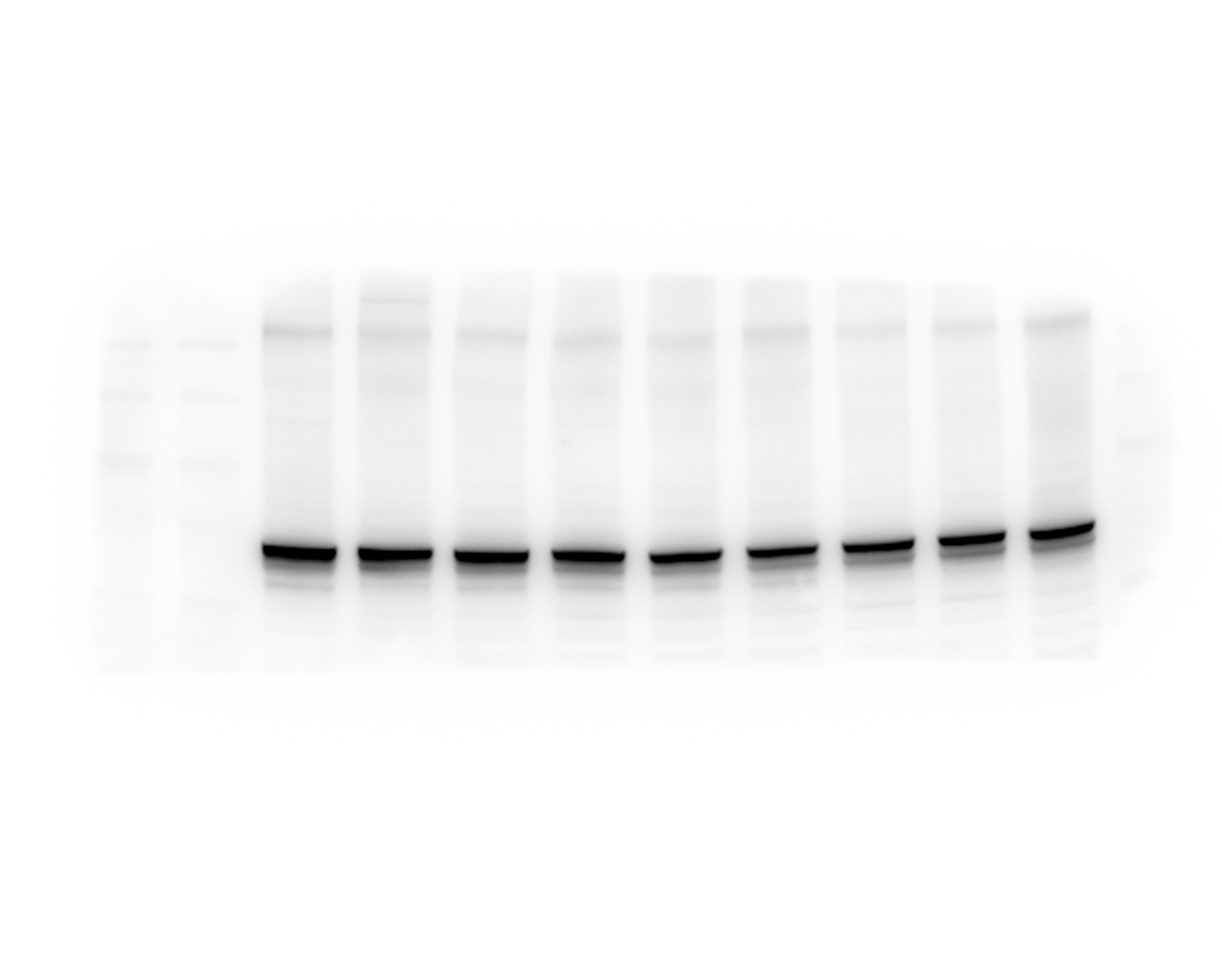

Supplement: Figure 7—figure supplement 1—source data 2. [file elife-109174-fig7-figsupp1-data2.zip › Figure 7-figure supplement 1-source data 2-Srebp2-KD.tif]
