# Supplementary material for: Magnesium isoglycyrrhizinate alleviates alcohol-associated liver disease through targeting HSD11B1
Source: eLife. 2026 Jul 28;15:RP109174. doi: 10.7554/eLife.109174 (PMC13412320; doi:10.7554/eLife.109174)
Supplement: Figure 3—figure supplement 2—source data 1. [file elife-109174-fig3-figsupp2-data1.zip › Figure 3-figure supplement 2-Source PDF.pdf]

D

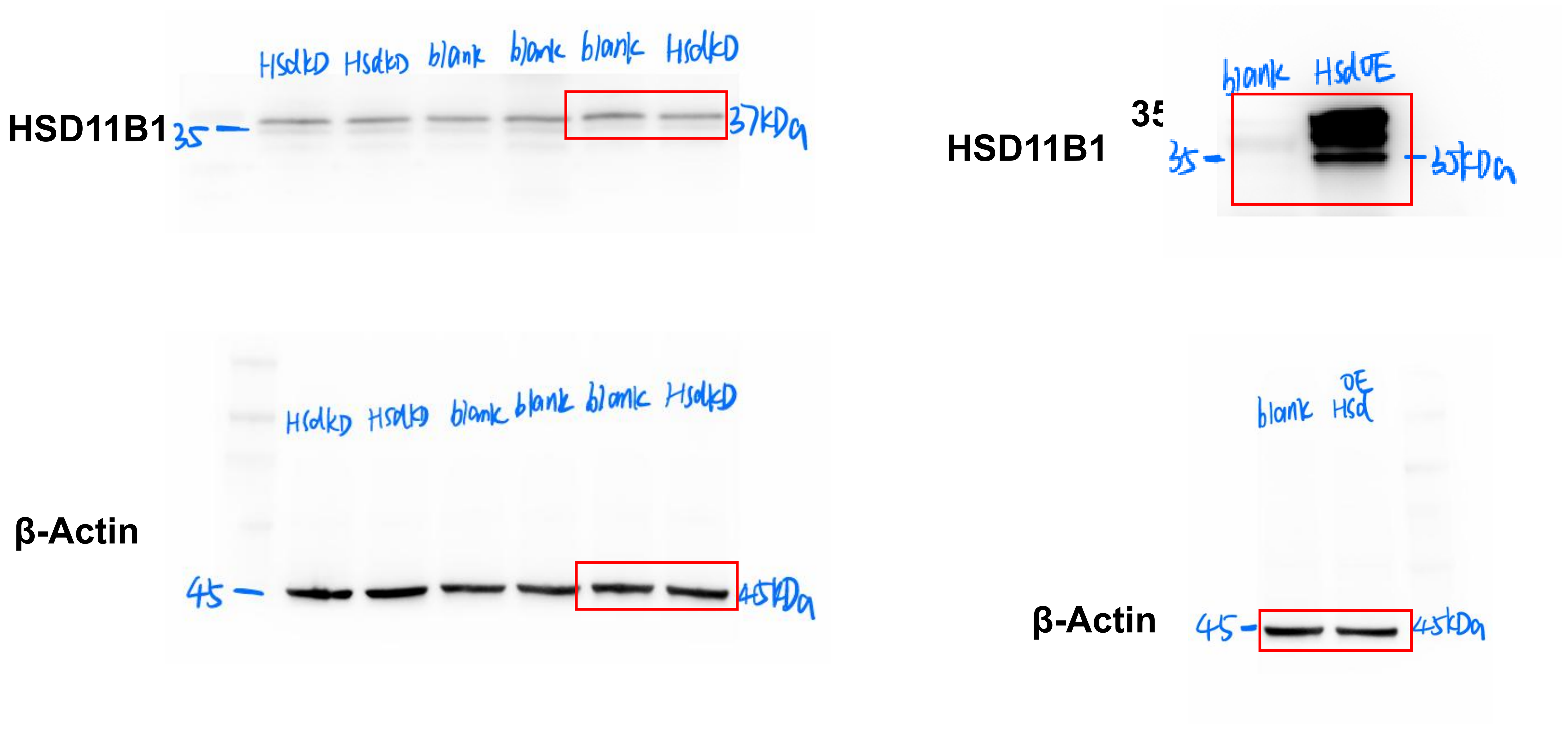

E

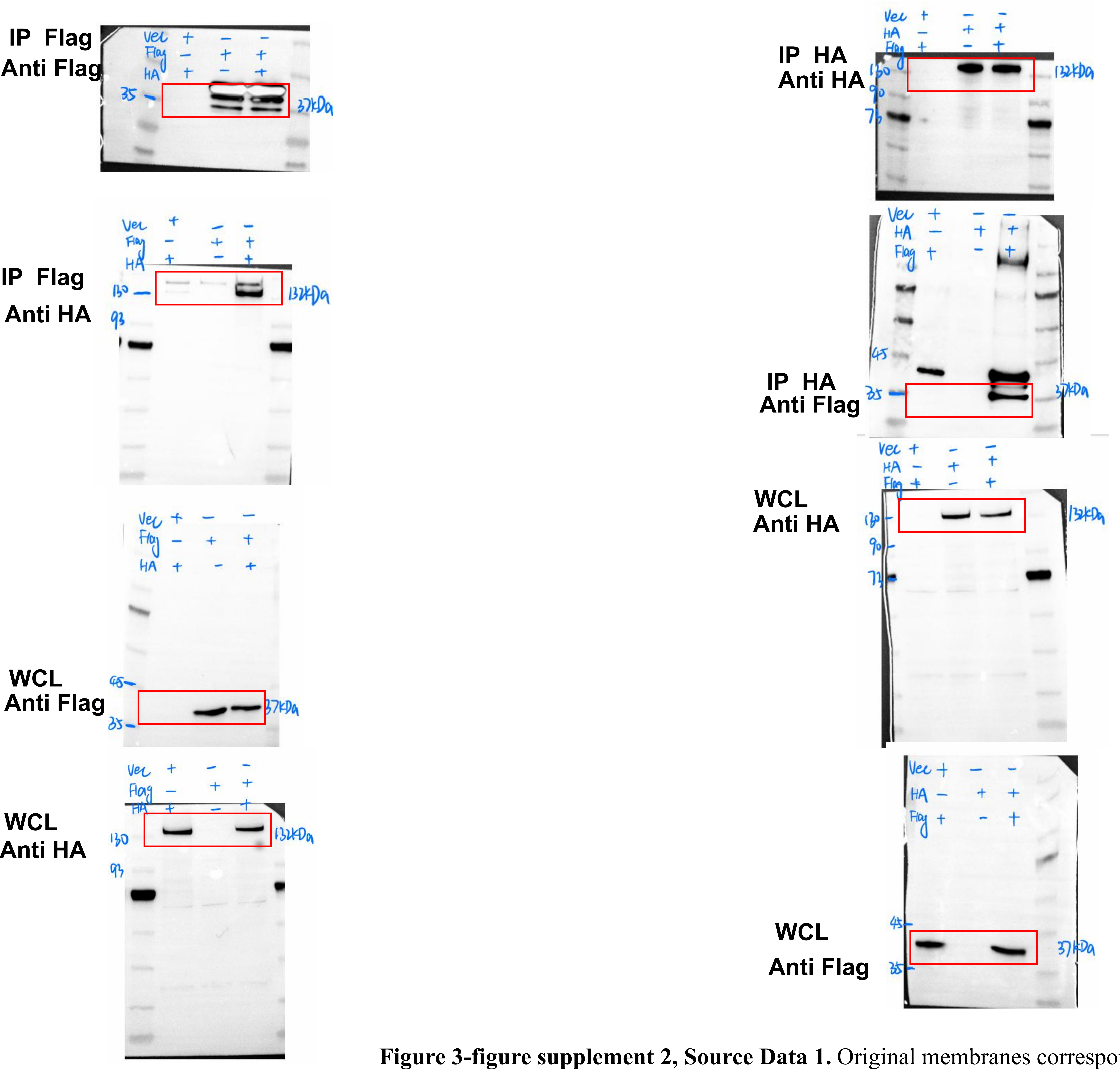

Figure 3-figure supplement 2, Source Data 1. Original membranes corresponding to Figure 3-figure supplement 2
