# Supplementary material for: Magnesium isoglycyrrhizinate alleviates alcohol-associated liver disease through targeting HSD11B1
Source: eLife. 2026 Jul 28;15:RP109174. doi: 10.7554/eLife.109174 (PMC13412320; doi:10.7554/eLife.109174)
Supplement: Figure 6—source data 1. [file elife-109174-fig6-data1.zip › Figure 6-Source PDF.pdf]

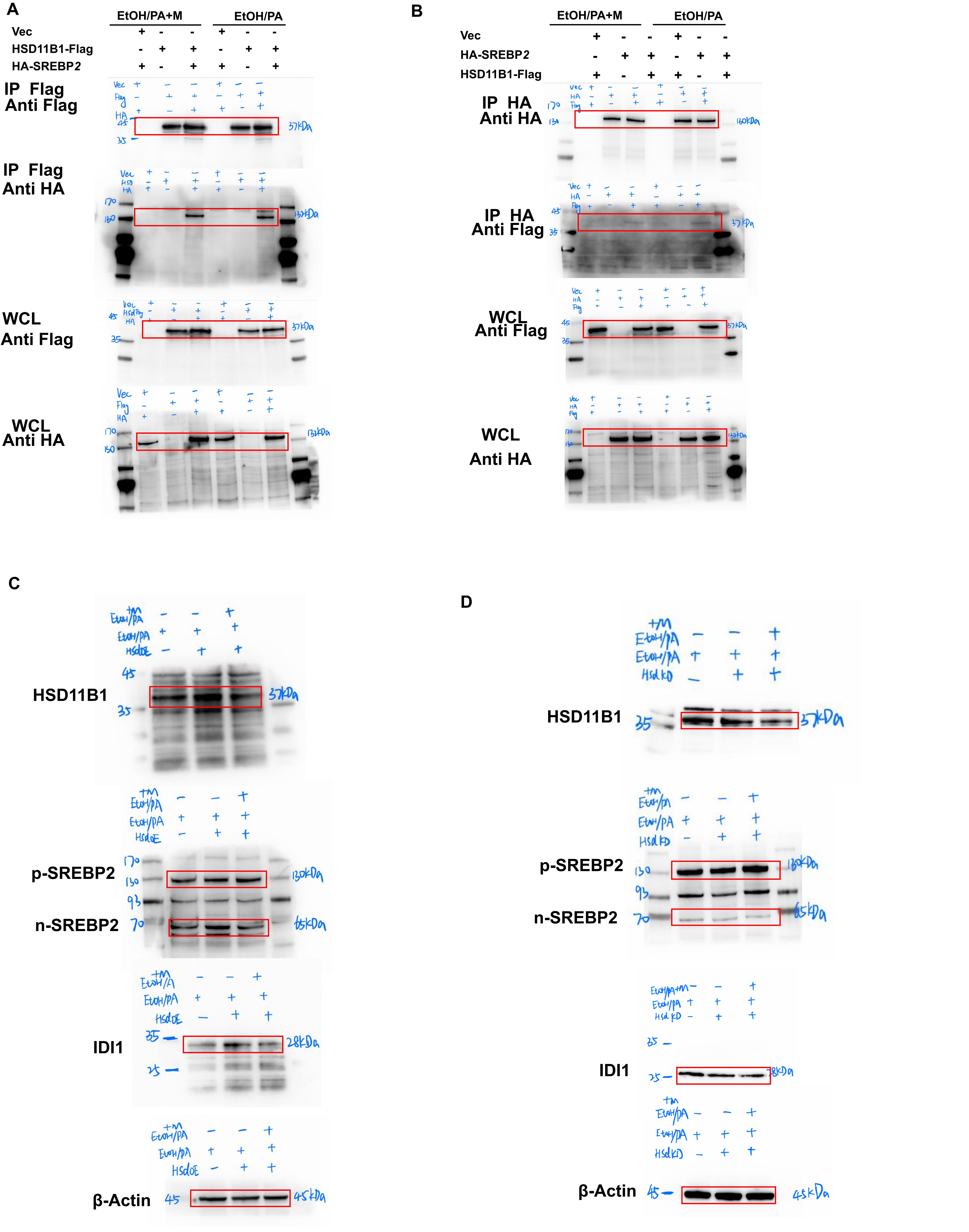


**Fig 6, Source Data 1.** Original membranes corresponding to Figure 6

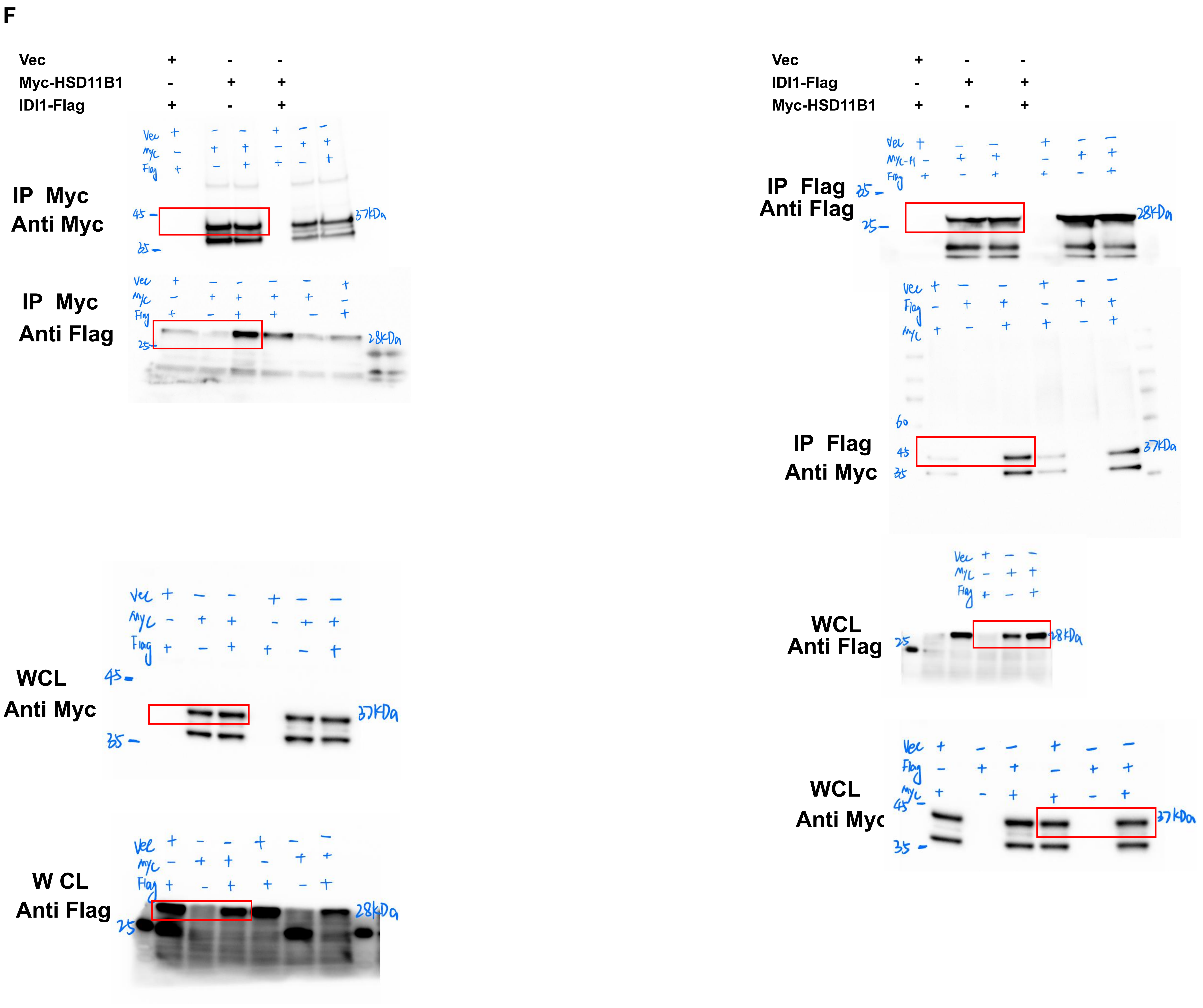


**Fig 6, Source Data 1.** Original membranes corresponding to Figure 6
