# Supplementary material for: Magnesium isoglycyrrhizinate alleviates alcohol-associated liver disease through targeting HSD11B1
Source: eLife. 2026 Jul 28;15:RP109174. doi: 10.7554/eLife.109174 (PMC13412320; doi:10.7554/eLife.109174)
Supplement: Supplementary file 2. [file elife-109174-supp2.docx]

**Supporting information**

**Magnesium isoglycyrrhizinate alleviates alcohol-associated liver disease through targeting HSD11B1**

Lu Xiao^1,2,†^, Lu Li^3,†^ , Shasha Wu^4^, Zhaoyi Che^1^, Yuyang Du^4^, Jingyi Zheng^5^, Jingsong Yan^4^, Hao Wang^1^, Hong Zhang^6^, Yan Li^5,*^, Jia Xiao^1,7,*^

^1^Clinical Medicine Research Institute and Department of Anesthesiology, The First Affiliated Hospital of Jinan University, Guangzhou 510632, China; ^2^Department of Gastroenterology, People’s Hospital of Guangming District, Shenzhen 518107, China; ^3^Department of Gastroenterology, The First Affiliated Hospital of Jinan University, Guangzhou 510632, China

^4^Department of Systems Biology, School of Life Sciences, Southern University of Science and Technology, Shenzhen 518055, China; ^5^Shenzhen Hospital of Southern Medical University, Shenzhen 518101, China; ^6^Department of Interventional Radiology and Vascular Surgery, The Sixth Affiliated Hospital of Jinan University, Dongguan 523573, China; ^7^School of Life and Health Sciences, University of Health and Rehabilitation Sciences, Qingdao, 266071, China

shRNA sequences

| Gene | Sequences |
| --- | --- |
| *Hsd11b1* | GAATTCTGACATTTGCGGAGCAATTTACTCGAGTAAATTGCTCCGCAAATGTCATTTTTTGGATCC |
| *Srebp2* | GAATTCGCGGACAACACACAATATCATCTCGAGATGATATTGTGTGTTGTCCGCTTTTTTGGATCC |
| *Idi1* | GAATTCTAGCGGAGATGTGTATTCTTACTCGAGTAAGAATACACATCTCCGCTATTTTTTGGATCC |
