# Supplementary material for: Magnesium isoglycyrrhizinate alleviates alcohol-associated liver disease through targeting HSD11B1
Source: eLife. 2026 Jul 28;15:RP109174. doi: 10.7554/eLife.109174 (PMC13412320; doi:10.7554/eLife.109174)
Supplement: Supplementary file 5. [file elife-109174-supp5.docx]

**Supporting information**

**Magnesium isoglycyrrhizinate alleviates alcohol-associated liver disease through targeting HSD11B1**

Lu Xiao^1,2,†^, Lu Li^3,†^ , Shasha Wu^4^, Zhaoyi Che^1^, Yuyang Du^4^, Jingyi Zheng^5^, Jingsong Yan^4^, Hao Wang^1^, Hong Zhang^6^, Yan Li^5,*^, Jia Xiao^1,7,*^

^1^Clinical Medicine Research Institute and Department of Anesthesiology, The First Affiliated Hospital of Jinan University, Guangzhou 510632, China; ^2^Department of Gastroenterology, People’s Hospital of Guangming District, Shenzhen 518107, China; ^3^Department of Gastroenterology, The First Affiliated Hospital of Jinan University, Guangzhou 510632, China

^4^Department of Systems Biology, School of Life Sciences, Southern University of Science and Technology, Shenzhen 518055, China; ^5^Shenzhen Hospital of Southern Medical University, Shenzhen 518101, China; ^6^Department of Interventional Radiology and Vascular Surgery, The Sixth Affiliated Hospital of Jinan University, Dongguan 523573, China; ^7^School of Life and Health Sciences, University of Health and Rehabilitation Sciences, Qingdao, 266071, China

Daily body weight of mice in each experimental group during the study period

| **Time (day)** | **Ctrl (g)** | | | | | | **EtOH (g)** | | | | | | **EtOH + M 25 mg/kg (g)** | | | | | | **EtOH + M 50 mg/kg (g)** | | | | | |
| --- | --- | --- | --- | --- | --- | --- | --- | --- | --- | --- | --- | --- | --- | --- | --- | --- | --- | --- | --- | --- | --- | --- | --- | --- |
|  |  |  |  |  |  | **mean±SD** |  |  |  |  |  | **mean±SD** |  |  |  |  |  | **mean±SD** |  |  |  |  |  | **mean±SD** |
| **1** | **21.7** | **21.1** | **23.1** | **21.6** | **22.5** | **22.00 ± 0.79** | **21.3** | **22.2** | **21.7** | **22.7** | **23.5** | **22.28 ± 0.86** | **22.7** | **21.9** | **22.6** | **22.3** | **23.4** | **22.58 ± 0.55** | **21.8** | **22.7** | **21.8** | **22.4** | **22.9** | **22.32 ± 0.51** |
| **2** | **22.1** | **22.8** | **23.8** | **21.9** | **22.6** | **22.64 ± 0.74** | **21.4** | **22.7** | **22.8** | **23.8** | **24.3** | **23.00 ± 1.12** | **23.1** | **22.8** | **23.1** | **22.8** | **23.8** | **23.12 ± 0.41** | **22.4** | **23.1** | **22.8** | **22.8** | **23.6** | **22.94 ± 0.45** |
| **3** | **22.5** | **23.1** | **23.6** | **22.2** | **22.9** | **22.86 ± 0.54** | **21.2** | **23** | **22.6** | **24** | **24.4** | **23.04 ± 1.26** | **23.4** | **23.1** | **23.2** | **23** | **23.2** | **23.18 ± 0.14** | **23** | **23.7** | **23.1** | **22.9** | **23** | **23.14 ± 0.32** |
| **4** | **23.6** | **23.3** | **24.3** | **23.8** | **23.1** | **23.62 ± 0.47** | **21.9** | **23.3** | **23.7** | **23.7** | **24.8** | **23.49 ± 1.04** | **23.5** | **22.7** | **22.6** | **23.7** | **23.3** | **23.16 ± 0.49** | **23.6** | **24** | **22.9** | **23.9** | **22.8** | **23.44 ± 0.56** |
| **5** | **24** | **23.8** | **24.8** | **23.8** | **23.4** | **23.96 ± 0.52** | **22** | **23.5** | **24.5** | **23.8** | **24.4** | **23.64 ± 1.00** | **24.1** | **22.7** | **23.2** | **23.8** | **23.5** | **23.46 ± 0.54** | **23.1** | **23.8** | **22.9** | **23.5** | **22.7** | **23.60 ± 0.45** |
| **6** | **23.9** | **23.8** | **25.5** | **24.4** | **23.2** | **24.16 ± 0.86** | **22.3** | **23.8** | **24.3** | **24.3** | **24.3** | **23.80 ± 0.87** | **24.3** | **23.1** | **22.7** | **24.3** | **24** | **23.68 ± 0.74** | **23.3** | **23.9** | **23.2** | **23.4** | **22.4** | **23.24 ± 0.54** |
| **7** | **24.7** | **23.8** | **25.3** | **24.2** | **23.7** | **24.34 ± 0.67** | **22.2** | **24** | **24.4** | **23.6** | **23.6** | **23.56 ± 0.83** | **23.3** | **22.1** | **22.3** | **23.6** | **24.2** | **23.10 ± 0.88** | **22.5** | **23.6** | **23.2** | **23.6** | **22.2** | **23.02 ± 0.64** |
| **8** | **24.6** | **24.2** | **25.1** | **24.5** | **23.6** | **24.40 ± 0.55** | **22.4** | **24.2** | **24.1** | **23** | **23.1** | **23.36 ± 0.77** | **23** | **21.4** | **21.8** | **23** | **24.9** | **22.82 ± 1.36** | **23** | **22.9** | **23.5** | **23.5** | **22.5** | **23.08 ± 0.43** |
| **9** | **25** | **24.3** | **25.3** | **24.4** | **24** | **24.60 ± 0.53** | **22.2** | **24.7** | **23.5** | **23.3** | **23.3** | **23.40 ± 0.89** | **23** | **20.8** | **21.6** | **23.3** | **24.4** | **22.62 ± 1.42** | **22.6** | **23** | **23.3** | **23.7** | **22.3** | **22.98 ± 0.55** |
| **10** | **24.7** | **24.3** | **25.1** | **24.6** | **24.3** | **24.60 ± 0.33** | **22.3** | **24.5** | **24** | **23.2** | **22.5** | **23.30 ± 0.94** | **22.5** | **21** | **21.3** | **23.2** | **24.1** | **22.42 ± 1.29** | **22.4** | **22.8** | **23.4** | **23.6** | **22.6** | **22.96 ± 0.52** |
| **11** | **25.1** | **24.4** | **25.1** | **25.3** | **24.7** | **24.92 ± 0.36** | **22.1** | **24.3** | **23.8** | **23** | **22.2** | **23.08 ± 0.96** | **22.5** | **21** | **21.4** | **23** | **24** | **22.38 ± 1.21** | **22.5** | **22.7** | **23.3** | **23.5** | **22.5** | **22.90 ± 0.47** |
| **12** | **24.2** | **24.7** | **25.2** | **25.1** | **24.8** | **24.80 ± 0.39** | **22.2** | **24.2** | **23.6** | **23** | **22.4** | **23.08 ± 0.83** | **22.4** | **20.5** | **21.3** | **23** | **23.9** | **22.22 ± 1.34** | **22.8** | **22.9** | **22.9** | **23.7** | **22.8** | **23.02 ± 0.38** |
| **13** | **24.5** | **24.5** | **25.6** | **25.7** | **25.1** | **25.08 ± 0.58** | **22.4** | **24.5** | **23.5** | **22.7** | **22** | **23.02 ± 0.99** | **22.6** | **20.8** | **21.4** | **22.7** | **23.9** | **22.28 ± 1.21** | **22.7** | **22.7** | **23.5** | **23.9** | **23.2** | **23.20 ± 0.52** |
| **14** | **25.3** | **24.4** | **25.4** | **25.5** | **25.2** | **25.16 ± 0.44** | **22.2** | **24.2** | **23.5** | **23.2** | **22** | **23.01 ± 0.92** | **22.7** | **20.8** | **21.6** | **23.2** | **24** | **22.46 ± 1.27** | **22.8** | **23** | **23.6** | **23.8** | **23.1** | **23.26 ± 0.42** |
| **15** | **25.2** | **25.8** | **25.8** | **25.6** | **25.4** | **25.56 ± 0.26** | **22.1** | **23.8** | **23.4** | **22.8** | **21.9** | **22.80 ± 0.82** | **22.5** | **20.8** | **21.3** | **22.8** | **23.8** | **22.24 ± 1.20** | **23.2** | **23.1** | **23.7** | **23.6** | **23.2** | **23.36 ± 0.27** |
