# Supplementary material for: Magnesium isoglycyrrhizinate alleviates alcohol-associated liver disease through targeting HSD11B1
Source: eLife. 2026 Jul 28;15:RP109174. doi: 10.7554/eLife.109174 (PMC13412320; doi:10.7554/eLife.109174)
Supplement: Peer review history

# Editor report: eLife Assessment

**DOI:** 10.7554/eLife.109174.4.sa0

**Authors:** Satyajit Rath

**Affiliations:** National Institute of Immunology India

**Keywords:** Convincing, Important

This **important** study reports characterisation of hepatocyte molecular pathways affected by a glycyrrhizin derivative in both in vivo and in vitro mouse models of alcohol-associated liver disease. The authors show **convincing** evidence indicating that IPP delta isomerase 1 (Idi1) is an intermediate in these pharmacological effects, via the binding of the glycyrrhizin derivative to an upstream regulator of Idi1, HSD11B1. The findings would be of interest to immunologists and pharmacologists interested in liver inflammation and its amelioration.

# Referee report: Reviewer #1 (Public review)

**DOI:** 10.7554/eLife.109174.4.sa1

Summary:

In this article by Xiao et al. the authors aimed to identify the precise targets by which magnesium isoglycyrrhizinate (MgIG) functions to improve liver injury in response to ethanol treatment. The authors found through a series of in-vivo and molecular approaches that MgIG treatment attenuates alcohol-induced liver injury through a potential SREBP2-IdI1 axis. The revised manuscript adds to a previous set of literature showing MgIG improves liver function across a variety of etiologies, and also provides mechanistic insight into its mechanism of action. All major weaknesses were addressed in the revised submission.

Strengths:

(1) The authors use a combination of approaches from both in-vivo mouse models to in-vitro approaches with AML12 hepatocytes to support the notion that MgIG does improve liver function in response to ethanol treatment.

(2) The authors use both knockdown and overexpression approaches, in-vivo and in-vitro, to support most of the claims provided.

(3) Identification of HSD11B1 as the protein target of MgIG, as well as confirmation of direct protein-protein interactions between HSD11B1/SREBP2/IDI1 is novel.

Comments on revision:

The authors addressed all my concerns. No additional comments.

# Referee report: Reviewer #2 (Public review)

**DOI:** 10.7554/eLife.109174.4.sa2

Summary:

In this manuscript, the authors investigated magnesium isoglycyrrhizinate (MgIG)'s hepatoprotective actions in chronic-binge alcohol-associated liver disease (ALD) mouse models and ethanol/palmitic acid-challenged AML-12 hepatocytes. They found that MgIG markedly attenuated alcohol-induced liver injury, evidenced by ameliorated histological damage, reduced hepatic steatosis, and normalized liver-to-body weight ratios. RNA sequencing identified isopentenyl diphosphate delta isomerase 1 (IDI1) as a key downstream effector. Hepatocyte-specific genetic manipulations confirmed that MgIG modulates the SREBP2-IDI1 axis. The mechanistic studies suggested that MgIG could directly target HSD11B1 and modulate the HSD11B1-SREBP2-IDI1 axis to attenuate ALD. This manuscript is of interest to the research field of ALD.

Strengths:

The authors have performed both in vivo and in vitro studies to demonstrate the action of magnesium isoglycyrrhizinate on hepatocytes and an animal model of alcohol-associated liver disease.

My first question: All the treatment arms (A-control, MgIG-25 mg/kg, MgIG-50 mg/kg) showed significant body weight loss compared to the untreated controls (Supplemental Figure 1A), but the body weight significantly increased in the treatment arms (A-control and MgIG-50 mg/kg) compared to the untreated controls (Figure 1E). Why?

My second question: Mice with MgIG (25 mg/kg) showed the lowest body weight, compared to either A-control or MgIG (50 mg/kg) treatment. According to the authors' explanation, the MgIG (25 mg/kg) caused bodyweight loss are attributed to inter-individual variability, differences in metabolic adaptation, or sample size-related variation. Did these differences happen in MgIG (25 mg/kg) only? or in all other groups? The mouse group assignment should be randomized; however, a large variation in bodyweight was seen in MgIG (25 mg/kg) group. It is not convincing for the author to select MgIG (50 mg/kg) group for subsequent animal experiments, because of a large variation in MgIG (25 mg/kg) group, and because that MgIG (50 mg/kg) group demonstrated more consistent and stable improvements across multiple parameters. The author should reanalyze and compare all the raw data between MgIG (50 mg/kg) group and MgIG (25 mg/kg) group, and address the issues being pointed out and justify rationale for the animal group assignment.

The author's response did not answer my question. If the authors believe it could be experimental constraints associated with the MgIG formulation, then it is questionable for this MgIG formulation used in all other associated experiments. The experiments, at least those the MgIG formulation associated experiments, need to be repeated.

The author explained the relative expression was normalized to GAPDH (fold change), but they did not answer my question. My question is for Figure 5B. in Figure 5B (left, Hsd11b1-KD), scramble control showed over 100 (unit), however, in Figure 5B (right, Hsd11b1-OE), scramble control showed only 0.5-1 (unit). The data seemed that authors used same scramble control for both KD and OE? If yes, they should provide more details of the KD and OE experiments and explain why this happened. If they used plasmid for OE control, they also need to clarify it. In addition, qPCR is not a good assay to show the success of KD or OE, Western blotting should be done as convincing data to show the success of KD or OE.

Comments on revised version.

In this revision, all the issues are addressed.

# Author comment: Author response

**DOI:** 10.7554/eLife.109174.4.sa3

**Authors:** Lu Xiao, Lu Li, Shasha Wu, Zhaoyi Che, Yuyang Du, Jingyi Zheng, Jingsong Yan, Hao Wang, Hong Zhang, Yan Li, Jia Xiao

**Affiliations:** First Affiliated Hospital of Jinan University Guangzhou China; First Affiliated Hospital of Jinan University Guangzhou China; Southern University of Science and Technology Shenzhen China; First Affiliated Hospital of Jinan University Guangzhou China; Southern University of Science and Technology Shenzhen China; Southern Medical University Shenzhen Hospital Shenzhen China; Southern University of Science and Technology Shenzhen China; First Affiliated Hospital of Jinan University Guangzhou China; Jinan University Dongguan China; Southern Medical University Shenzhen Hospital Shenzhen China; University of Health and Rehabilitation Sciences Qingdao China

The following is the authors’ response to the previous reviews

**Public Reviews:**

**Reviewer #1 (Public review):**

The authors addressed all my concerns.

We sincerely appreciate your recognition of our efforts to address the reviewers' suggestions and improve the manuscript.

**Reviewer #2 (Public review):**

(1) All the treatment arms (A-control, MgIG-25 mg/kg, MgIG-50 mg/kg) showed significant body weight loss compared to the untreated controls (Supplemental Figure 1A), but the body weight significantly increased in the treatment arms (A-control and MgIG-50 mg/kg) compared to the untreated controls (Figure 1E). Why?

We appreciate the reviewer’s careful observation regarding the apparent discrepancy between Supplemental Figure 1A and Figure 1E. We apologize for any confusion caused by the presentation of these data.

We would like to clarify that Supplemental Figure 1A and Figure 1E represent two different parameters. Supplemental Figure 1A shows absolute body weight, whereas Figure 1E presents the liver-to-body weight ratio (LW/BW), as indicated in the revised figure legend.

In the NIAAA alcohol-fed model, chronic ethanol exposure typically results in reduced body weight gain or relative body weight loss compared with normal diet-fed control mice, which is consistent with the findings shown in Supplemental Figure 1A. In the preliminary dose-finding study, all alcohol-fed groups (EtOH groups, MgIG 25 mg/kg, and MgIG 50 mg/kg) exhibited lower absolute body weight compared with the untreated control group, which is a common feature of ethanol-induced liver injury models.

By contrast, Figure 1E reflects changes in the LW/BW ratio rather than total body weight. Ethanol feeding induces hepatomegaly and hepatic steatosis, thereby increasing the LW/BW ratio. Although the LW/BW ratio in the MgIG-treated group remained higher than that in the untreated control group, MgIG treatment significantly reduced the ethanol-induced increase in LW/BW ratio compared with the EtOH group, consistent with its hepatoprotective effects and reduced hepatic lipid accumulation. We hope this clarification could well answer this concern. Thank you very much!

(2) Mice with MgIG (25 mg/kg) showed the lowest body weight, compared to either A-control or MgIG (50 mg/kg) treatment. According to the authors' explanation, the MgIG (25 mg/kg) caused bodyweight loss are attributed to inter-individual variability, differences in metabolic adaptation, or sample size-related variation. Did these differences happen in MgIG (25 mg/kg) only? or in all other groups? The mouse group assignment should be randomized; however, a large variation in bodyweight was seen in MgIG (25 mg/kg) group. It is not convincing for the author to select MgIG (50 mg/kg) group for subsequent animal experiments, because of a large variation in MgIG (25 mg/kg) group, and because that MgIG (50 mg/kg) group demonstrated more consistent and stable improvements across multiple parameters. The author should reanalyze and compare all the raw data between MgIG (50 mg/kg) group and MgIG (25 mg/kg) group, and address the issues being pointed out and justify rationale for the animal group assignment.

We appreciate the reviewer’s careful evaluation regarding the variability observed in the MgIG (25 mg/kg) group and the rationale for dose selection.

Supplemental Figure 1A presents data from our preliminary dose-finding study (n=5 per group, independent cohort), in which all alcohol-fed groups showed expected body weight loss relative to the normal-diet control, as is typical in the NIAAA model. The 25 mg/kg group exhibited numerically greater variability (likely due to inter-individual metabolic differences and small sample size), but no statistically significant difference was observed among the three alcohol-fed groups (A-control, 25 mg/kg, and 50 mg/kg) in final body weight (one-way ANOVA with post-hoc test).

Mice were randomized by initial body weight and age prior to diet feeding. To address the reviewer’s concern, we have now included Supplementary Table Body weight-raw data with individual animal body weight data (raw values, mean ± SD) for both the dose-finding and main experiments, together with statistical comparisons. We selected 50 mg/kg for all subsequent experiments because it provided more consistent and statistically significant improvements across multiple key parameters (ALT, AST, TG, TC, NAS score, Oil Red O staining, and LW/BW ratio) compared with 25 mg/kg. The 25 mg/kg group showed greater variability in several indices, which is why it was not chosen for mechanistic studies.

To further clarify this point, we have added detailed descriptions of the randomization procedure and dose-selection rationale in the revised Methods section. Please refer to Page 5, line 106-108 and Page 10, line 276-277. In addition, we will provide the original data on mouse body weight changes, together with the corresponding statistical analyses, in the supplementary materials to further enhance transparency and facilitate reference.

(3) The author's response did not answer my question. If the authors believe it could be experimental constraints associated with the MgIG formulation, then it is questionable for this MgIG formulation used in all other associated experiments. The experiments, at least those the MgIG formulation associated experiments, need to be repeated.

We sincerely appreciate the reviewer’s concern regarding the potential impact of the MgIG formulation on the reliability of the associated experiments.

As clarified in our previous response, the commercially available MgIG preparation used in this study is a clinically approved injectable formulation (5 mg/mL). During the preliminary in vitro dose-ranging experiments, achieving the highest testing concentration (1.0 mg/mL) required the addition of a relatively larger volume of stock solution, which slightly reduced the effective culture medium volume and may have contributed to minor effects on cell status. Consistently, CCK-8 and LDH assays showed a slight reduction in cell viability only at the highest concentration tested.

Importantly, this phenomenon was observed exclusively in the 1.0 mg/mL group. All subsequent functional and mechanistic experiments were performed using the optimized non-toxic concentration (0.25 mg/mL), at which MgIG consistently and significantly improved IL-6, Acc1, Scd1, and other relevant parameters in a dose-dependent manner (P < 0.05), without detectable cytotoxicity.

In addition, vehicle controls with volume-matched conditions were included for the high-concentration (1 mg/mL) condition to exclude potential confounding effects caused by solvent volume differences. The protective effects observed at 0.25 mg/mL were highly reproducible and were further supported by multiple independent lines of evidence, including RNA-seq analysis, enzyme activity assays, and knockdown/overexpression experiments, all of which demonstrated consistent mechanistic trends.

Therefore, we believe that the current data obtained using the optimized concentration remain reliable and interpretable, and that the formulation-related issue observed at the highest concentration does not affect the validity of the main conclusions. Nevertheless, to further address the reviewer’s concern, we are willing to provide additional replicate data for the 1.0 mg/mL cell viability/toxicity assays, as well as repeat qPCR analyses under volume-matched vehicle control conditions in the Supplementary File . Please refer to Supplementary Figure 2E.

(4) The author explained the relative expression was normalized to GAPDH (fold change), but they did not answer my question. My question is for Figure 5B. in Figure 5B (left, Hsd11b1-KD), scramble control showed over 100 (unit), however, in Figure 5B (right, Hsd11b1-OE), scramble control showed only 0.5-1 (unit). The data seemed that authors used same scramble control for both KD and OE? If yes, they should provide more details of the KD and OE experiments and explain why this happened. If they used plasmid for OE control, they also need to clarify it. In addition, qPCR is not a good assay to show the success of KD or OE, Western blotting should be done as convincing data to show the success of KD or OE.

We apologize that our previous response did not fully clarify the details of Figure 5B. The left panel of Figure 5B shows the Hsd11b1 knockdown experiment using Hsd11b1 siRNA with scramble siRNA as the corresponding control, whereas the right panel shows the Idi1 overexpression experiment using the Idi1 expression plasmid with empty vector as the corresponding control. These are two independent experiments with separate control groups, rather than a shared scramble control. We recognize that the labeling and figure presentation may have caused confusion, we have revised the legend for Figures 3B, 3C and Figures 5B, 5C as suggested.

For both experiments, relative mRNA expression levels were normalized to GAPDH and analyzed independently using the 2^^−ΔΔCt^ method relative to their respective controls. Therefore, the numerical values shown in the two panels are not directly comparable. The apparent difference in baseline expression levels reflects independent normalization and the intrinsic expression characteristics of different genes, rather than the use of the same control group or any data inconsistency.

We have confirmed that transfection efficiencies were consistent with expectations and did not significantly affect cell viability.

We also agree with the reviewer that protein-level validation would provide stronger evidence for the success of knockdown and overexpression. Accordingly, we have performed Western blot analyses for Hsd11b1 knockdown and Idi1 overexpression and will include these data in the revised manuscript to complement the qPCR results (Please refer to revised Supplementary Figure 3C and 4D).
